# Linkage disequilibrium block single-nucleotide polymorphisms in FTO alpha ketoglutarate dependent dioxygenase gene inference with breast cancer and Type II diabetes in Pakistani female population

**DOI:** 10.1371/journal.pone.0288934

**Published:** 2023-07-20

**Authors:** Qaisar Mansoor, Muhammad Ismail, Shah Ali Ul Qader

**Affiliations:** 1 Department of Biochemistry, University of Karachi, Karachi, Pakistan; 2 Institute of Biomedical and Genetic Engineering (IBGE), Islamabad, Pakistan; The University of Lahore, PAKISTAN

## Abstract

Breast Cancer and Diabetes Mellitus are top ranked non-communicable life threatening diseases concerned in South Asia. The growing scientific clues witness the involvement of genetic variations which readily serve as risk factors for the disease onset. Comprehending the association of genetic predictors and risk factors in the conserved genome regions can help reveal underlying disease genetics and identify the sustained druggable targets. The present study aims to identify discrete inference of FTO alpha-ketoglutarate dependent dioxygenase gene linkage disequilibrium block SNPs rs9940128 and rs9939609 as prognostic genetic elements in defining the disease course either as BrC or NIDDM in Pakistani population. Clinically abreast female Breast Cancer and Type II (Non-Insulin Dependent) Diabetes Mellitus cases with the healthy controls participated in the study. The genomic study was established on the DNA of cases and controls through Tetra primers ARMS PCR and PCR-RFLP; data were analyzed statistically to reach comprehensive scientific annotation. Breast Cancer incidence was high in post menopause women. Fretful cholesterol, triglycerides, hypertension, sugar profiles and high incidence in females was evident in Type II (Non-Insulin Dependent) Diabetes Mellitus. BMI and family history were meager factor for either of the diseases. FTO gene alpha-ketoglutarate dependent dioxygenase linkage disequilibrium block Single-Nucleotide Polymorphism rs9939609 and rs9940128 threating inference was significant in the cancer and diabetes subjects correspondingly. The conclusion indicates serious clinical derailments in breast cancer and Type II (Non-Insulin Dependent) Diabetes Mellitus auxiliary to disease complication in genetically risk bearing FTO alpha-ketoglutarate dependent dioxygenase gene haplotype/linkage disequilibrium block SNPs prevailing in the affected Pakistani population. These clinical and genetic indicators could decisively be adopted in health care practice to intervene the sky rising disease incidence.

## 1. Introduction

Non-communicable diseases burden data for Breast Cancer (BrC) and Type II (Non-Insulin Dependent) Diabetes Mellitus (NIDDM)is accelerating logarithmically in South Asian region particularly in Pakistan. In 2016, 11.77% of the Pakistani population was reported stressed from NIDDM [1]. But upsettingly the prevalence was revealed much higher than previous records based on Hemoglobin A1C (HbA1c) screening [2]. Likewise the cancer incidence, mortality and prevalence; BrC has been ranked at the top in the country [3]. Prevention, management and biomedical strategies can help developing countries like Pakistan for an early diagnosis, risk assessment, combat, lower the disease burden and identify druggable targets. The developments in the field of genetics have made our understanding better for the complex diseases. Diabetes mellitus and cancer are also well studied with large-scale genome-wide association studies (GWAS) clearing the way for in depth disease triggers and pathology [4]. Strong genetic associations have been observed between the single nucleotide polymorphisms (SNPs) of FTO alpha-ketoglutarate (αKG) dependent dioxygenase gene with NIDDM and BrC conceptualizing the considerate involvement of the gene [5, 6]. The FTO gene SNPs modulated the expression of gene in context of the rs9939609 SNP risk (A) and wild (T) alleles. The risk allele was found associated with the fold rise of FTO transcript in skin fibroblasts. The findings are indicative of cis-regulatory site in intron 1 of the FTO αKG dependent dioxygenase gene [7]. The FTO SNPs also regulate gene expression at large kilobases of distance that may be related to cancer susceptibility [8]. In this regard a study showed the involvement of FTO intron 1 interaction with the promoter region of IRX3 gene present at 500kb distance and the target gene expression is modified regarded to genetic variations in FTO [9]. FTO has been reported in adipogenesis and tumorigenesis by regulating post-transcription of downstream molecular expression or by target of the mammalian target protein rapamycin (mTOR). FTO inhibitors have been reported to exhibit both anti-obesity and anti-cancer effects *in vivo* [10]. In breast cancer cells, over expression of FTO gene promotes glycolysis and modulators of PI3K/AKT signal transduction pathway [11]. The cluster of SNPs in the FTO first intron has been reported in the genetic epidemiology with ethnic specificities [12]. Recent studies refined the FTO SNPs identification in relation to increased body mass index (BMI) and cancer. The mortality rate in cancer patients has been doubled in the individuals with higher BMI in comparison to normal BMI [13]. FTO SNPs exhibits the ability to modify the binding site for transcriptional factors and regulate gene expression. We aim to investigate the association of two SNPs rs9939609 and rs9940128 in the intron 1 of FTO gene with as risk factors for clinically diagnosed NIDDM and BrC in Pakistani population for better understanding and genetic correlation of the disease and its development.

## 2. Materials and methods

### 2.1 Cases and controls

The study was approved from the ethical review committee (letter no. IBGE/IEC/12/01/22) of Institute of Biomedical and Genetic Engineering, Islamabad, Pakistan. Six hundred and fifty BrC cases and two hundred normal healthy females as control; one thousand and five NIDDM cases with five hundred healthy controls participated in this study. The sampling was done by non-probability consecutive sampling. Age, body mass index (BMI), gender, blood pressure, sugar profile, family history, tobacco and alcohol consumption were determined for NIDDM cases and controls. In addition to these parameters tumor and menopause status and age of the female at the time of first delivery were also recorded for BrC cases. 5ml peripheral blood was collected after informed consent from the cases and controls taking part in the study.

### 2.2 DNA isolation

Genomic DNA was extracted from the whole blood sample using Gene-Jet DNA Purification kit (Thermo Scientific, USA). The extracted DNA was checked for quantity and quality by Nanodrop 2000C (Thermo Scientific, USA). Dilution of each DNA sample at 25ηg/μl was prepared for downstream use.

### 2.3 Genotyping

**Fig 1:**
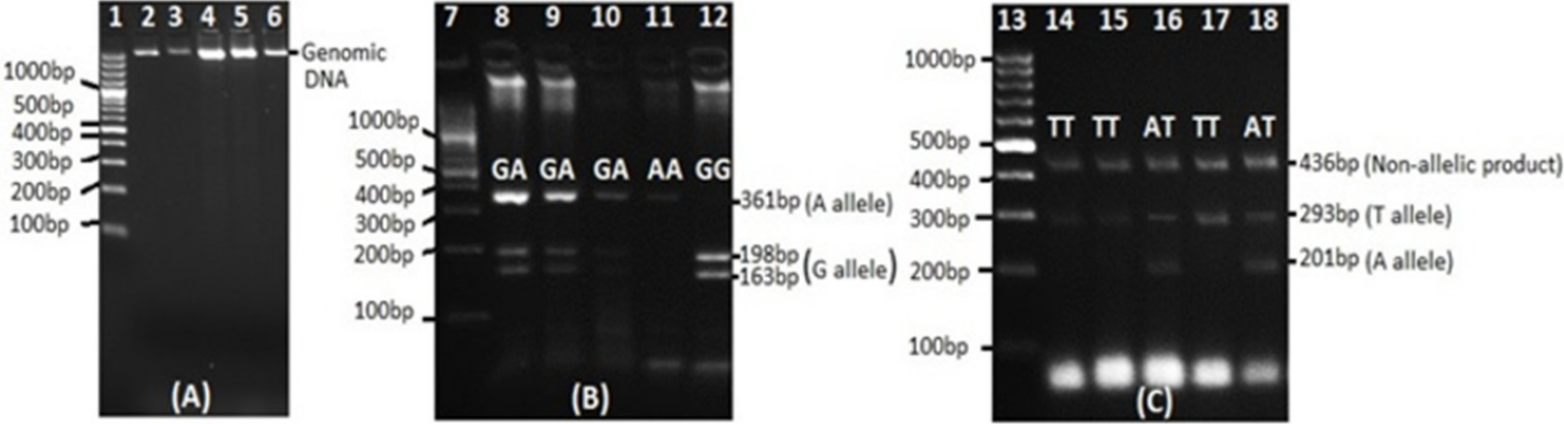
Agarose gel images. 100bp gene ruler (DNA Ladder) in lane 1, 7 and 13. (A) Genomic DNA quality check, genomic DNA in lanes 2–6 (B) rs9940128 genotyping, RFLP products in lanes 8–12, and (C) rs9939609 genotyping, Tetra-Primer ARMS-PCR products in lanes 14–18.

FTO αKG dependent dioxygenase gene SNPs rs9939609 and rs9940128 were genotyped in the sample pool by Tetra-Primer ARMS PCR and PCR-RFLP respectively. ARMS-PCR for rs9939609 was done using forward outer 5‘gttctacagttccagtcatttttgacagc’3, reverse outer 5’agcctctctaccatcttatgtccaaaca’3, risk allele ‘A’ specific forward inner 5’taggttccttgcgactgctgtgaatata’3 and wild type allele ‘T’ specific reverse inner 5’gagtaacagagactatccaagtgcatctca’3 primers. While rs9940128 PCR was carried out by specific forward 5’aggtcagggccagagataga’3 and reverse 5’gccttaggacctgaactgct’3 primers. The PCR was done using *Taq* DNA polymerase kit and dNTPs set (Thermo Scientific, USA). Briefly the 25μl PCR included 0.5U *Taq* DNA polymerase enzyme, 1X PCR buffer (NH4)_2_SO_4_, 1.5mM MgCl_2_, 20μM dNTPs, 1μM of SNP specific primers, 3ηg genomic DNA and PCR-grade water. The reaction was carried out in MultiGene OptiMax Thermal Cycler (Labnet, USA). For the rs9940128 RFLP, relevant PCR product of was digested with *Mspl* (10U/ml) (Thermo Scientific, USA) restriction enzyme as per product protocol. Amplified products of ARMS-PCR and *Msp1*digested products were subjected to electrophoresis on 2% w/v agarose gel and visualized in gel documentation system (Syngene, USA). The genotyping data was recorded from the gel analysis. DNA quality check, genotyping of rs9940128 RFLP and rs9939609 Tetra-Primer ARMS-PCR gel images are shown in Fig 1(A)–1(C) respectively.

### 2.4 Statistical analysis

Genetic association study statistical power (GASSP) and the genotype relative risk (GRR) assessment was performed to rationalize the sample size of NIDDM and BrC cases and controls [14–16]. Genotyping data was statistically analyzed by SPSS (Version 20). Chi-squared (χ^2^) test with *p* value <0.05 or = 0.05 was considered statistically significant. Odds ratio (OR) were calculated with 95% confidence interval (CI) and forest plots were constructed for genotypes and allelic data.

## 3. Results

### 3.1 Cases

The cases were presented with 33.2% male and 66.8% female NIDDM and female BrC independently along with specified healthy 29.7% male and 70.3% female controls for NIDDM and female for BrC. Mean age of all the cases was ranged 46.23 to 50.14 years with standard deviation of 9.66 to 15.63. 25.09% NIDDM and 30.6% BrC cases were obese with BMI >30, 33.45% and 13.13% were overweight respectively. The average values for fasting and random sugar profile were more than admissible range, lipid profile was normal for cholesterol and LDL but elevated for HDL and triglycerides in NIDDM cases. Hypertension of stage 1 = 20.9% and stage 2 = 51.9% was evident in the diabetic group. The BrC tumor stage 2 was most prevalent i.e., 28.2% followed by 19.2% stage 3 and less abundant stage 1 and 4. However 42.9% cases were undetermined of stage category. Almost 62% BrC cases were diagnosed for the cancer after menopause. These attributes along with tobacco and alcohol consumption, age at first delivery (BrC cases and BrC controls) and family history are shown in Table 1.

**Table 1:** Cases and controls physical and clinical features.

Parameter	Description	BrC Cases n = 650	BrC Controls n = 200	NIDDM Cases n = 1005	NIDDM Controls n = 500
Age		48.56 ± 15.54	47.23 ± 14.63	50.14 ± 11.82	49.34 ± 9.66
Gender	Female	100%	100%	66.8%	70.3%
	Male	0%	0%	33.2%	29.7%
Ethnic origin		Pakistani			
BMI (Kg/m^2^)	Normal (18.5–25)	56.27%	97.4%	41.46%	100%
	Overweight (25.1–29.9)	13.13%	2.6%	33.45%	0%
	Obese (>30)	30.6%	0%	25.09%	0%
Sugar Profile (mg/dL)	Fasting			188.66 ± 87.891	< 99
	Random			257.21 ± 96.884	125–140
Lipid Profile	Cholesterol			182.12 ± 45.60	< 200
	Triglycerides			204.19 ± 137.2	< 150
	HDL			42.78 ± 22.60	> 60
	LDL			102.92 ± 36.16	< 130
Blood Pressure (mmHg)	Normal Systolic < 120 Diastolic < 80	97.6%	94.3%	21.4%	85.7%
	Elevated Systolic 120–129 Diastolic <80	2.4%	5.7%	5.8%	14.3%
	Hypertension Stage 1 Systolic 130–139 Diastolic 80–89	0%	0%	20.9%	0%
	Hypertension Stage 2 Systolic >140 Diastolic >90	0%	0%	51.9%	0%
Tumor status	Undetermined	42.9%	Nil		
	Stage 1	5.5%			
	Stage 2	28.2%			
	Stage 3	19.2%			
	Stage 4	4.2%			
Menopausal status	Pre-menopause	37.81%	41.25%		
	Post-menopause	62.19%	58.75%		
Tobacco Consumption	Smoker	13.00%	1.5%	7%	0%
	Non smoker	87.00%	98.5%	93%	100%
Alcohol Consumption	Drinker	0%	0%	0%	0%
	Non drinker	100%	100%	100%	100%
Age at first delivery (mean)		22.10 years	23 years	-	-
Family history	Present	16.3%	0.40%	23%	2%
	Absent	83.7%	99.60%	77%	98%

### 3.2 GASSP and GRR

#### 3.2.1 NIDDM

One stage design of NIDDM 1005 cases and 500 controls (case/control ratio = 0.498) for rs9939609 has a genetic association study statistical power 80% to find disease predisposing variant with Genotype Relative Risk (GRR) 1.4475 (>1), prevalence 0.25 (0.0001–0.9999), risk allele frequency for cases 0.482 and controls 0.381 and 80% power to find disease predisposing variant of rs9940128 with GRR 1.4430 (>1), prevalence 0.25 (0.0001–0.9999), risk allele frequency for cases 0.598 and controls 0.498

#### 3.2.2 BrC

**Fig 2:**
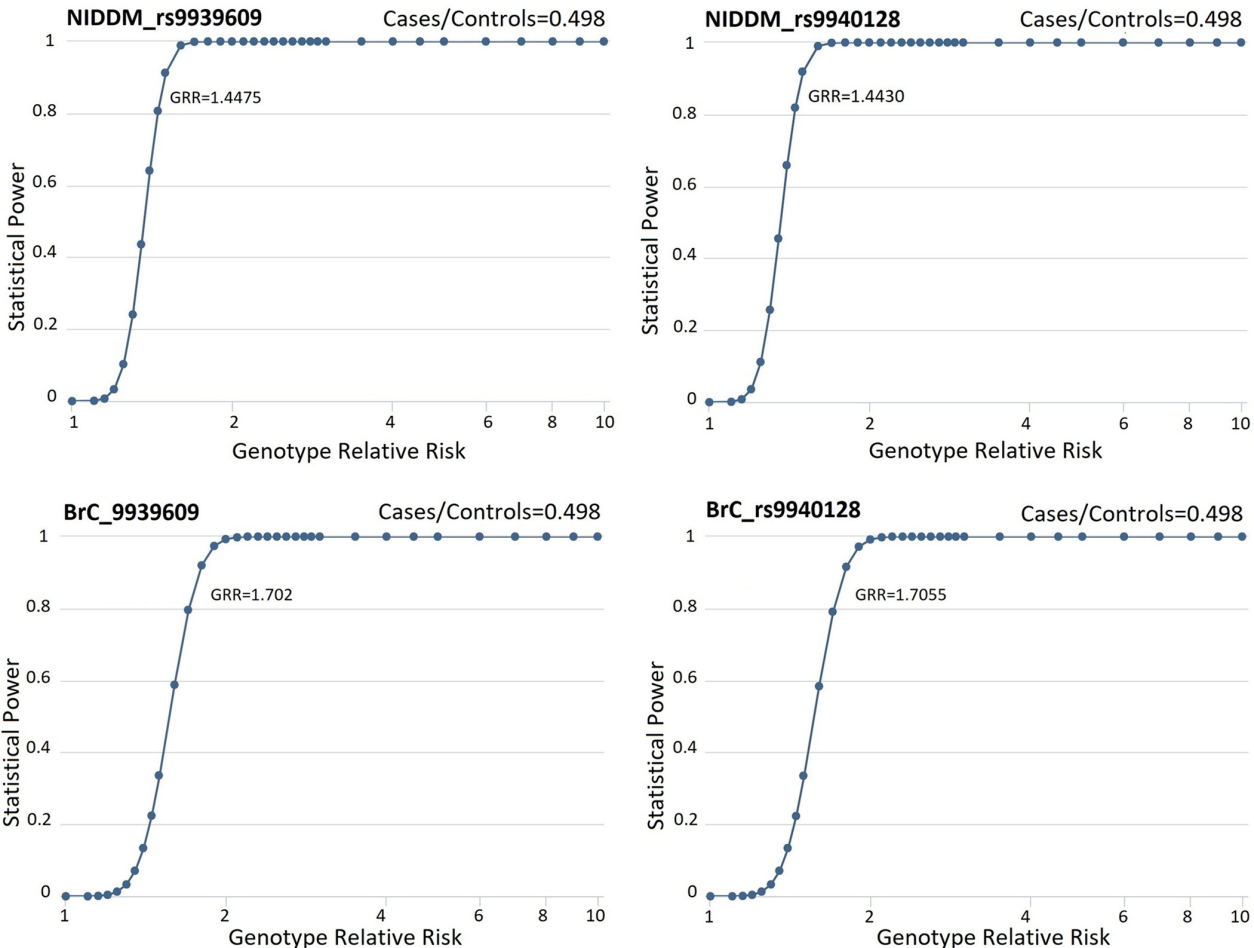
Genetic association study statistical power (GASSP) and Genotype relative risk (GRR) for NIDDM and BrC cases and controls.

One stage design of BrC 650 cases and 200 controls (case/control ratio = 0.308) for rs9939609 has a genetic association study statistical power 80% to find disease predisposing variant with GRR 1.702 (>1), prevalence 0.111 (0.0001–0.9999), risk allele frequency for cases 0.575 and controls 0.426 and 80% power to find disease predisposing variant of rs9940128 with GRR 1.7055 (>1), prevalence 0.111(0.0001–0.9999), risk allele frequency for cases 0.641 and controls 0.495 Fig 2 shows the graphical presentations of the results.

### 3.3 FTO αKG dependent dioxygenase gene rs9939609 and rs9940128 Genotyping for NIDDM

#### 3.3.1 χ^2^ Test (NIDDM)

The genotype distribution of rs9939609 was in Hardy-Weinberg Equilibrium (HWE) in the cases and controls. The genotype frequencies in NIDDM observed as wild allele homozygotes (TT) 38%, heterozygotes (AT) 47.1% and risk allele homozygotes (AA) 14.9% along with genotypes trend in control group as 35.50% TT, 49.50% AT and 15% AA. *p* value 0.93 (>0.05) for the *χ^2^* test was statistically non-significant.

The SNP rs9940128 genotypes were in HWE for NIDDM cases but controls did not follow the principle (*p* value 0.009, <0.05). Unlikely a statistically significant association with *p* value 0.007 (<0.05) for the χ^2^ test was observed for rs9940128 in NIDDM and control cases. The homozygous genotype for wild allele (GG) was 28% and 11.92%, heterozygous (GA) 48.90% and 68.22% and risk allele homozygous (AA) was 23.10% and 19.86% in NIDDM and healthy controls respectively (Table 2).

**Table 2:** Genotype frequency distributions of FTO αKG dependent dioxygenase gene SNPs rs9939609 and rs9940128 polymorphisms, expected HWE genotype frequencies for the SNPs, *χ^2^* Test *p* value along with OR (95% CI) calculations for the SNPs association with NIDDM and BrC cases versus controls.

SNP	Cases		Genotypes		Observed frequency in Cases (%)	Expected HWE frequency in Cases (%)	Observed frequency in Controls (%)	Expected HWE frequency in Controls (%)	*χ^2^* Test *p* value	OR (95% CI)	OR *p* value
rs9939609	NIDDM		AA		14.9	14.20	15	15.76	0.93**	0.99 (0.45–2.15)	1
			AT		47.10	46.59	49.50	47.96		0.90 (0.52–1.58)	0.72
			TT		38	38.20	35.50	35.76		1.11 (0.62–1.97)	0.71
			HWE		*p* value 0.99**		*p* value 0.94**				
	BrC		AA		15.05	13.21	25.81	27.20	0.00*	0.50 (0.25–1.03)	0.05
			AT		42.60	46.27	52.69	49.90		0.66 (0.38–1.16)	0.15
			TT		42.35	40.52	21.50	22.90		2.68 (1.44–4.98)	0.00
			HWE		*p* value 0.73**		*p* value 0.85**				
rs9940128	NIDDM		GG		28	27.51	11.92	21.18	0.007*	2.87 (1.36–6.05)	0.004
			GA		48.90	49.88	68.22	49.70		0.44 (0.25–0.79)	0.005
			AA		23.10	22.61	19.86	29.12		1.21 (0.61–2.38)	0.577
			HWE		*p* value 0.98**		*p* value 0.009*				
	BrC		GG		24.20	22.50	17.94	22.50	0.51**	1.45 (0.73–2.89)	0.27
			GA		52.31	49.86	58.98	49.86		0.76 (0.43–1.33)	0.34
			AA		23.49	27.64	23.08	27.64		1.02 (0.53–1.97)	1
		HWE		*p* value 0.89**			*p* value 0.18**				

^2^Statistically

*significant *p* value (<0.05) and

**non-significant (>0.05)

#### 3.3.2 OR calculations (NIDDM)

**Fig 3:**
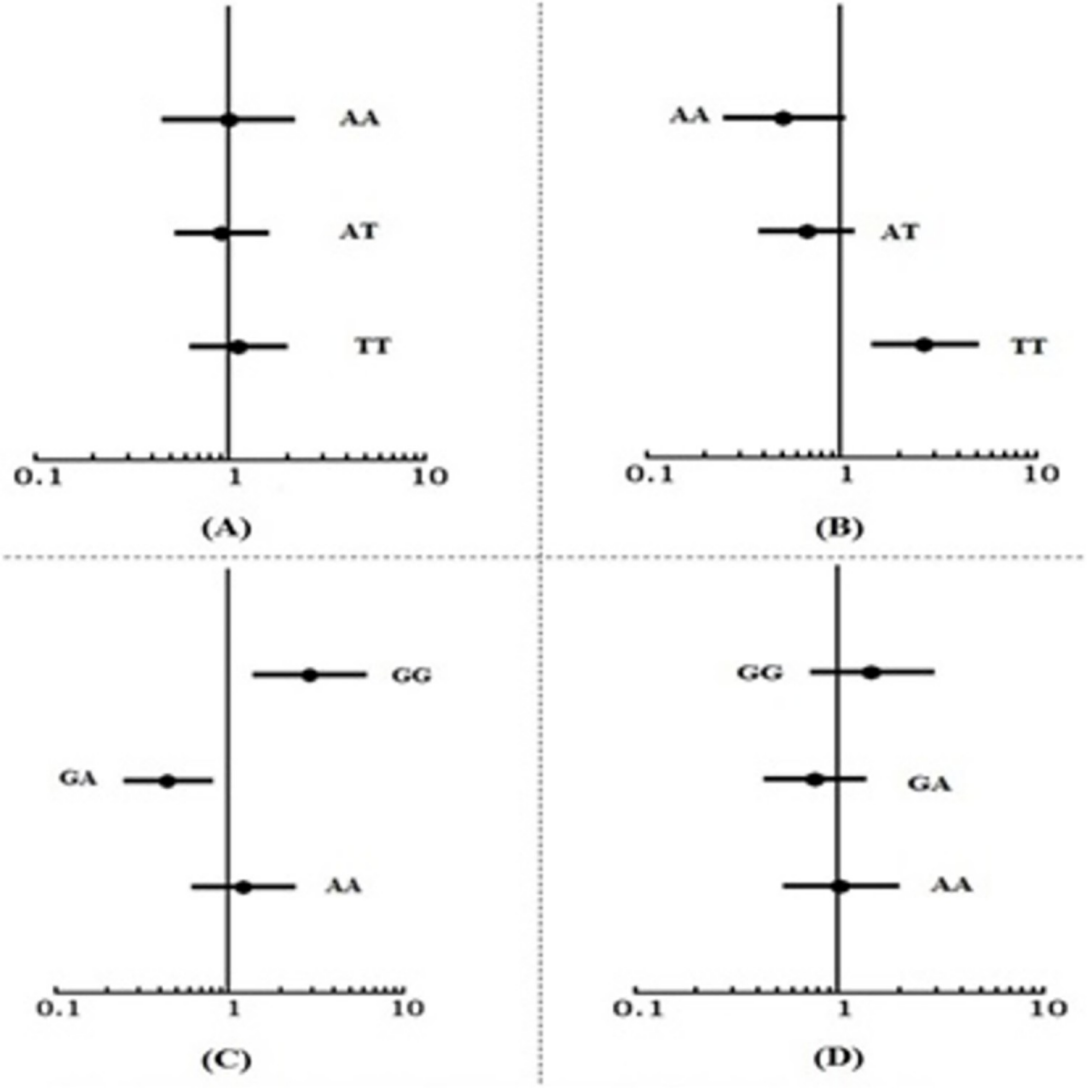
Forest Plots for genotypes of rs9939609 (A) NIDDM and (B) BrC and rs9940128 (C) NIDDM and (D) BrC.

OR with 95% CI was calculated within the NIDDM and healthy control genotypes to define to define the strength of association of both SNPs. rs9939609 OR value 0.99 (95% CI 0.45–2.15) for AA, 0.90(95% CI 0.52–1.58) for AT and 1.11 (95% CI 0.62–1.97) for TT genotypes was observed with a non-significant p value (>0.05) for all genotypes. Results are shown in Table 2 and forest plot in Fig 3(A). Whereas for rs9940128 statistically significant (p value 0.004) OR value 2.87 (95% CI 1.36–6.05) for GG, 0.44(95% CI 0.25–0.79) statistically significant (p value0.005) for GA and 1.21(95% CI 0.61–2.38) for AA non- significant (p value 0.577) were observed as shown in Table 2 and forest plots in Fig 3(C).

#### 3.3.3 Risk allele genotypes assessment (NIDDM)

The genotypes carrying the risk allele were statistically evaluated. The SNP rs9939609 results showed statistically non-significant (*p* value 0.714, >0.05) distribution in NIDDM and controls. For the similar scenario interestingly again a significant (p value 0.004, <0.05) difference was seen in the categorized genotypes in case of rs9940128 as shown in Table 3.

**Table 3:** FTO SNPs rs9939609 and rs9940128 wild and risk allele genotypes assessment for association with NIDDM and BrC.

SNP	Disease	Genotypes	Patients observed frequency, %	Control observed requency, %	p-Value for the risk assessment between patients & controls	Correlation by OR (95% CI) between patient & control	p-Value
rs9939609	NIDDM	TT	38	35.5	0.714	0.89 (0.50–1.59)	0.71
		AA/AT	62	64.5		1.11 (0.62–1.97)	0.71
	BrC	TT	42.35	21.50	0.001	2.68 (1.44–4.98)	0.001
		AA/AT	57.65	78.50		0.37 (0.20–0.69)	0.001
rs9940128	NIDDM	GG	28	11.92	0.004	2.87 (1.36–6.058)	0.004
		GA/AA	72	88.08		0.34 (0.16–0.73)	0.004
	BrC	GG	24.20	17.94	0.277	1.46 (0.73–2.89)	0.27
		GA/AA	75.80	82.06		0.68 (0.34–1.35)	0.27

### 3.4 FTO αKG dependent dioxygenase gene rs9939609 and rs9940128 Genotyping for BrC

#### 3.4.1 χ^2^ Test (BrC)

Patterns of the SNPs in case of BrC showed contrasting findings. FTO rs9939609 the genotypes distribution observed were 42.35% TT homozygotes, 42.60% AT heterozygotes and 15.05% AA homozygotes. And the prevalence of this SNP was significantly (p value 0.00, <0.05) deviating from the genotype frequencies observed in BrC healthy control group as shown in Table 2. An inadequate *p* value 0.51 (>0.05) for the statistical significance revealed null association of rs9940128 with BrC cases as compared to the controls. The genotype distribution observed in BrC and the controls was as: wild allele GG homozygote 24.20% and 17.94%, heterozygotes GA 52.31% and 58.98% and risk allele AA homozygotes 23.49% and 23.08% respectively.

#### 3.4.2 OR calculations (BrC)

FTO αKG dependent dioxygenase gene SNP rs9939609 in BrC and controls comparison based calculations statistically significant (p value = 0.05) OR value 0.50 (95% CI 0.25–1.03) for AA, 0.66 (95% CI 0.38–1.16) for AT, statistically non-significant (p value 0.15) and significant (p value 0.00) 2.68 (95% CI 1.44–4.98) for TT were observed. The forest plot for the OR results are shown in Fig 3(B). However non-significant findings for OR were revealed for rs9940128 as shown in Table 2 and forest plots in Fig 3(D).

#### 3.4.3 Risk allele genotypes assessment (BrC)

The risk allele genotypes TA/AA deciphered for rs9939609 in BrC and controls showed statistically significant variation with p value 0.001. In addition, the data for rs9940128 risk allele genotypes GA/AA did not reveal significant (*p* value 0.27) differences. The data is shown in Table 3.

## 4. Discussion

BrC and NIDDM have been on rise in Pakistan driven by drastic economic decline challenges, social norms and sedentary life style. There are frequent breast cancer and diabetes mellitus campaigns to create awareness at national level to reduce disease occurrence in Pakistan, one such campaign is mediated through telephonic ringtone voice notes. Along with the disease prevention policies, understanding of these non-communicable diseases at genetic level is needed for better clinical management through biomedical approach. FTO αKG dependent dioxygenase gene SNPs in the first intron have been found associated with adipogenesis and tumorigenesis. FTO αKG dependent dioxygenase gene (located on chromosome 16) SNPs rs9939609 and rs9940128 extend over approximately 2kb region which is present in high genetic linkage disequilibrium (LD) block (D’ 0.99 to 1). SNPs in LD block have been shown in association with BMI retrospective non communicable diseases like cancer and diabetes. The present study has been conducted to identify the involvement of the SNPs as common and or heterogeneous basis for the risk of NIDDM and BrC.

Our study strengthened the scientific body of knowledge by revealing the FTO αKG dependent dioxygenase gene variations as common basis for diabetes mellitus and breast cancer in defined pool of Pakistani population. SNPs rs9940128 and rs9939609 have been shown to independently associate with BrC and NIDDM in this population based study. SNP genotypes based discrete association of rs9940128 with NIDDM and rs9939609 with BrC in the population speculates confined disease specific involvement.

### 4.1 NIDDM

The study pool considerably showed overweight and obese cases 58.56% for NIDDM. Blood pressure and lipid profiles in NIDDM were indicative of comorbidity condition which could possibly lead to micro-vascular, macro-vascular and hepatic complications in uncontrolled hyperglycemia state as evident in the cases.

In the present study association of rs9939609 with NIDDM strikingly disapproves the consolidated disease association reports different populations. The SNP rs9939609 SNP has been regarded as future predictor or prognostic marker of NIDDM in Vietnamese population [16]. In addition, research conducted on Palestinian population reported strong association of rs9939609 with NIDDM based on statistically significant findings [17]. For NIDDM lack of rs9939609 association with or without BMI adjustment existed in North American populations, though the East Asian and European studies showed significant association [18]. There was no significant association of rs9939609 risk allele genotypes or risk allele was observed in NIDDM.

The results of SNP rs9940128 explained by the statistical attributes showed significant (*p* value 0.007, <0.05) prevalence deviation between the NIDDM cases and controls (Table 2). Likewise, the risk allele genotypes were significantly (*p* value 0.004, <0.05) higher in control group for NIDDM (Table 3). The present findings are relevant to the association of rs9940128 with development of NIDDM with respect to obesity [19].

### 4.2 BrC

The BrC overweight and obese cases were 43.13%. Menopause status in BrC can be related to life style, physiological, hormonal and genetic risk factors independently or co-dependently. Age of mother at first delivery remained independent of BrC. Tobacco and alcohol consumption and family history were not found strong determinants of NIDDM or BrC. These attributes are defined in Table 1.

The SNP rs9939609 findings in our study showed statistically significant (OR value 2.68 with 95% CI 1.44–4.98) association of the wild type allele (TT) genotypes as compared to significantly (OR value 0.50 with 95% CI 0.25–1.03) less prevalent risk allele genotypes (AA) in BrC as compared to the control group without adjusting BMI (Table 3). Contrarily a significant association of the risk allele genotype with female BrC patients has been recently discovered irrespective of anthropometric indices [5]. However, heterogeneity behavior of rs9939609 has been implicated in European, East Asian, Middle East and mixed populations when there was no association of the risk allele without BMI adjustments but risk allele was associated only with East Asian and African population after BMI adjustments [20]. In concordance to the findings on these populations where rs9939609 risk allele marginally decreased risk of BrC (OR value 0.94 with 95% CI 0.92–0.96) without adjustment for BMI, our study also highlights the plausibly lower risk of BrC with rs9939609 risk allele genotypes in Pakistani population. There was no significant association of the rs9940128 SNP as risk factor for BrC.

## 5. Conclusions

The amalgam of patients’ age, BMI and clinical hallmarks propose serious concerns for the both diseases. The unvarying association of FTO αKG dependent dioxygenase gene SNPs rs9939609 and rs9940128 are categorically genetic risk factors for BrC and NIDDM respectively in a population where the females are majorly affected. These SNPs may serve as strong prospective predictors for these diseases and useful for relocating the druggable targets.

## Supporting information

S1 File Minimal data set for breast cancer. (PDF)

S2 File Minimal data set for healthy breast cancer controls. (PDF)

S3 File Minimal data set for Type II diabetes. (PDF)

S4 File Minimal data set for Type II diabetes Controls. (PDF)

S1 Raw images (PDF)
