# Supplementary material for: Linkage disequilibrium block single-nucleotide polymorphisms in FTO alpha ketoglutarate dependent dioxygenase gene inference with breast cancer and Type II diabetes in Pakistani female population
Source: PLoS One. 2023 Jul 20;18(7):e0288934. doi: 10.1371/journal.pone.0288934 (PMC10358933; doi:10.1371/journal.pone.0288934)
Supplement: S1 File — (PDF) [file pone.0288934.s001.pdf]

S1: Minimal Data Set for Breast Cancer					
**Sample ID**	**Age in Years**	**BMI Status**	**Hypertension (mmHg)**	**Cancer Stage**	**Menopause**
BrC001	55	normal	Normal (Systolic < 120 Diastolic < 80)	2	Post menopause onset
BrC002	60	normal	Normal (Systolic < 120 Diastolic < 80)	2	Post menopause onset
BrC003	65	normal	Normal (Systolic < 120 Diastolic < 80)	3	Post menopause onset
BrC004	62	normal	Normal (Systolic < 120 Diastolic < 80)	3	Post menopause onset
BrC005	53	normal	Normal (Systolic < 120 Diastolic < 80)	1	Post menopause onset
BrC006	55	Overweight	Normal (Systolic < 120 Diastolic < 80)	2	Post menopause onset
BrC007	28	normal	Normal (Systolic < 120 Diastolic < 80)	3	Post menopause onset
BrC008	35	normal	Normal (Systolic < 120 Diastolic < 80)	3	Post menopause onset
BrC009	26	normal	Elevated (Systolic 120-129 Diastolic <80)	2	Post menopause onset
BrC010	37	normal	Normal (Systolic < 120 Diastolic < 80)	2	Post menopause onset
BrC011	53	Overweight	Normal (Systolic < 120 Diastolic < 80)	2	Post menopause onset
BrC012	40	normal	Normal (Systolic < 120 Diastolic < 80)	3	Post menopause onset
BrC013	39	normal	Normal (Systolic < 120 Diastolic < 80)	3	Post menopause onset
BrC014	60	normal	Normal (Systolic < 120 Diastolic < 80)	3	Post menopause onset
BrC015	75	normal	Normal (Systolic < 120 Diastolic < 80)	3	Post menopause onset
BrC016	61	normal	Normal (Systolic < 120 Diastolic < 80)	3	Post menopause onset
BrC017	51	Overweight	Normal (Systolic < 120 Diastolic < 80)	2	Post menopause onset
BrC018	70	normal	Normal (Systolic < 120 Diastolic < 80)	3	Post menopause onset
BrC019	24	normal	Normal (Systolic < 120 Diastolic < 80)	2	Post menopause onset
BrC020	28	normal	Normal (Systolic < 120 Diastolic < 80)	2	Post menopause onset
BrC021	60	normal	Normal (Systolic < 120 Diastolic < 80)	1	Post menopause onset
BrC022	55	normal	Normal (Systolic < 120 Diastolic < 80)	2	Post menopause onset
BrC023	70	normal	Normal (Systolic < 120 Diastolic < 80)	2	Post menopause onset
BrC024	67	normal	Elevated (Systolic 120-129 Diastolic <80)	3	Post menopause onset
BrC025	68	normal	Normal (Systolic < 120 Diastolic < 80)	2	Post menopause onset
BrC026	55	Overweight	Normal (Systolic < 120 Diastolic < 80)	3	Post menopause onset
BrC027	51	normal	Normal (Systolic < 120 Diastolic < 80)	2	Post menopause onset
BrC028	33	Overweight	Normal (Systolic < 120 Diastolic < 80)	2	Post menopause onset
BrC029	30	normal	Normal (Systolic < 120 Diastolic < 80)	2	Post menopause onset
BrC030	45	Overweight	Normal (Systolic < 120 Diastolic < 80)	3	Post menopause onset
BrC031	52	normal	Normal (Systolic < 120 Diastolic < 80)	3	Post menopause onset
BrC032	55	normal	Normal (Systolic < 120 Diastolic < 80)	1	Post menopause onset
BrC033	68	normal	Normal (Systolic < 120 Diastolic < 80)	3	Post menopause onset
BrC034	45	normal	Normal (Systolic < 120 Diastolic < 80)	2	Post menopause onset
BrC035	54	Overweight	Normal (Systolic < 120 Diastolic < 80)	2	Post menopause onset
BrC036	58	normal	Normal (Systolic < 120 Diastolic < 80)	2	Post menopause onset
BrC037	55	normal	Normal (Systolic < 120 Diastolic < 80)	2	Post menopause onset
BrC038	51	Overweight	Normal (Systolic < 120 Diastolic < 80)	2	Post menopause onset
BrC039	50	normal	Normal (Systolic < 120 Diastolic < 80)	3	Post menopause onset
BrC040	27	normal	Normal (Systolic < 120 Diastolic < 80)	3	Post menopause onset
BrC041	55	normal	Normal (Systolic < 120 Diastolic < 80)	2	Post menopause onset
BrC042	34	normal	Normal (Systolic < 120 Diastolic < 80)	3	Post menopause onset
BrC043	55	normal	Normal (Systolic < 120 Diastolic < 80)	2	Post menopause onset
BrC044	37	Obese	Normal (Systolic < 120 Diastolic < 80)	3	Post menopause onset
BrC045	51	normal	Normal (Systolic < 120 Diastolic < 80)	2	Post menopause onset
BrC046	42	Obese	Normal (Systolic < 120 Diastolic < 80)	3	Post menopause onset
BrC047	25	normal	Normal (Systolic < 120 Diastolic < 80)	2	Post menopause onset
BrC048	38	normal	Normal (Systolic < 120 Diastolic < 80)	3	Post menopause onset
BrC049	55	normal	Normal (Systolic < 120 Diastolic < 80)	2	Post menopause onset
BrC050	50	Obese	Normal (Systolic < 120 Diastolic < 80)	3	Post menopause onset
BrC051	60	normal	Normal (Systolic < 120 Diastolic < 80)	3	Post menopause onset

BrC052	29	normal	Normal (Systolic < 120 Diastolic < 80)	1	Post menopause onset
BrC053	42	normal	Normal (Systolic < 120 Diastolic < 80)	2	Post menopause onset
BrC054	27	normal	Normal (Systolic < 120 Diastolic < 80)	1	Post menopause onset
BrC055	28	Obese	Normal (Systolic < 120 Diastolic < 80)	2	Post menopause onset
BrC056	30	Obese	Normal (Systolic < 120 Diastolic < 80)	2	Post menopause onset
BrC057	31	normal	Normal (Systolic < 120 Diastolic < 80)	3	Post menopause onset
BrC058	52	Obese	Normal (Systolic < 120 Diastolic < 80)	3	Post menopause onset
BrC059	57	normal	Normal (Systolic < 120 Diastolic < 80)	2	Post menopause onset
BrC060	58	normal	Normal (Systolic < 120 Diastolic < 80)	1	Post menopause onset
BrC061	26	normal	Normal (Systolic < 120 Diastolic < 80)	2	Post menopause onset
BrC062	64	Obese	Normal (Systolic < 120 Diastolic < 80)	2	Post menopause onset
BrC063	33	normal	Normal (Systolic < 120 Diastolic < 80)	3	Post menopause onset
BrC064	65	normal	Normal (Systolic < 120 Diastolic < 80)	3	Post menopause onset
BrC065	66	Obese	Normal (Systolic < 120 Diastolic < 80)	1	Post menopause onset
BrC066	62	normal	Normal (Systolic < 120 Diastolic < 80)	2	Post menopause onset
BrC067	27	normal	Normal (Systolic < 120 Diastolic < 80)	1	Post menopause onset
BrC068	29	normal	Normal (Systolic < 120 Diastolic < 80)	2	Post menopause onset
BrC069	43	Obese	Normal (Systolic < 120 Diastolic < 80)	2	Post menopause onset
BrC070	67	normal	Normal (Systolic < 120 Diastolic < 80)	3	Post menopause onset
BrC071	37	normal	Normal (Systolic < 120 Diastolic < 80)	2	Post menopause onset
BrC072	34	Obese	Elevated (Systolic 120-129 Diastolic <80)	2	Post menopause onset
BrC073	67	normal	Normal (Systolic < 120 Diastolic < 80)	3	Post menopause onset
BrC074	70	normal	Normal (Systolic < 120 Diastolic < 80)	3	Post menopause onset
BrC075	45	normal	Normal (Systolic < 120 Diastolic < 80)	2	Post menopause onset
BrC076	70	Obese	Normal (Systolic < 120 Diastolic < 80)	3	Post menopause onset
BrC077	69	normal	Normal (Systolic < 120 Diastolic < 80)	2	Post menopause onset
BrC078	44	normal	Normal (Systolic < 120 Diastolic < 80)	2	Post menopause onset
BrC079	50	normal	Normal (Systolic < 120 Diastolic < 80)	2	Post menopause onset
BrC080	30	normal	Normal (Systolic < 120 Diastolic < 80)	2	Post menopause onset
BrC081	29	normal	Normal (Systolic < 120 Diastolic < 80)	2	Post menopause onset
BrC082	50	normal	Normal (Systolic < 120 Diastolic < 80)	3	Post menopause onset
BrC083	29	normal	Normal (Systolic < 120 Diastolic < 80)	3	Post menopause onset
BrC084	38	normal	Normal (Systolic < 120 Diastolic < 80)	3	Post menopause onset
BrC085	64	Obese	Normal (Systolic < 120 Diastolic < 80)	2	Post menopause onset
BrC086	36	normal	Normal (Systolic < 120 Diastolic < 80)	3	Post menopause onset
BrC087	28	Obese	Normal (Systolic < 120 Diastolic < 80)	3	Post menopause onset
BrC088	43	normal	Normal (Systolic < 120 Diastolic < 80)	2	Post menopause onset
BrC089	50	normal	Normal (Systolic < 120 Diastolic < 80)	2	Post menopause onset
BrC090	68	normal	Normal (Systolic < 120 Diastolic < 80)	3	Post menopause onset
BrC091	69	normal	Normal (Systolic < 120 Diastolic < 80)	2	Post menopause onset
BrC092	61	Obese	Normal (Systolic < 120 Diastolic < 80)	2	Post menopause onset
BrC093	29	normal	Normal (Systolic < 120 Diastolic < 80)	2	Post menopause onset
BrC094	37	normal	Normal (Systolic < 120 Diastolic < 80)	2	Post menopause onset
BrC095	39	normal	Normal (Systolic < 120 Diastolic < 80)	2	Post menopause onset
BrC096	46	normal	Normal (Systolic < 120 Diastolic < 80)	2	Post menopause onset
BrC097	37	Obese	Normal (Systolic < 120 Diastolic < 80)	2	Post menopause onset
BrC098	31	normal	Normal (Systolic < 120 Diastolic < 80)	2	Post menopause onset
BrC099	27	normal	Normal (Systolic < 120 Diastolic < 80)	2	Post menopause onset
BrC100	26	normal	Elevated (Systolic 120-129 Diastolic <80)	1	Post menopause onset
BrC101	36	normal	Normal (Systolic < 120 Diastolic < 80)	2	Post menopause onset
BrC102	27	Obese	Normal (Systolic < 120 Diastolic < 80)	2	Post menopause onset
BrC103	30	normal	Normal (Systolic < 120 Diastolic < 80)	2	Post menopause onset
BrC104	53	normal	Normal (Systolic < 120 Diastolic < 80)	2	Post menopause onset

BrC105	24	normal	Normal (Systolic < 120 Diastolic < 80)	1	Post menopause onset
BrC106	40	normal	Normal (Systolic < 120 Diastolic < 80)	2	Post menopause onset
BrC107	71	Obese	Normal (Systolic < 120 Diastolic < 80)	1	Post menopause onset
BrC108	57	Obese	Normal (Systolic < 120 Diastolic < 80)	1	Post menopause onset
BrC109	59	normal	Normal (Systolic < 120 Diastolic < 80)	2	Post menopause onset
BrC110	51	normal	Normal (Systolic < 120 Diastolic < 80)	1	Post menopause onset
BrC111	37	normal	Normal (Systolic < 120 Diastolic < 80)	2	Post menopause onset
BrC112	56	normal	Normal (Systolic < 120 Diastolic < 80)	2	Post menopause onset
BrC113	63	normal	Normal (Systolic < 120 Diastolic < 80)	1	Post menopause onset
BrC114	36	normal	Normal (Systolic < 120 Diastolic < 80)	1	Post menopause onset
BrC115	36	normal	Normal (Systolic < 120 Diastolic < 80)	2	Post menopause onset
BrC116	60	normal	Normal (Systolic < 120 Diastolic < 80)	2	Post menopause onset
BrC117	26	Obese	Normal (Systolic < 120 Diastolic < 80)	1	Post menopause onset
BrC118	43	normal	Normal (Systolic < 120 Diastolic < 80)	2	Post menopause onset
BrC119	36	normal	Normal (Systolic < 120 Diastolic < 80)	2	Post menopause onset
BrC120	27	normal	Normal (Systolic < 120 Diastolic < 80)	2	Post menopause onset
BrC121	73	normal	Normal (Systolic < 120 Diastolic < 80)	3	Post menopause onset
BrC122	51	normal	Normal (Systolic < 120 Diastolic < 80)	2	Post menopause onset
BrC123	36	Obese	Normal (Systolic < 120 Diastolic < 80)	2	Post menopause onset
BrC124	38	Obese	Normal (Systolic < 120 Diastolic < 80)	2	Post menopause onset
BrC125	55	normal	Normal (Systolic < 120 Diastolic < 80)	2	Post menopause onset
BrC126	40	normal	Normal (Systolic < 120 Diastolic < 80)	2	Post menopause onset
BrC127	30	normal	Normal (Systolic < 120 Diastolic < 80)	1	Post menopause onset
BrC128	40	normal	Normal (Systolic < 120 Diastolic < 80)	1	Post menopause onset
BrC129	25	Obese	Normal (Systolic < 120 Diastolic < 80)	2	Post menopause onset
BrC130	65	normal	Normal (Systolic < 120 Diastolic < 80)	3	Post menopause onset
BrC131	29	normal	Normal (Systolic < 120 Diastolic < 80)	2	Post menopause onset
BrC132	33	Obese	Elevated (Systolic 120-129 Diastolic <80)	3	Post menopause onset
BrC133	25	normal	Normal (Systolic < 120 Diastolic < 80)	3	Post menopause onset
BrC134	56	Obese	Normal (Systolic < 120 Diastolic < 80)	2	Post menopause onset
BrC135	33	normal	Normal (Systolic < 120 Diastolic < 80)	2	Post menopause onset
BrC136	51	normal	Normal (Systolic < 120 Diastolic < 80)	3	Post menopause onset
BrC137	70	Obese	Normal (Systolic < 120 Diastolic < 80)	3	Post menopause onset
BrC138	37	Obese	Normal (Systolic < 120 Diastolic < 80)	3	Post menopause onset
BrC139	56	normal	Normal (Systolic < 120 Diastolic < 80)	2	Post menopause onset
BrC140	57	Obese	Normal (Systolic < 120 Diastolic < 80)	3	Post menopause onset
BrC141	72	normal	Normal (Systolic < 120 Diastolic < 80)	3	Post menopause onset
BrC142	54	normal	Normal (Systolic < 120 Diastolic < 80)	2	Post menopause onset
BrC143	41	normal	Normal (Systolic < 120 Diastolic < 80)	2	Post menopause onset
BrC144	48	Obese	Normal (Systolic < 120 Diastolic < 80)	2	Post menopause onset
BrC145	55	normal	Normal (Systolic < 120 Diastolic < 80)	3	Post menopause onset
BrC146	67	Obese	Normal (Systolic < 120 Diastolic < 80)	2	Post menopause onset
BrC147	58	normal	Normal (Systolic < 120 Diastolic < 80)	3	Post menopause onset
BrC148	33	Obese	Normal (Systolic < 120 Diastolic < 80)	2	Post menopause onset
BrC149	50	normal	Normal (Systolic < 120 Diastolic < 80)	2	Post menopause onset
BrC150	67	normal	Normal (Systolic < 120 Diastolic < 80)	2	Post menopause onset
BrC151	68	Obese	Normal (Systolic < 120 Diastolic < 80)	2	Post menopause onset
BrC152	33	normal	Normal (Systolic < 120 Diastolic < 80)	2	Post menopause onset
BrC153	40	normal	Normal (Systolic < 120 Diastolic < 80)	1	Post menopause onset
BrC154	38	Obese	Normal (Systolic < 120 Diastolic < 80)	3	Post menopause onset
BrC155	50	Obese	Normal (Systolic < 120 Diastolic < 80)	3	Post menopause onset
BrC156	64	normal	Normal (Systolic < 120 Diastolic < 80)	2	Post menopause onset
BrC157	61	Obese	Normal (Systolic < 120 Diastolic < 80)	3	Post menopause onset

BrC158	66	normal	Normal (Systolic < 120 Diastolic < 80)	3	Post menopause onset
BrC159	63	Obese	Normal (Systolic < 120 Diastolic < 80)	2	Post menopause onset
BrC160	35	normal	Normal (Systolic < 120 Diastolic < 80)	3	Post menopause onset
BrC161	53	normal	Normal (Systolic < 120 Diastolic < 80)	3	Post menopause onset
BrC162	51	Obese	Normal (Systolic < 120 Diastolic < 80)	3	Post menopause onset
BrC163	45	Obese	Normal (Systolic < 120 Diastolic < 80)	2	Post menopause onset
BrC164	53	Obese	Normal (Systolic < 120 Diastolic < 80)	2	Post menopause onset
BrC165	51	normal	Normal (Systolic < 120 Diastolic < 80)	2	Post menopause onset
BrC166	70	Obese	Normal (Systolic < 120 Diastolic < 80)	3	Post menopause onset
BrC167	51	normal	Normal (Systolic < 120 Diastolic < 80)	2	Post menopause onset
BrC168	36	normal	Normal (Systolic < 120 Diastolic < 80)	2	Post menopause onset
BrC169	74	Obese	Normal (Systolic < 120 Diastolic < 80)	2	Post menopause onset
BrC170	31	normal	Normal (Systolic < 120 Diastolic < 80)	3	Post menopause onset
BrC171	60	normal	Normal (Systolic < 120 Diastolic < 80)	1	Post menopause onset
BrC172	69	normal	Normal (Systolic < 120 Diastolic < 80)	3	Post menopause onset
BrC173	27	Obese	Normal (Systolic < 120 Diastolic < 80)	3	Post menopause onset
BrC174	42	normal	Elevated (Systolic 120-129 Diastolic <80)	2	Post menopause onset
BrC175	68	normal	Normal (Systolic < 120 Diastolic < 80)	0	Pre menopause onset
BrC176	56	normal	Normal (Systolic < 120 Diastolic < 80)	0	Post menopause onset
BrC177	67	Obese	Normal (Systolic < 120 Diastolic < 80)	0	Post menopause onset
BrC178	40	normal	Normal (Systolic < 120 Diastolic < 80)	2	Post menopause onset
BrC179	69	obese	Normal (Systolic < 120 Diastolic < 80)	0	Post menopause onset
BrC180	36	obese	Normal (Systolic < 120 Diastolic < 80)	0	Premenopause onset
BrC181	43	normal	Normal (Systolic < 120 Diastolic < 80)	2	Post menopause onset
BrC182	70	normal	Normal (Systolic < 120 Diastolic < 80)	3	Post menopause onset
BrC183	32	normal	Normal (Systolic < 120 Diastolic < 80)	0	Post menopause onset
BrC184	61	Normal	Normal (Systolic < 120 Diastolic < 80)	0	Post menopause onset
BrC185	57	obese	Normal (Systolic < 120 Diastolic < 80)	2	Post menopause onset
BrC186	61	normal	Normal (Systolic < 120 Diastolic < 80)	3	Post menopause onset
BrC187	40	normal	Normal (Systolic < 120 Diastolic < 80)	3	Post menopause onset
BrC188	64	obese	Elevated (Systolic 120-129 Diastolic <80)	2	Premenopause onset
BrC189	42	normal	Normal (Systolic < 120 Diastolic < 80)	0	Post menopause onset
BrC190	67	normal	Normal (Systolic < 120 Diastolic < 80)	1	Post menopause onset
BrC191	42	normal	Normal (Systolic < 120 Diastolic < 80)	2	Premenopause onset
BrC192	69	normal	Normal (Systolic < 120 Diastolic < 80)	2	Post menopause onset
BrC193	40	normal	Normal (Systolic < 120 Diastolic < 80)	0	Post menopause onset
BrC194	44	obese	Normal (Systolic < 120 Diastolic < 80)	3	Premenopause onset
BrC195	33	normal	Normal (Systolic < 120 Diastolic < 80)	0	Post menopause onset
BrC196	50	normal	Normal (Systolic < 120 Diastolic < 80)	3	Post menopause onset
BrC197	65	normal	Normal (Systolic < 120 Diastolic < 80)	3	Post menopause onset
BrC198	59	normal	Normal (Systolic < 120 Diastolic < 80)	0	Post menopause onset
BrC199	63	obese	Normal (Systolic < 120 Diastolic < 80)	0	Pre menopause onset
BrC200	39	normal	Normal (Systolic < 120 Diastolic < 80)	2	Post menopause onset
BrC201	40	normal	Normal (Systolic < 120 Diastolic < 80)	2	Post menopause onset
BrC202	68	normal	Normal (Systolic < 120 Diastolic < 80)	0	Pre menopause onset
BrC203	38	normal	Normal (Systolic < 120 Diastolic < 80)	0	Pre menopause onset
BrC204	35	Obese	Normal (Systolic < 120 Diastolic < 80)	2	Pre menopause onset
BrC205	60	normal	Normal (Systolic < 120 Diastolic < 80)	0	Post menopause onset
BrC206	58	normal	Normal (Systolic < 120 Diastolic < 80)	0	Post menopause onset
BrC207	62	obese	Normal (Systolic < 120 Diastolic < 80)	0	Post menopause onset
BrC208	55	normal	Normal (Systolic < 120 Diastolic < 80)	0	Post menopause onset
BrC209	70	obese	Normal (Systolic < 120 Diastolic < 80)	1	Post menopause onset
BrC210	65	normal	Normal (Systolic < 120 Diastolic < 80)	3	Post menopause onset

BrC211	39	obese	Normal (Systolic < 120 Diastolic < 80)	3	Pre menopause onset
BrC212	26	Normal	Normal (Systolic < 120 Diastolic < 80)	0	Pre menopause onset
BrC213	54	normal	Normal (Systolic < 120 Diastolic < 80)	0	Post menopause onset
BrC214	29	normal	Normal (Systolic < 120 Diastolic < 80)	2	Pre menopause onset
BrC215	62	obese	Normal (Systolic < 120 Diastolic < 80)	0	Pre menopause onset
BrC216	40	obese	Normal (Systolic < 120 Diastolic < 80)	3	Pre menopause onset
BrC217	38	normal	Normal (Systolic < 120 Diastolic < 80)	0	Pre menopause onset
BrC218	36	normal	Normal (Systolic < 120 Diastolic < 80)	3	Pre menopause onset
BrC219	30	obese	Normal (Systolic < 120 Diastolic < 80)	0	Post menopause onset
BrC220	60	normal	Normal (Systolic < 120 Diastolic < 80)	3	Post menopause onset
BrC221	70	normal	Normal (Systolic < 120 Diastolic < 80)	2	Pre menopause onset
BrC222	38	normal	Normal (Systolic < 120 Diastolic < 80)	0	Post menopause onset
BrC223	67	normal	Normal (Systolic < 120 Diastolic < 80)	0	Pre menopause onset
BrC224	71	normal	Normal (Systolic < 120 Diastolic < 80)	0	Post menopause onset
BrC225	31	obese	Normal (Systolic < 120 Diastolic < 80)	1	Pre menopause onset
BrC226	71	obese	Normal (Systolic < 120 Diastolic < 80)	3	Pre menopause onset
BrC227	68	normal	Normal (Systolic < 120 Diastolic < 80)	1	Post menopause onset
BrC228	38	normal	Normal (Systolic < 120 Diastolic < 80)	0	Post menopause onset
BrC229	65	obese	Normal (Systolic < 120 Diastolic < 80)	0	Pre menopause onset
BrC230	35	normal	Normal (Systolic < 120 Diastolic < 80)	0	Post menopause onset
BrC231	46	normal	Normal (Systolic < 120 Diastolic < 80)	4	Pre menopause onset
BrC232	37	normal	Normal (Systolic < 120 Diastolic < 80)	3	Pre menopause onset
BrC233	41	normal	Normal (Systolic < 120 Diastolic < 80)	2	Post menopause onset
BrC234	57	normal	Normal (Systolic < 120 Diastolic < 80)	0	Post menopause onset
BrC235	54	normal	Normal (Systolic < 120 Diastolic < 80)	2	Post menopause onset
BrC236	66	obese	Normal (Systolic < 120 Diastolic < 80)	2	Post menopause onset
BrC237	58	normal	Normal (Systolic < 120 Diastolic < 80)	2	Post menopause onset
BrC238	40	normal	Normal (Systolic < 120 Diastolic < 80)	0	Post menopause onset
BrC239	63	normal	Normal (Systolic < 120 Diastolic < 80)	1	Post menopause onset
BrC240	71	normal	Normal (Systolic < 120 Diastolic < 80)	0	Post menopause onset
BrC241	53	normal	Normal (Systolic < 120 Diastolic < 80)	0	Post menopause onset
BrC242	30	normal	Normal (Systolic < 120 Diastolic < 80)	0	Pre menopause onset
BrC243	44	obese	Normal (Systolic < 120 Diastolic < 80)	0	Post menopause onset
BrC244	42	normal	Normal (Systolic < 120 Diastolic < 80)	3	Post menopause onset
BrC245	67	normal	Normal (Systolic < 120 Diastolic < 80)	2	Post menopause onset
BrC246	55	normal	Normal (Systolic < 120 Diastolic < 80)	0	Post menopause onset
BrC247	72	normal	Normal (Systolic < 120 Diastolic < 80)	3	Post menopause onset
BrC248	34	normal	Elevated (Systolic 120-129 Diastolic <80)	0	Post menopause onset
BrC249	45	obese	Normal (Systolic < 120 Diastolic < 80)	2	Post menopause onset
BrC250	60	normal	Normal (Systolic < 120 Diastolic < 80)	0	Premenopause onset
BrC251	68	normal	Normal (Systolic < 120 Diastolic < 80)	0	Post menopause onset
BrC252	53	normal	Normal (Systolic < 120 Diastolic < 80)	0	Post menopause onset
BrC253	42	normal	Normal (Systolic < 120 Diastolic < 80)	2	Pre menopause onset
BrC254	42	normal	Normal (Systolic < 120 Diastolic < 80)	1	Premenopause onset
BrC255	35	normal	Normal (Systolic < 120 Diastolic < 80)	2	Post menopause onset
BrC256	42	normal	Normal (Systolic < 120 Diastolic < 80)	0	Post menopause onset
BrC257	70	normal	Normal (Systolic < 120 Diastolic < 80)	3	Pre menopause onset
BrC258	39	normal	Normal (Systolic < 120 Diastolic < 80)	2	Post menopause onset
BrC259	71	normal	Normal (Systolic < 120 Diastolic < 80)	0	Post menopause onset
BrC260	30	normal	Normal (Systolic < 120 Diastolic < 80)	4	Pre menopause onset
BrC261	20	normal	Normal (Systolic < 120 Diastolic < 80)	0	Post menopause onset
BrC262	35	normal	Normal (Systolic < 120 Diastolic < 80)	2	Post menopause onset
BrC263	23	obese	Normal (Systolic < 120 Diastolic < 80)	3	Post menopause onset

BrC264	35	normal	Normal (Systolic < 120 Diastolic < 80)	2	Post menopause onset
BrC265	40	obese	Normal (Systolic < 120 Diastolic < 80)	0	Post menopause onset
BrC266	35	obese	Normal (Systolic < 120 Diastolic < 80)	1	Post menopause onset
BrC267	33	normal	Normal (Systolic < 120 Diastolic < 80)	0	Post menopause onset
BrC268	71	normal	Normal (Systolic < 120 Diastolic < 80)	0	Post menopause onset
BrC269	35	obese	Normal (Systolic < 120 Diastolic < 80)	2	Post menopause onset
BrC270	65	obese	Normal (Systolic < 120 Diastolic < 80)	2	Post menopause onset
BrC271	35	obese	Normal (Systolic < 120 Diastolic < 80)	3	Post menopause onset
BrC272	32	normal	Normal (Systolic < 120 Diastolic < 80)	0	Post menopause onset
BrC273	31	obese	Normal (Systolic < 120 Diastolic < 80)	3	Post menopause onset
BrC274	34	normal	Elevated (Systolic 120-129 Diastolic <80)	2	Post menopause onset
BrC275	65	normal	Normal (Systolic < 120 Diastolic < 80)	1	Post menopause onset
BrC276	68	normal	Normal (Systolic < 120 Diastolic < 80)	4	Post menopause onset
BrC277	25	normal	Normal (Systolic < 120 Diastolic < 80)	2	Pre menopause onset
BrC278	52	obese	Normal (Systolic < 120 Diastolic < 80)	2	Pre menopause onset
BrC279	35	obese	Normal (Systolic < 120 Diastolic < 80)	2	Pre menopause onset
BrC280	28	normal	Normal (Systolic < 120 Diastolic < 80)	0	Pre menopause onset
BrC281	26	normal	Normal (Systolic < 120 Diastolic < 80)	3	Pre menopause onset
BrC282	71	obese	Normal (Systolic < 120 Diastolic < 80)	4	Pre menopause onset
BrC283	33	normal	Normal (Systolic < 120 Diastolic < 80)	3	Pre menopause onset
BrC284	65	normal	Normal (Systolic < 120 Diastolic < 80)	2	Pre menopause onset
BrC285	34	normal	Normal (Systolic < 120 Diastolic < 80)	3	Pre menopause onset
BrC286	69	normal	Normal (Systolic < 120 Diastolic < 80)	2	Pre menopause onset
BrC287	33	obese	Normal (Systolic < 120 Diastolic < 80)	0	Pre menopause onset
BrC288	62	normal	Normal (Systolic < 120 Diastolic < 80)	2	Pre menopause onset
BrC289	69	normal	Normal (Systolic < 120 Diastolic < 80)	3	Pre menopause onset
BrC290	60	obese	Normal (Systolic < 120 Diastolic < 80)	2	Pre menopause onset
BrC291	55	normal	Normal (Systolic < 120 Diastolic < 80)	4	Pre menopause onset
BrC292	27	normal	Normal (Systolic < 120 Diastolic < 80)	3	Pre menopause onset
BrC293	35	normal	Normal (Systolic < 120 Diastolic < 80)	1	Pre menopause onset
BrC294	65	normal	Normal (Systolic < 120 Diastolic < 80)	2	Pre menopause onset
BrC295	23	Overweight	Normal (Systolic < 120 Diastolic < 80)	2	Pre menopause onset
BrC296	65	normal	Normal (Systolic < 120 Diastolic < 80)	2	Pre menopause onset
BrC297	62	normal	Normal (Systolic < 120 Diastolic < 80)	3	Post menopause onset
BrC298	39	obese	Normal (Systolic < 120 Diastolic < 80)	0	Post menopause onset
BrC299	66	normal	Normal (Systolic < 120 Diastolic < 80)	0	Post menopause onset
BrC300	71	normal	Normal (Systolic < 120 Diastolic < 80)	0	Post menopause onset
BrC301	29	normal	Normal (Systolic < 120 Diastolic < 80)	0	Post menopause onset
BrC302	25	normal	Normal (Systolic < 120 Diastolic < 80)	0	Post menopause onset
BrC303	70	normal	Normal (Systolic < 120 Diastolic < 80)	0	Post menopause onset
BrC304	35	obese	Normal (Systolic < 120 Diastolic < 80)	3	Post menopause onset
BrC305	49	normal	Normal (Systolic < 120 Diastolic < 80)	0	Post menopause onset
BrC306	20	obese	Normal (Systolic < 120 Diastolic < 80)	2	Post menopause onset
BrC307	70	obese	Normal (Systolic < 120 Diastolic < 80)	2	Pre menopause onset
BrC308	35	normal	Normal (Systolic < 120 Diastolic < 80)	2	Pre menopause onset
BrC309	40	normal	Normal (Systolic < 120 Diastolic < 80)	2	Pre menopause onset
BrC310	63	obese	Normal (Systolic < 120 Diastolic < 80)	3	Pre menopause onset
BrC311	63	obese	Normal (Systolic < 120 Diastolic < 80)	0	Pre menopause onset
BrC312	67	obese	Normal (Systolic < 120 Diastolic < 80)	0	Pre menopause onset
BrC313	40	obese	Normal (Systolic < 120 Diastolic < 80)	0	Pre menopause onset
BrC314	20	obese	Normal (Systolic < 120 Diastolic < 80)	0	Pre menopause onset
BrC315	35	Overweight	Normal (Systolic < 120 Diastolic < 80)	4	Pre menopause onset
BrC316	70	obese	Normal (Systolic < 120 Diastolic < 80)	0	Pre menopause onset

BrC317	30	normal	Normal (Systolic < 120 Diastolic < 80)	2	Pre menopause onset
BrC318	33	obese	Normal (Systolic < 120 Diastolic < 80)	3	Pre menopause onset
BrC319	37	obese	Normal (Systolic < 120 Diastolic < 80)	2	Pre menopause onset
BrC320	37	obese	Normal (Systolic < 120 Diastolic < 80)	0	Pre menopause onset
BrC321	65	normal	Normal (Systolic < 120 Diastolic < 80)	3	Pre menopause onset
BrC322	70	obese	Normal (Systolic < 120 Diastolic < 80)	2	Pre menopause onset
BrC323	60	normal	Normal (Systolic < 120 Diastolic < 80)	2	Pre menopause onset
BrC324	35	normal	Normal (Systolic < 120 Diastolic < 80)	0	Pre menopause onset
BrC325	60	normal	Normal (Systolic < 120 Diastolic < 80)	0	Pre menopause onset
BrC326	69	normal	Normal (Systolic < 120 Diastolic < 80)	1	Pre menopause onset
BrC327	55	normal	Normal (Systolic < 120 Diastolic < 80)	2	Pre menopause onset
BrC328	50	obese	Normal (Systolic < 120 Diastolic < 80)	4	Pre menopause onset
BrC329	71	normal	Normal (Systolic < 120 Diastolic < 80)	0	Pre menopause onset
BrC330	22	normal	Normal (Systolic < 120 Diastolic < 80)	2	Pre menopause onset
BrC331	27	obese	Normal (Systolic < 120 Diastolic < 80)	0	Pre menopause onset
BrC332	57	normal	Normal (Systolic < 120 Diastolic < 80)	0	Pre menopause onset
BrC333	31	obese	Normal (Systolic < 120 Diastolic < 80)	3	Pre menopause onset
BrC334	22	normal	Normal (Systolic < 120 Diastolic < 80)	0	Pre menopause onset
BrC335	35	obese	Normal (Systolic < 120 Diastolic < 80)	2	Pre menopause onset
BrC336	32	normal	Normal (Systolic < 120 Diastolic < 80)	0	Post menopause onset
BrC337	25	obese	Normal (Systolic < 120 Diastolic < 80)	3	Post menopause onset
BrC338	50	normal	Normal (Systolic < 120 Diastolic < 80)	3	Post menopause onset
BrC339	61	normal	Normal (Systolic < 120 Diastolic < 80)	0	Post menopause onset
BrC340	40	Overweight	Normal (Systolic < 120 Diastolic < 80)	0	Post menopause onset
BrC341	62	normal	Normal (Systolic < 120 Diastolic < 80)	0	Post menopause onset
BrC342	22	obese	Normal (Systolic < 120 Diastolic < 80)	2	Post menopause onset
BrC343	40	obese	Normal (Systolic < 120 Diastolic < 80)	2	Pre menopause onset
BrC344	60	normal	Normal (Systolic < 120 Diastolic < 80)	0	Pre menopause onset
BrC345	63	normal	Normal (Systolic < 120 Diastolic < 80)	0	Pre menopause onset
BrC346	41	Overweight	Normal (Systolic < 120 Diastolic < 80)	3	Pre menopause onset
BrC347	35	obese	Normal (Systolic < 120 Diastolic < 80)	0	Pre menopause onset
BrC348	38	normal	Normal (Systolic < 120 Diastolic < 80)	2	Pre menopause onset
BrC349	60	obese	Elevated (Systolic 120-129 Diastolic <80)	0	Pre menopause onset
BrC350	71	normal	Normal (Systolic < 120 Diastolic < 80)	0	Pre menopause onset
BrC351	26	normal	Normal (Systolic < 120 Diastolic < 80)	0	Pre menopause onset
BrC352	65	obese	Normal (Systolic < 120 Diastolic < 80)	0	Pre menopause onset
BrC353	35	obese	Normal (Systolic < 120 Diastolic < 80)	0	Pre menopause onset
BrC354	30	obese	Normal (Systolic < 120 Diastolic < 80)	0	Pre menopause onset
BrC355	35	obese	Normal (Systolic < 120 Diastolic < 80)	0	Pre menopause onset
BrC356	28	normal	Normal (Systolic < 120 Diastolic < 80)	0	Pre menopause onset
BrC357	65	obese	Normal (Systolic < 120 Diastolic < 80)	0	Pre menopause onset
BrC358	37	Overweight	Normal (Systolic < 120 Diastolic < 80)	0	Pre menopause onset
BrC359	35	obese	Normal (Systolic < 120 Diastolic < 80)	0	Pre menopause onset
BrC360	28	normal	Normal (Systolic < 120 Diastolic < 80)	4	Pre menopause onset
BrC361	26	Overweight	Normal (Systolic < 120 Diastolic < 80)	0	Pre menopause onset
BrC362	34	normal	Normal (Systolic < 120 Diastolic < 80)	0	Post menopause onset
BrC363	63	normal	Normal (Systolic < 120 Diastolic < 80)	0	Post menopause onset
BrC364	60	obese	Normal (Systolic < 120 Diastolic < 80)	0	Post menopause onset
BrC365	65	obese	Normal (Systolic < 120 Diastolic < 80)	0	Pre menopause onset
BrC366	27	obese	Normal (Systolic < 120 Diastolic < 80)	0	Pre menopause onset
BrC367	40	obese	Normal (Systolic < 120 Diastolic < 80)	0	Pre menopause onset
BrC368	25	normal	Normal (Systolic < 120 Diastolic < 80)	2	Pre menopause onset
BrC369	33	normal	Normal (Systolic < 120 Diastolic < 80)	0	Pre menopause onset

BrC370	32	Overweight	Normal (Systolic < 120 Diastolic < 80)	0	Pre menopause onset
BrC371	27	normal	Normal (Systolic < 120 Diastolic < 80)	4	Post menopause onset
BrC372	36	normal	Normal (Systolic < 120 Diastolic < 80)	0	Post menopause onset
BrC373	35	obese	Normal (Systolic < 120 Diastolic < 80)	3	Post menopause onset
BrC374	64	normal	Normal (Systolic < 120 Diastolic < 80)	0	Post menopause onset
BrC375	33	normal	Normal (Systolic < 120 Diastolic < 80)	0	Post menopause onset
BrC376	40	obese	Normal (Systolic < 120 Diastolic < 80)	0	Post menopause onset
BrC377	50	obese	Normal (Systolic < 120 Diastolic < 80)	0	Post menopause onset
BrC378	55	Overweight	Normal (Systolic < 120 Diastolic < 80)	0	Pre menopause onset
BrC379	24	Overweight	Normal (Systolic < 120 Diastolic < 80)	0	Pre menopause onset
BrC380	65	obese	Normal (Systolic < 120 Diastolic < 80)	0	Pre menopause onset
BrC381	65	obese	Normal (Systolic < 120 Diastolic < 80)	2	Pre menopause onset
BrC382	35	obese	Normal (Systolic < 120 Diastolic < 80)	0	Pre menopause onset
BrC383	60	normal	Normal (Systolic < 120 Diastolic < 80)	0	Pre menopause onset
BrC384	35	normal	Normal (Systolic < 120 Diastolic < 80)	0	Premenopause onset
BrC385	72	obese	Normal (Systolic < 120 Diastolic < 80)	0	Post menopause onset
BrC386	36	normal	Normal (Systolic < 120 Diastolic < 80)	0	Post menopause onset
BrC387	67	obese	Normal (Systolic < 120 Diastolic < 80)	0	Post menopause onset
BrC388	34	normal	Normal (Systolic < 120 Diastolic < 80)	0	Post menopause onset
BrC389	60	normal	Normal (Systolic < 120 Diastolic < 80)	3	Post menopause onset
BrC390	27	normal	Normal (Systolic < 120 Diastolic < 80)	0	Post menopause onset
BrC391	45	obese	Normal (Systolic < 120 Diastolic < 80)	0	Post menopause onset
BrC392	60	obese	Normal (Systolic < 120 Diastolic < 80)	4	Post menopause onset
BrC393	65	normal	Normal (Systolic < 120 Diastolic < 80)	0	Post menopause onset
BrC394	40	obese	Normal (Systolic < 120 Diastolic < 80)	0	Post menopause onset
BrC395	60	normal	Normal (Systolic < 120 Diastolic < 80)	0	Post menopause onset
BrC396	67	obese	Normal (Systolic < 120 Diastolic < 80)	3	Post menopause onset
BrC397	37	obese	Normal (Systolic < 120 Diastolic < 80)	0	Post menopause onset
BrC398	65	normal	Normal (Systolic < 120 Diastolic < 80)	0	Post menopause onset
BrC399	25	normal	Elevated (Systolic 120-129 Diastolic <80)	0	Post menopause onset
BrC400	71	normal	Normal (Systolic < 120 Diastolic < 80)	0	Premenopause onset
BrC401	65	normal	Normal (Systolic < 120 Diastolic < 80)	3	Premenopause onset
BrC402	40	normal	Normal (Systolic < 120 Diastolic < 80)	0	Premenopause onset
BrC403	24	obese	Normal (Systolic < 120 Diastolic < 80)	0	Post menopause onset
BrC404	36	normal	Normal (Systolic < 120 Diastolic < 80)	4	Post menopause onset
BrC405	27	normal	Normal (Systolic < 120 Diastolic < 80)	0	Post menopause onset
BrC406	35	Overweight	Normal (Systolic < 120 Diastolic < 80)	0	Post menopause onset
BrC407	61	obese	Normal (Systolic < 120 Diastolic < 80)	0	Post menopause onset
BrC408	28	normal	Normal (Systolic < 120 Diastolic < 80)	0	Post menopause onset
BrC409	31	obese	Normal (Systolic < 120 Diastolic < 80)	4	Post menopause onset
BrC410	60	normal	Normal (Systolic < 120 Diastolic < 80)	0	Post menopause onset
BrC411	35	normal	Normal (Systolic < 120 Diastolic < 80)	0	Post menopause onset
BrC412	40	obese	Normal (Systolic < 120 Diastolic < 80)	0	Post menopause onset
BrC413	40	normal	Normal (Systolic < 120 Diastolic < 80)	0	Post menopause onset
BrC414	35	normal	Normal (Systolic < 120 Diastolic < 80)	0	Post menopause onset
BrC415	65	normal	Normal (Systolic < 120 Diastolic < 80)	0	Post menopause onset
BrC416	50	normal	Normal (Systolic < 120 Diastolic < 80)	0	Post menopause onset
BrC417	71	normal	Normal (Systolic < 120 Diastolic < 80)	0	Post menopause onset
BrC418	66	normal	Normal (Systolic < 120 Diastolic < 80)	0	Post menopause onset
BrC419	64	Obese	Normal (Systolic < 120 Diastolic < 80)	0	Pre menopause onset
BrC420	35	Overweight	Normal (Systolic < 120 Diastolic < 80)	0	Pre menopause onset
BrC421	67	Overweight	Normal (Systolic < 120 Diastolic < 80)	0	Post menopause onset
BrC422	36	Obese	Normal (Systolic < 120 Diastolic < 80)	4	Pre menopause onset

BrC423	28	normal	Normal (Systolic < 120 Diastolic < 80)	0	Pre menopause onset
BrC424	60	Overweight	Normal (Systolic < 120 Diastolic < 80)	0	Pre menopause onset
BrC425	65	Overweight	Normal (Systolic < 120 Diastolic < 80)	0	Post menopause onset
BrC426	35	Overweight	Normal (Systolic < 120 Diastolic < 80)	0	Pre menopause onset
BrC427	33	Obese	Normal (Systolic < 120 Diastolic < 80)	0	Post menopause onset
BrC428	26	normal	Normal (Systolic < 120 Diastolic < 80)	2	Pre menopause onset
BrC429	36	Overweight	Normal (Systolic < 120 Diastolic < 80)	0	Pre menopause onset
BrC430	65	Overweight	Normal (Systolic < 120 Diastolic < 80)	0	Pre menopause onset
BrC431	68	Overweight	Normal (Systolic < 120 Diastolic < 80)	0	Pre menopause onset
BrC432	40	normal	Normal (Systolic < 120 Diastolic < 80)	0	Pre menopause onset
BrC433	59	normal	Normal (Systolic < 120 Diastolic < 80)	0	Pre menopause onset
BrC434	35	normal	Normal (Systolic < 120 Diastolic < 80)	3	Post menopause onset
BrC435	63	normal	Normal (Systolic < 120 Diastolic < 80)	0	Pre menopause onset
BrC436	69	normal	Normal (Systolic < 120 Diastolic < 80)	4	Pre menopause onset
BrC437	71	Obese	Normal (Systolic < 120 Diastolic < 80)	0	Pre menopause onset
BrC438	25	Overweight	Normal (Systolic < 120 Diastolic < 80)	0	Pre menopause onset
BrC439	28	normal	Normal (Systolic < 120 Diastolic < 80)	0	Pre menopause onset
BrC440	60	Overweight	Normal (Systolic < 120 Diastolic < 80)	0	Pre menopause onset
BrC441	34	Overweight	Normal (Systolic < 120 Diastolic < 80)	3	Pre menopause onset
BrC442	35	Overweight	Normal (Systolic < 120 Diastolic < 80)	0	Pre menopause onset
BrC443	60	Obese	Normal (Systolic < 120 Diastolic < 80)	0	Pre menopause onset
BrC444	67	Overweight	Normal (Systolic < 120 Diastolic < 80)	0	Pre menopause onset
BrC445	66	Overweight	Normal (Systolic < 120 Diastolic < 80)	4	Pre menopause onset
BrC446	51	normal	Normal (Systolic < 120 Diastolic < 80)	4	Pre menopause onset
BrC447	33	Obese	Normal (Systolic < 120 Diastolic < 80)	0	Pre menopause onset
BrC448	61	Obese	Normal (Systolic < 120 Diastolic < 80)	0	Pre menopause onset
BrC449	71	Obese	Elevated (Systolic 120-129 Diastolic <80)	0	Pre menopause onset
BrC450	62	Overweight	Normal (Systolic < 120 Diastolic < 80)	0	Post menopause onset
BrC451	28	Overweight	Normal (Systolic < 120 Diastolic < 80)	0	Pre menopause onset
BrC452	30	Obese	Normal (Systolic < 120 Diastolic < 80)	0	Pre menopause onset
BrC453	64	Obese	Normal (Systolic < 120 Diastolic < 80)	0	Post menopause onset
BrC454	28	normal	Normal (Systolic < 120 Diastolic < 80)	0	Pre menopause onset
BrC455	65	normal	Normal (Systolic < 120 Diastolic < 80)	0	Pre menopause onset
BrC456	36	normal	Normal (Systolic < 120 Diastolic < 80)	2	Pre menopause onset
BrC457	39	Overweight	Normal (Systolic < 120 Diastolic < 80)	0	Post menopause onset
BrC458	73	Obese	Normal (Systolic < 120 Diastolic < 80)	0	Pre menopause onset
BrC459	28	Overweight	Normal (Systolic < 120 Diastolic < 80)	0	Post menopause onset
BrC460	75	Overweight	Normal (Systolic < 120 Diastolic < 80)	0	Pre menopause onset
BrC461	20	normal	Normal (Systolic < 120 Diastolic < 80)	0	Pre menopause onset
BrC462	62	Obese	Normal (Systolic < 120 Diastolic < 80)	0	Pre menopause onset
BrC463	28	normal	Normal (Systolic < 120 Diastolic < 80)	0	Post menopause onset
BrC464	21	Obese	Normal (Systolic < 120 Diastolic < 80)	4	Post menopause onset
BrC465	28	normal	Normal (Systolic < 120 Diastolic < 80)	0	Post menopause onset
BrC466	34	normal	Normal (Systolic < 120 Diastolic < 80)	0	Post menopause onset
BrC467	65	normal	Normal (Systolic < 120 Diastolic < 80)	0	Post menopause onset
BrC468	28	normal	Normal (Systolic < 120 Diastolic < 80)	0	Pre menopause onset
BrC469	38	normal	Normal (Systolic < 120 Diastolic < 80)	3	Post menopause onset
BrC470	35	Obese	Normal (Systolic < 120 Diastolic < 80)	0	Pre menopause onset
BrC471	38	normal	Normal (Systolic < 120 Diastolic < 80)	0	Post menopause onset
BrC472	40	normal	Normal (Systolic < 120 Diastolic < 80)	0	Post menopause onset
BrC473	34	Overweight	Normal (Systolic < 120 Diastolic < 80)	0	Pre menopause onset
BrC474	35	normal	Normal (Systolic < 120 Diastolic < 80)	0	Post menopause onset
BrC475	62	normal	Normal (Systolic < 120 Diastolic < 80)	0	Pre menopause onset

BrC476	26	normal	Normal (Systolic < 120 Diastolic < 80)	3	Post menopause onset
BrC477	72	normal	Normal (Systolic < 120 Diastolic < 80)	4	Post menopause onset
BrC478	36	normal	Normal (Systolic < 120 Diastolic < 80)	2	Post menopause onset
BrC479	41	Overweight	Normal (Systolic < 120 Diastolic < 80)	0	Pre menopause onset
BrC480	38	Obese	Normal (Systolic < 120 Diastolic < 80)	0	Pre menopause onset
BrC481	43	Obese	Normal (Systolic < 120 Diastolic < 80)	0	Pre menopause onset
BrC482	27	Overweight	Normal (Systolic < 120 Diastolic < 80)	3	Post menopause onset
BrC483	34	Obese	Normal (Systolic < 120 Diastolic < 80)	0	Pre menopause onset
BrC484	35	Obese	Normal (Systolic < 120 Diastolic < 80)	0	Pre menopause onset
BrC485	36	Obese	Normal (Systolic < 120 Diastolic < 80)	0	Post menopause onset
BrC486	73	Overweight	Normal (Systolic < 120 Diastolic < 80)	0	Post menopause onset
BrC487	60	Obese	Normal (Systolic < 120 Diastolic < 80)	0	Post menopause onset
BrC488	71	Obese	Normal (Systolic < 120 Diastolic < 80)	0	Pre menopause onset
BrC489	38	Obese	Normal (Systolic < 120 Diastolic < 80)	0	Pre menopause onset
BrC490	61	Overweight	Normal (Systolic < 120 Diastolic < 80)	0	Post menopause onset
BrC491	23	Obese	Normal (Systolic < 120 Diastolic < 80)	3	Post menopause onset
BrC492	39	Obese	Normal (Systolic < 120 Diastolic < 80)	0	Pre menopause onset
BrC493	57	Obese	Normal (Systolic < 120 Diastolic < 80)	0	Pre menopause onset
BrC494	24	normal	Normal (Systolic < 120 Diastolic < 80)	0	Pre menopause onset
BrC495	41	normal	Normal (Systolic < 120 Diastolic < 80)	0	Pre menopause onset
BrC496	67	normal	Normal (Systolic < 120 Diastolic < 80)	4	Post menopause onset
BrC497	61	normal	Normal (Systolic < 120 Diastolic < 80)	0	Post menopause onset
BrC498	65	normal	Normal (Systolic < 120 Diastolic < 80)	0	Post menopause onset
BrC499	57	normal	Normal (Systolic < 120 Diastolic < 80)	4	Pre menopause onset
BrC500	60	Overweight	Normal (Systolic < 120 Diastolic < 80)	0	Post menopause onset
BrC501	23	Overweight	Normal (Systolic < 120 Diastolic < 80)	2	Post menopause onset
BrC502	73	normal	Normal (Systolic < 120 Diastolic < 80)	4	Post menopause onset
BrC503	27	Obese	Normal (Systolic < 120 Diastolic < 80)	0	Post menopause onset
BrC504	64	Overweight	Normal (Systolic < 120 Diastolic < 80)	0	Post menopause onset
BrC505	68	Overweight	Normal (Systolic < 120 Diastolic < 80)	4	Post menopause onset
BrC506	28	normal	Normal (Systolic < 120 Diastolic < 80)	0	Pre menopause onset
BrC507	37	normal	Normal (Systolic < 120 Diastolic < 80)	0	Post menopause onset
BrC508	57	normal	Normal (Systolic < 120 Diastolic < 80)	0	Pre menopause onset
BrC509	27	normal	Normal (Systolic < 120 Diastolic < 80)	0	Pre menopause onset
BrC510	23	normal	Normal (Systolic < 120 Diastolic < 80)	0	Pre menopause onset
BrC511	41	Overweight	Normal (Systolic < 120 Diastolic < 80)	0	Pre menopause onset
BrC512	42	normal	Normal (Systolic < 120 Diastolic < 80)	0	Pre menopause onset
BrC513	41	normal	Normal (Systolic < 120 Diastolic < 80)	4	Pre menopause onset
BrC514	30	Obese	Normal (Systolic < 120 Diastolic < 80)	0	Post menopause onset
BrC515	43	normal	Normal (Systolic < 120 Diastolic < 80)	0	Pre menopause onset
BrC516	31	normal	Normal (Systolic < 120 Diastolic < 80)	0	Post menopause onset
BrC517	57	normal	Normal (Systolic < 120 Diastolic < 80)	0	Pre menopause onset
BrC518	28	Obese	Normal (Systolic < 120 Diastolic < 80)	0	Pre menopause onset
BrC519	31	Overweight	Normal (Systolic < 120 Diastolic < 80)	0	Post menopause onset
BrC520	55	normal	Normal (Systolic < 120 Diastolic < 80)	0	Post menopause onset
BrC521	38	Overweight	Normal (Systolic < 120 Diastolic < 80)	0	Pre menopause onset
BrC522	51	normal	Normal (Systolic < 120 Diastolic < 80)	0	Pre menopause onset
BrC523	41	Overweight	Normal (Systolic < 120 Diastolic < 80)	0	Pre menopause onset
BrC524	71	Overweight	Normal (Systolic < 120 Diastolic < 80)	0	Post menopause onset
BrC525	29	normal	Normal (Systolic < 120 Diastolic < 80)	0	Post menopause onset
BrC526	67	Overweight	Elevated (Systolic 120-129 Diastolic <80)	1	Pre menopause onset
BrC527	45	normal	Normal (Systolic < 120 Diastolic < 80)	0	Post menopause onset
BrC528	67	Overweight	Normal (Systolic < 120 Diastolic < 80)	0	Pre menopause onset

BrC529	26	Overweight	Normal (Systolic < 120 Diastolic < 80)	0	Pre menopause onset
BrC530	28	normal	Normal (Systolic < 120 Diastolic < 80)	0	Pre menopause onset
BrC531	69	normal	Normal (Systolic < 120 Diastolic < 80)	0	Pre menopause onset
BrC532	29	normal	Normal (Systolic < 120 Diastolic < 80)	4	Post menopause onset
BrC533	26	normal	Normal (Systolic < 120 Diastolic < 80)	0	Pre menopause onset
BrC534	39	normal	Normal (Systolic < 120 Diastolic < 80)	0	Pre menopause onset
BrC535	40	Obese	Normal (Systolic < 120 Diastolic < 80)	0	Pre menopause onset
BrC536	65	Overweight	Normal (Systolic < 120 Diastolic < 80)	0	Pre menopause onset
BrC537	40	normal	Normal (Systolic < 120 Diastolic < 80)	0	Pre menopause onset
BrC538	37	Overweight	Normal (Systolic < 120 Diastolic < 80)	0	Pre menopause onset
BrC539	69	Obese	Normal (Systolic < 120 Diastolic < 80)	0	Pre menopause onset
BrC540	67	normal	Elevated (Systolic 120-129 Diastolic <80)	4	Pre menopause onset
BrC541	70	Overweight	Normal (Systolic < 120 Diastolic < 80)	2	Pre menopause onset
BrC542	67	normal	Normal (Systolic < 120 Diastolic < 80)	0	Pre menopause onset
BrC543	59	Overweight	Normal (Systolic < 120 Diastolic < 80)	0	Pre menopause onset
BrC544	65	Overweight	Normal (Systolic < 120 Diastolic < 80)	0	Pre menopause onset
BrC545	71	normal	Normal (Systolic < 120 Diastolic < 80)	0	Pre menopause onset
BrC546	40	Overweight	Normal (Systolic < 120 Diastolic < 80)	0	Pre menopause onset
BrC547	43	normal	Normal (Systolic < 120 Diastolic < 80)	0	Pre menopause onset
BrC548	67	Overweight	Normal (Systolic < 120 Diastolic < 80)	0	Pre menopause onset
BrC549	39	normal	Normal (Systolic < 120 Diastolic < 80)	0	Post menopause onset
BrC550	46	normal	Normal (Systolic < 120 Diastolic < 80)	0	Pre menopause onset
BrC551	41	normal	Normal (Systolic < 120 Diastolic < 80)	0	Pre menopause onset
BrC552	45	normal	Normal (Systolic < 120 Diastolic < 80)	0	Post menopause onset
BrC553	73	Overweight	Normal (Systolic < 120 Diastolic < 80)	0	Post menopause onset
BrC554	43	normal	Normal (Systolic < 120 Diastolic < 80)	0	Pre menopause onset
BrC555	68	normal	Normal (Systolic < 120 Diastolic < 80)	0	Post menopause onset
BrC556	40	normal	Normal (Systolic < 120 Diastolic < 80)	0	Post menopause onset
BrC557	70	Obese	Normal (Systolic < 120 Diastolic < 80)	0	Pre menopause onset
BrC558	65	normal	Normal (Systolic < 120 Diastolic < 80)	0	Post menopause onset
BrC559	37	Overweight	Normal (Systolic < 120 Diastolic < 80)	0	Pre menopause onset
BrC560	70	Overweight	Normal (Systolic < 120 Diastolic < 80)	0	Post menopause onset
BrC561	73	normal	Normal (Systolic < 120 Diastolic < 80)	2	Pre menopause onset
BrC562	42	Overweight	Normal (Systolic < 120 Diastolic < 80)	0	Post menopause onset
BrC563	72	Obese	Normal (Systolic < 120 Diastolic < 80)	0	Pre menopause onset
BrC564	61	Overweight	Normal (Systolic < 120 Diastolic < 80)	0	Post menopause onset
BrC565	67	normal	Normal (Systolic < 120 Diastolic < 80)	0	Pre menopause onset
BrC566	69	normal	Normal (Systolic < 120 Diastolic < 80)	0	Pre menopause onset
BrC567	72	Overweight	Normal (Systolic < 120 Diastolic < 80)	0	Post menopause onset
BrC568	39	Overweight	Normal (Systolic < 120 Diastolic < 80)	0	Pre menopause onset
BrC569	42	normal	Normal (Systolic < 120 Diastolic < 80)	0	Pre menopause onset
BrC570	63	Overweight	Normal (Systolic < 120 Diastolic < 80)	0	Post menopause onset
BrC571	67	Overweight	Normal (Systolic < 120 Diastolic < 80)	0	Post menopause onset
BrC572	43	Overweight	Normal (Systolic < 120 Diastolic < 80)	0	Pre menopause onset
BrC573	42	Overweight	Normal (Systolic < 120 Diastolic < 80)	0	Post menopause onset
BrC574	65	Obese	Elevated (Systolic 120-129 Diastolic <80)	0	Pre menopause onset
BrC575	40	Obese	Normal (Systolic < 120 Diastolic < 80)	0	Post menopause onset
BrC576	67	Obese	Normal (Systolic < 120 Diastolic < 80)	0	Post menopause onset
BrC577	39	Overweight	Normal (Systolic < 120 Diastolic < 80)	0	Pre menopause onset
BrC578	41	Obese	Normal (Systolic < 120 Diastolic < 80)	0	Pre menopause onset
BrC579	67	Obese	Normal (Systolic < 120 Diastolic < 80)	0	Post menopause onset
BrC580	74	Overweight	Normal (Systolic < 120 Diastolic < 80)	0	Pre menopause onset
BrC581	67	Obese	Normal (Systolic < 120 Diastolic < 80)	0	Pre menopause onset

BrC582	43	Obese	Normal (Systolic < 120 Diastolic < 80)	0	Post menopause onset
BrC583	37	Obese	Normal (Systolic < 120 Diastolic < 80)	4	Pre menopause onset
BrC584	71	Obese	Normal (Systolic < 120 Diastolic < 80)	4	Pre menopause onset
BrC585	67	Overweight	Normal (Systolic < 120 Diastolic < 80)	0	Post menopause onset
BrC586	64	Obese	Normal (Systolic < 120 Diastolic < 80)	0	Pre menopause onset
BrC587	43	Obese	Normal (Systolic < 120 Diastolic < 80)	0	Post menopause onset
BrC588	74	Obese	Normal (Systolic < 120 Diastolic < 80)	3	Pre menopause onset
BrC589	37	Obese	Normal (Systolic < 120 Diastolic < 80)	0	Pre menopause onset
BrC590	64	Obese	Normal (Systolic < 120 Diastolic < 80)	0	Post menopause onset
BrC591	43	Overweight	Normal (Systolic < 120 Diastolic < 80)	0	Post menopause onset
BrC592	65	Obese	Normal (Systolic < 120 Diastolic < 80)	0	Post menopause onset
BrC593	73	Obese	Elevated (Systolic 120-129 Diastolic <80)	2	Pre menopause onset
BrC594	78	Obese	Normal (Systolic < 120 Diastolic < 80)	2	Post menopause onset
BrC595	65	Obese	Normal (Systolic < 120 Diastolic < 80)	3	Pre menopause onset
BrC596	43	Obese	Normal (Systolic < 120 Diastolic < 80)	3	Post menopause onset
BrC597	67	Obese	Normal (Systolic < 120 Diastolic < 80)	2	Pre menopause onset
BrC598	70	Obese	Normal (Systolic < 120 Diastolic < 80)	3	Pre menopause onset
BrC599	65	Obese	Normal (Systolic < 120 Diastolic < 80)	1	Post menopause onset
BrC600	37	Obese	Normal (Systolic < 120 Diastolic < 80)	3	Post menopause onset
BrC601	62	Obese	Normal (Systolic < 120 Diastolic < 80)	2	Post menopause onset
BrC602	56	Obese	Normal (Systolic < 120 Diastolic < 80)	2	Pre menopause onset
BrC603	61	Obese	Normal (Systolic < 120 Diastolic < 80)	3	Pre menopause onset
BrC604	68	Obese	Normal (Systolic < 120 Diastolic < 80)	2	Pre menopause onset
BrC605	70	Obese	Normal (Systolic < 120 Diastolic < 80)	3	Post menopause onset
BrC606	61	Overweight	Normal (Systolic < 120 Diastolic < 80)	1	Post menopause onset
BrC607	37	Obese	Normal (Systolic < 120 Diastolic < 80)	2	Pre menopause onset
BrC608	70	Obese	Normal (Systolic < 120 Diastolic < 80)	2	Post menopause onset
BrC609	36	Obese	Normal (Systolic < 120 Diastolic < 80)	1	Pre menopause onset
BrC610	61	Obese	Normal (Systolic < 120 Diastolic < 80)	1	Post menopause onset
BrC611	64	Obese	Normal (Systolic < 120 Diastolic < 80)	3	Post menopause onset
BrC612	67	Obese	Normal (Systolic < 120 Diastolic < 80)	2	Post menopause onset
BrC613	68	Overweight	Normal (Systolic < 120 Diastolic < 80)	3	Post menopause onset
BrC614	60	Obese	Normal (Systolic < 120 Diastolic < 80)	3	Pre menopause onset
BrC615	59	Obese	Normal (Systolic < 120 Diastolic < 80)	2	Post menopause onset
BrC616	60	Obese	Normal (Systolic < 120 Diastolic < 80)	3	Pre menopause onset
BrC617	37	Obese	Normal (Systolic < 120 Diastolic < 80)	3	Pre menopause onset
BrC618	23	Obese	Normal (Systolic < 120 Diastolic < 80)	3	Post menopause onset
BrC619	73	Overweight	Normal (Systolic < 120 Diastolic < 80)	3	Post menopause onset
BrC620	42	Overweight	Normal (Systolic < 120 Diastolic < 80)	2	Post menopause onset
BrC621	59	normal	Normal (Systolic < 120 Diastolic < 80)	2	Pre menopause onset
BrC622	44	Overweight	Normal (Systolic < 120 Diastolic < 80)	2	Pre menopause onset
BrC623	67	Overweight	Normal (Systolic < 120 Diastolic < 80)	3	Post menopause onset
BrC624	58	normal	Normal (Systolic < 120 Diastolic < 80)	3	Post menopause onset
BrC625	42	normal	Normal (Systolic < 120 Diastolic < 80)	2	Post menopause onset
BrC626	65	normal	Normal (Systolic < 120 Diastolic < 80)	3	Post menopause onset
BrC627	40	normal	Normal (Systolic < 120 Diastolic < 80)	2	Pre menopause onset
BrC628	46	Obese	Normal (Systolic < 120 Diastolic < 80)	3	Post menopause onset
BrC629	56	Obese	Normal (Systolic < 120 Diastolic < 80)	3	Post menopause onset
BrC630	71	Obese	Normal (Systolic < 120 Diastolic < 80)	2	Post menopause onset
BrC631	42	Overweight	Normal (Systolic < 120 Diastolic < 80)	2	Post menopause onset
BrC632	36	normal	Normal (Systolic < 120 Diastolic < 80)	2	Pre menopause onset
BrC633	37	Obese	Normal (Systolic < 120 Diastolic < 80)	2	Post menopause onset
BrC634	70	normal	Normal (Systolic < 120 Diastolic < 80)	1	Pre menopause onset

BrC635	60	Obese	Normal (Systolic < 120 Diastolic < 80)	2	Post menopause onset
BrC636	41	normal	Normal (Systolic < 120 Diastolic < 80)	3	Pre menopause onset
BrC637	60	normal	Normal (Systolic < 120 Diastolic < 80)	3	Post menopause onset
BrC638	64	normal	Normal (Systolic < 120 Diastolic < 80)	2	Pre menopause onset
BrC639	43	normal	Normal (Systolic < 120 Diastolic < 80)	2	Pre menopause onset
BrC640	41	normal	Normal (Systolic < 120 Diastolic < 80)	2	Pre menopause onset
BrC641	46	normal	Normal (Systolic < 120 Diastolic < 80)	2	Post menopause onset
BrC642	38	normal	Normal (Systolic < 120 Diastolic < 80)	3	Post menopause onset
BrC643	71	normal	Normal (Systolic < 120 Diastolic < 80)	2	Post menopause onset
BrC644	38	normal	Normal (Systolic < 120 Diastolic < 80)	2	Post menopause onset
BrC645	60	Overweight	Normal (Systolic < 120 Diastolic < 80)	3	Post menopause onset
BrC646	39	normal	Normal (Systolic < 120 Diastolic < 80)	2	Pre menopause onset
BrC647	60	normal	Normal (Systolic < 120 Diastolic < 80)	2	Pre menopause onset
BrC648	40	normal	Normal (Systolic < 120 Diastolic < 80)	2	Pre menopause onset
BrC649	37	normal	Normal (Systolic < 120 Diastolic < 80)	2	Pre menopause onset
BrC650	61	Overweight	Normal (Systolic < 120 Diastolic < 80)	2	Post menopause onset
