# Supplementary material for: Linkage disequilibrium block single-nucleotide polymorphisms in FTO alpha ketoglutarate dependent dioxygenase gene inference with breast cancer and Type II diabetes in Pakistani female population
Source: PLoS One. 2023 Jul 20;18(7):e0288934. doi: 10.1371/journal.pone.0288934 (PMC10358933; doi:10.1371/journal.pone.0288934)
Supplement: S2 File — (PDF) [file pone.0288934.s002.pdf]

S2: Minimal Data Set for Healthy Breast Cancer Controls

Sample ID	Age in Years	BMI Status	Hypertension (mmHg)	Menopause
CBrC001	35	Normal	Normal (Systolic < 120 Diastolic < 80)	Pre Menopause
CBrC002	25	Normal	Normal (Systolic < 120 Diastolic < 80)	Pre Menopause
CBrC003	31	Normal	Normal (Systolic < 120 Diastolic < 80)	Pre Menopause
CBrC004	24	Normal	Normal (Systolic < 120 Diastolic < 80)	Pre Menopause
CBrC005	40	Normal	Normal (Systolic < 120 Diastolic < 80)	Pre Menopause
CBrC006	42	Normal	Normal (Systolic < 120 Diastolic < 80)	Pre Menopause
CBrC007	40	Normal	Normal (Systolic < 120 Diastolic < 80)	Pre Menopause
CBrC008	35	Normal	Normal (Systolic < 120 Diastolic < 80)	Pre Menopause
CBrC009	42	Normal	Normal (Systolic < 120 Diastolic < 80)	Pre Menopause
CBrC010	60	Normal	Normal (Systolic < 120 Diastolic < 80)	Pre Menopause
CBrC011	35	Normal	Normal (Systolic < 120 Diastolic < 80)	Pre Menopause
CBrC012	30	Normal	Normal (Systolic < 120 Diastolic < 80)	Pre Menopause
CBrC013	27	Normal	Normal (Systolic < 120 Diastolic < 80)	Pre Menopause
CBrC014	70	Normal	Normal (Systolic < 120 Diastolic < 80)	Pre Menopause
CBrC015	26	Normal	Normal (Systolic < 120 Diastolic < 80)	Pre Menopause
CBrC016	32	Normal	Normal (Systolic < 120 Diastolic < 80)	Pre Menopause
CBrC017	31	Normal	Normal (Systolic < 120 Diastolic < 80)	Pre Menopause
CBrC018	42	Normal	Normal (Systolic < 120 Diastolic < 80)	Pre Menopause
CBrC019	50	Normal	Normal (Systolic < 120 Diastolic < 80)	Pre Menopause
CBrC020	30	Normal	Normal (Systolic < 120 Diastolic < 80)	Pre Menopause
CBrC021	29	Normal	Normal (Systolic < 120 Diastolic < 80)	Pre Menopause
CBrC022	31	Normal	Normal (Systolic < 120 Diastolic < 80)	Pre Menopause
CBrC023	70	Normal	Normal (Systolic < 120 Diastolic < 80)	Pre Menopause
CBrC024	26	Normal	Normal (Systolic < 120 Diastolic < 80)	Pre Menopause
CBrC025	28	Over Weight	Normal (Systolic < 120 Diastolic < 80)	Pre Menopause
CBrC026	24	Normal	Normal (Systolic < 120 Diastolic < 80)	Pre Menopause
CBrC027	42	Normal	Normal (Systolic < 120 Diastolic < 80)	Pre Menopause
CBrC028	30	Normal	Normal (Systolic < 120 Diastolic < 80)	Pre Menopause
CBrC029	70	Normal	Normal (Systolic < 120 Diastolic < 80)	Pre Menopause
CBrC030	69	Normal	Normal (Systolic < 120 Diastolic < 80)	Pre Menopause
CBrC031	52	Normal	Normal (Systolic < 120 Diastolic < 80)	Pre Menopause
CBrC032	68	Normal	Normal (Systolic < 120 Diastolic < 80)	Pre Menopause
CBrC033	32	Normal	Normal (Systolic < 120 Diastolic < 80)	Pre Menopause
CBrC034	67	Normal	Normal (Systolic < 120 Diastolic < 80)	Pre Menopause
CBrC035	31	Normal	Normal (Systolic < 120 Diastolic < 80)	Pre Menopause
CBrC036	64	Normal	Elevated (Systolic 120-129 Diastolic <80)	Pre Menopause
CBrC037	71	Normal	Normal (Systolic < 120 Diastolic < 80)	Pre Menopause
CBrC038	27	Normal	Normal (Systolic < 120 Diastolic < 80)	Pre Menopause
CBrC039	56	Normal	Normal (Systolic < 120 Diastolic < 80)	Pre Menopause
CBrC040	26	Normal	Normal (Systolic < 120 Diastolic < 80)	Pre Menopause
CBrC041	68	Normal	Normal (Systolic < 120 Diastolic < 80)	Pre Menopause
CBrC042	34	Normal	Normal (Systolic < 120 Diastolic < 80)	Pre Menopause
CBrC043	51	Normal	Normal (Systolic < 120 Diastolic < 80)	Pre Menopause
CBrC044	34	Normal	Normal (Systolic < 120 Diastolic < 80)	Pre Menopause
CBrC045	50	Normal	Normal (Systolic < 120 Diastolic < 80)	Pre Menopause
CBrC046	65	Normal	Normal (Systolic < 120 Diastolic < 80)	Pre Menopause
CBrC047	66	Normal	Normal (Systolic < 120 Diastolic < 80)	Pre Menopause

CBrC048	60	Normal	Normal (Systolic < 120 Diastolic < 80)	Pre Menopause
CBrC049	70	Normal	Normal (Systolic < 120 Diastolic < 80)	Pre Menopause
CBrC050	62	Normal	Normal (Systolic < 120 Diastolic < 80)	Pre Menopause
CBrC051	69	Normal	Normal (Systolic < 120 Diastolic < 80)	Pre Menopause
CBrC052	65	Normal	Normal (Systolic < 120 Diastolic < 80)	Pre Menopause
CBrC053	59	Normal	Normal (Systolic < 120 Diastolic < 80)	Pre Menopause
CBrC054	56	Normal	Normal (Systolic < 120 Diastolic < 80)	Pre Menopause
CBrC055	62	Normal	Normal (Systolic < 120 Diastolic < 80)	Pre Menopause
CBrC056	59	Normal	Normal (Systolic < 120 Diastolic < 80)	Pre Menopause
CBrC057	45	Normal	Normal (Systolic < 120 Diastolic < 80)	Pre Menopause
CBrC058	64	Normal	Normal (Systolic < 120 Diastolic < 80)	Pre Menopause
CBrC059	59	Normal	Normal (Systolic < 120 Diastolic < 80)	Pre Menopause
CBrC060	65	Normal	Normal (Systolic < 120 Diastolic < 80)	Pre Menopause
CBrC061	49	Over Weight	Normal (Systolic < 120 Diastolic < 80)	Pre Menopause
CBrC062	70	Normal	Normal (Systolic < 120 Diastolic < 80)	Pre Menopause
CBrC063	49	Normal	Normal (Systolic < 120 Diastolic < 80)	Pre Menopause
CBrC064	62	Normal	Elevated (Systolic 120-129 Diastolic <80)	Pre Menopause
CBrC065	69	Normal	Normal (Systolic < 120 Diastolic < 80)	Pre Menopause
CBrC066	68	Normal	Normal (Systolic < 120 Diastolic < 80)	Nil
CBrC067	65	Normal	Normal (Systolic < 120 Diastolic < 80)	Pre Menopause
CBrC068	45	Normal	Normal (Systolic < 120 Diastolic < 80)	Pre Menopause
CBrC069	48	Normal	Normal (Systolic < 120 Diastolic < 80)	Pre Menopause
CBrC070	46	Normal	Normal (Systolic < 120 Diastolic < 80)	Post Menopause
CBrC071	37	Normal	Normal (Systolic < 120 Diastolic < 80)	Post Menopause
CBrC072	42	Normal	Normal (Systolic < 120 Diastolic < 80)	Post Menopause
CBrC073	61	Normal	Normal (Systolic < 120 Diastolic < 80)	Post Menopause
CBrC074	70	Normal	Normal (Systolic < 120 Diastolic < 80)	Pre Menopause
CBrC075	35	Normal	Normal (Systolic < 120 Diastolic < 80)	Post Menopause
CBrC076	32	Normal	Normal (Systolic < 120 Diastolic < 80)	Post Menopause
CBrC077	61	Normal	Normal (Systolic < 120 Diastolic < 80)	Post Menopause
CBrC078	27	Normal	Normal (Systolic < 120 Diastolic < 80)	Post Menopause
CBrC079	67	Normal	Normal (Systolic < 120 Diastolic < 80)	Post Menopause
CBrC080	38	Normal	Elevated (Systolic 120-129 Diastolic <80)	Pre Menopause
CBrC081	29	Normal	Normal (Systolic < 120 Diastolic < 80)	Post Menopause
CBrC082	71	Normal	Normal (Systolic < 120 Diastolic < 80)	Post Menopause
CBrC083	29	Normal	Normal (Systolic < 120 Diastolic < 80)	Post Menopause
CBrC084	38	Normal	Normal (Systolic < 120 Diastolic < 80)	Post Menopause
CBrC085	64	Normal	Normal (Systolic < 120 Diastolic < 80)	Post Menopause
CBrC086	36	Normal	Normal (Systolic < 120 Diastolic < 80)	Pre Menopause
CBrC087	28	Normal	Normal (Systolic < 120 Diastolic < 80)	Post Menopause
CBrC088	33	Normal	Normal (Systolic < 120 Diastolic < 80)	Pre Menopause
CBrC089	65	Normal	Normal (Systolic < 120 Diastolic < 80)	Post Menopause
CBrC090	56	Normal	Normal (Systolic < 120 Diastolic < 80)	Post Menopause
CBrC091	68	Normal	Normal (Systolic < 120 Diastolic < 80)	Post Menopause
CBrC092	61	Normal	Normal (Systolic < 120 Diastolic < 80)	Post Menopause
CBrC093	29	Normal	Normal (Systolic < 120 Diastolic < 80)	Post Menopause
CBrC094	37	Normal	Normal (Systolic < 120 Diastolic < 80)	Post Menopause
CBrC095	39	Normal	Normal (Systolic < 120 Diastolic < 80)	Post Menopause
CBrC096	65	Normal	Normal (Systolic < 120 Diastolic < 80)	Post Menopause

CBrC097	56	Normal	Normal (Systolic < 120 Diastolic < 80)	Post Menopause
CBrC098	49	Normal	Normal (Systolic < 120 Diastolic < 80)	Pre Menopause
CBrC099	23	Over Weight	Normal (Systolic < 120 Diastolic < 80)	Post Menopause
CBrC100	26	Normal	Normal (Systolic < 120 Diastolic < 80)	Post Menopause
CBrC101	36	Normal	Normal (Systolic < 120 Diastolic < 80)	Post Menopause
CBrC102	28	Normal	Normal (Systolic < 120 Diastolic < 80)	Post Menopause
CBrC103	37	Normal	Normal (Systolic < 120 Diastolic < 80)	Post Menopause
CBrC104	53	Normal	Normal (Systolic < 120 Diastolic < 80)	Post Menopause
CBrC105	38	Normal	Normal (Systolic < 120 Diastolic < 80)	Pre Menopause
CBrC106	21	Normal	Normal (Systolic < 120 Diastolic < 80)	Post Menopause
CBrC107	71	Normal	Normal (Systolic < 120 Diastolic < 80)	Pre Menopause
CBrC108	57	Normal	Normal (Systolic < 120 Diastolic < 80)	Post Menopause
CBrC109	59	Normal	Normal (Systolic < 120 Diastolic < 80)	Post Menopause
CBrC110	51	Normal	Elevated (Systolic 120-129 Diastolic <80)	Post Menopause
CBrC111	37	Normal	Normal (Systolic < 120 Diastolic < 80)	Post Menopause
CBrC112	56	Normal	Normal (Systolic < 120 Diastolic < 80)	Post Menopause
CBrC113	63	Normal	Normal (Systolic < 120 Diastolic < 80)	Post Menopause
CBrC114	36	Normal	Normal (Systolic < 120 Diastolic < 80)	Post Menopause
CBrC115	36	Normal	Normal (Systolic < 120 Diastolic < 80)	Post Menopause
CBrC116	60	Normal	Normal (Systolic < 120 Diastolic < 80)	Post Menopause
CBrC117	50	Normal	Normal (Systolic < 120 Diastolic < 80)	Post Menopause
CBrC118	54	Normal	Normal (Systolic < 120 Diastolic < 80)	Post Menopause
CBrC119	48	Normal	Normal (Systolic < 120 Diastolic < 80)	Pre Menopause
CBrC120	27	Normal	Normal (Systolic < 120 Diastolic < 80)	Post Menopause
CBrC121	73	Normal	Normal (Systolic < 120 Diastolic < 80)	Post Menopause
CBrC122	51	Normal	Normal (Systolic < 120 Diastolic < 80)	Post Menopause
CBrC123	36	Normal	Normal (Systolic < 120 Diastolic < 80)	Post Menopause
CBrC124	38	Normal	Normal (Systolic < 120 Diastolic < 80)	Pre Menopause
CBrC125	55	Normal	Normal (Systolic < 120 Diastolic < 80)	Post Menopause
CBrC126	40	Normal	Normal (Systolic < 120 Diastolic < 80)	Post Menopause
CBrC127	68	Normal	Normal (Systolic < 120 Diastolic < 80)	Post Menopause
CBrC128	24	Normal	Normal (Systolic < 120 Diastolic < 80)	Post Menopause
CBrC129	56	Normal	Normal (Systolic < 120 Diastolic < 80)	Post Menopause
CBrC130	65	Normal	Elevated (Systolic 120-129 Diastolic <80)	Post Menopause
CBrC131	39	Normal	Normal (Systolic < 120 Diastolic < 80)	Post Menopause
CBrC132	53	Normal	Normal (Systolic < 120 Diastolic < 80)	Post Menopause
CBrC133	39	Normal	Normal (Systolic < 120 Diastolic < 80)	Pre Menopause
CBrC134	56	Normal	Normal (Systolic < 120 Diastolic < 80)	Post Menopause
CBrC135	33	Over Weight	Normal (Systolic < 120 Diastolic < 80)	Post Menopause
CBrC136	51	Normal	Normal (Systolic < 120 Diastolic < 80)	Post Menopause
CBrC137	49	Normal	Normal (Systolic < 120 Diastolic < 80)	Post Menopause
CBrC138	37	Normal	Normal (Systolic < 120 Diastolic < 80)	Post Menopause
CBrC139	56	Normal	Normal (Systolic < 120 Diastolic < 80)	Pre Menopause
CBrC140	57	Normal	Normal (Systolic < 120 Diastolic < 80)	Nil
CBrC141	34	Normal	Normal (Systolic < 120 Diastolic < 80)	Post Menopause
CBrC142	54	Normal	Normal (Systolic < 120 Diastolic < 80)	Post Menopause
CBrC143	41	Normal	Normal (Systolic < 120 Diastolic < 80)	Post Menopause
CBrC144	29	Normal	Normal (Systolic < 120 Diastolic < 80)	Post Menopause
CBrC145	55	Normal	Normal (Systolic < 120 Diastolic < 80)	Post Menopause

CBrC146	67	Normal	Normal (Systolic < 120 Diastolic < 80)	Post Menopause
CBrC147	58	Normal	Normal (Systolic < 120 Diastolic < 80)	Post Menopause
CBrC148	53	Over Weight	Normal (Systolic < 120 Diastolic < 80)	Post Menopause
CBrC149	50	Normal	Normal (Systolic < 120 Diastolic < 80)	Post Menopause
CBrC150	46	Normal	Elevated (Systolic 120-129 Diastolic <80)	Post Menopause
CBrC151	46	Normal	Normal (Systolic < 120 Diastolic < 80)	Post Menopause
CBrC152	33	Normal	Normal (Systolic < 120 Diastolic < 80)	Post Menopause
CBrC153	40	Normal	Normal (Systolic < 120 Diastolic < 80)	Post Menopause
CBrC154	38	Normal	Normal (Systolic < 120 Diastolic < 80)	Post Menopause
CBrC155	50	Normal	Normal (Systolic < 120 Diastolic < 80)	Pre Menopause
CBrC156	64	Normal	Normal (Systolic < 120 Diastolic < 80)	Post Menopause
CBrC157	61	Normal	Normal (Systolic < 120 Diastolic < 80)	Post Menopause
CBrC158	66	Normal	Normal (Systolic < 120 Diastolic < 80)	Post Menopause
CBrC159	63	Normal	Normal (Systolic < 120 Diastolic < 80)	Post Menopause
CBrC160	49	Normal	Normal (Systolic < 120 Diastolic < 80)	Post Menopause
CBrC161	53	Normal	Normal (Systolic < 120 Diastolic < 80)	Post Menopause
CBrC162	51	Normal	Elevated (Systolic 120-129 Diastolic <80)	Post Menopause
CBrC163	31	Normal	Normal (Systolic < 120 Diastolic < 80)	Post Menopause
CBrC164	53	Normal	Normal (Systolic < 120 Diastolic < 80)	Post Menopause
CBrC165	51	Normal	Normal (Systolic < 120 Diastolic < 80)	Post Menopause
CBrC166	70	Normal	Normal (Systolic < 120 Diastolic < 80)	Post Menopause
CBrC167	51	Normal	Normal (Systolic < 120 Diastolic < 80)	Post Menopause
CBrC168	36	Normal	Normal (Systolic < 120 Diastolic < 80)	Post Menopause
CBrC169	74	Normal	Normal (Systolic < 120 Diastolic < 80)	Post Menopause
CBrC170	31	Normal	Normal (Systolic < 120 Diastolic < 80)	Post Menopause
CBrC171	60	Normal	Normal (Systolic < 120 Diastolic < 80)	Post Menopause
CBrC172	30	Normal	Normal (Systolic < 120 Diastolic < 80)	Post Menopause
CBrC173	27	Normal	Normal (Systolic < 120 Diastolic < 80)	Post Menopause
CBrC174	23	Normal	Normal (Systolic < 120 Diastolic < 80)	Post Menopause
CBrC175	38	Normal	Normal (Systolic < 120 Diastolic < 80)	Post Menopause
CBrC176	56	Normal	Normal (Systolic < 120 Diastolic < 80)	Post Menopause
CBrC177	23	Normal	Elevated (Systolic 120-129 Diastolic <80)	Post Menopause
CBrC178	20	Normal	Normal (Systolic < 120 Diastolic < 80)	Post Menopause
CBrC179	49	Normal	Normal (Systolic < 120 Diastolic < 80)	Post Menopause
CBrC180	36	Normal	Normal (Systolic < 120 Diastolic < 80)	Post Menopause
CBrC181	36	Normal	Elevated (Systolic 120-129 Diastolic <80)	Post Menopause
CBrC182	49	Normal	Normal (Systolic < 120 Diastolic < 80)	Post Menopause
CBrC183	32	Normal	Normal (Systolic < 120 Diastolic < 80)	Pre Menopause
CBrC184	61	Normal	Normal (Systolic < 120 Diastolic < 80)	Post Menopause
CBrC185	70	Normal	Normal (Systolic < 120 Diastolic < 80)	Post Menopause
CBrC186	61	Normal	Elevated (Systolic 120-129 Diastolic <80)	Post Menopause
CBrC187	40	Normal	Normal (Systolic < 120 Diastolic < 80)	Post Menopause
CBrC188	54	Normal	Normal (Systolic < 120 Diastolic < 80)	Post Menopause
CBrC189	22	Normal	Normal (Systolic < 120 Diastolic < 80)	Post Menopause
CBrC190	49	Normal	Normal (Systolic < 120 Diastolic < 80)	Post Menopause
CBrC191	34	Normal	Normal (Systolic < 120 Diastolic < 80)	Post Menopause
CBrC192	49	Normal	Normal (Systolic < 120 Diastolic < 80)	Post Menopause
CBrC193	40	Normal	Normal (Systolic < 120 Diastolic < 80)	Post Menopause
CBrC194	40	Normal	Elevated (Systolic 120-129 Diastolic <80)	Post Menopause

CBrC195	33	Normal	Normal (Systolic < 120 Diastolic < 80)	Post Menopause
CBrC196	50	Normal	Normal (Systolic < 120 Diastolic < 80)	Post Menopause
CBrC197	55	Normal	Normal (Systolic < 120 Diastolic < 80)	Post Menopause
CBrC198	59	Normal	Normal (Systolic < 120 Diastolic < 80)	Pre Menopause
CBrC199	53	Normal	Normal (Systolic < 120 Diastolic < 80)	Post Menopause
CBrC200	39	Normal	Normal (Systolic < 120 Diastolic < 80)	Post Menopause
