# Supplementary material for: Linkage disequilibrium block single-nucleotide polymorphisms in FTO alpha ketoglutarate dependent dioxygenase gene inference with breast cancer and Type II diabetes in Pakistani female population
Source: PLoS One. 2023 Jul 20;18(7):e0288934. doi: 10.1371/journal.pone.0288934 (PMC10358933; doi:10.1371/journal.pone.0288934)
Supplement: S3 File — (PDF) [file pone.0288934.s003.pdf]

S3: Minimal Data Set for Type II diabetes

Sample ID	Age in Years	Gender	BMI status	Sugar Profile (Fasting, mg/dL)	Sugar Profile (Random, mg/dL)	Cholesterol (mg/dL)	Triglyceride (mg/dL)	LDL (mg/dL)	HDL (mg/dL)	Hypertension Status
NIDDM001	28	Female	Obese	269	117	225	80	80	23	Hypertension Stage 2 (Systolic >140 Diastolic >90)
NIDDM002	60	Female	Obese	265	119	224	51	70	53	Hypertension Stage 2 (Systolic >140 Diastolic >90)
NIDDM003	42	Male	Over Weight	234	382	216	380	75	41	Hypertension Stage 2 (Systolic >140 Diastolic >90)
NIDDM004	43	Female	Over Weight	301	114	214	52	56	11	Hypertension Stage 2 (Systolic >140 Diastolic >90)
NIDDM005	32	Female	Normal	259	426	180	100	79	14	Normal (Systolic < 120 Diastolic < 80)
NIDDM006	61	Male	Over Weight	195	122	205	90	40	24	Hypertension Stage 2 (Systolic >140 Diastolic >90)
NIDDM007	67	Female	Over Weight	169	125	207	55	78	25	Hypertension Stage 2 (Systolic >140 Diastolic >90)
NIDDM008	65	Female	Normal	256	563	177	45	82	49	Normal (Systolic < 120 Diastolic < 80)
NIDDM009	60	Female	Normal	256	115	179	46	112	18	Hypertension Stage 2 (Systolic >140 Diastolic >90)
NIDDM010	25	Female	Over Weight	258	185	212	390	46	20	Hypertension Stage 2 (Systolic >140 Diastolic >90)
NIDDM011	68	Male	Normal	369	152	195	67	125	20	Hypertension Stage 2 (Systolic >140 Diastolic >90)
NIDDM012	72	Female	Over Weight	129	112	213	390	95	21	Hypertension Stage 2 (Systolic >140 Diastolic >90)
NIDDM013	25	Female	Normal	258	223	150	310	79	50	Hypertension Stage 2 (Systolic >140 Diastolic >90)
NIDDM014	35	Female	Over Weight	222	456	170	80	90	12	Hypertension Stage 2 (Systolic >140 Diastolic >90)
NIDDM015	69	Female	Over Weight	228	269	178	62	86	70	Elevated (Systolic 120-129 Diastolic <80)
NIDDM016	31	Female	Over Weight	268	245	200	60	95	40	Hypertension Stage 2 (Systolic >140 Diastolic >90)
NIDDM017	41	Male	Obese	269	215	310	56	75	47	Hypertension Stage 2 (Systolic >140 Diastolic >90)
NIDDM018	29	Female	Normal	254	360	150	76	43	50	Normal (Systolic < 120 Diastolic < 80)
NIDDM019	72	Female	Over Weight	192	360	170	312	70	27	Hypertension Stage 2 (Systolic >140 Diastolic >90)
NIDDM020	32	Female	Over Weight	245	123	120	318	60	76	Hypertension Stage 2 (Systolic >140 Diastolic >90)
NIDDM021	28	Female	Normal	186	256	100	40	78	42	Hypertension Stage 2 (Systolic >140 Diastolic >90)
NIDDM022	35	Female	Normal	145	211	99	90	50	60	Hypertension Stage 2 (Systolic >140 Diastolic >90)
NIDDM023	71	Female	Over Weight	325	152	182	80	54	14	Hypertension Stage 2 (Systolic >140 Diastolic >90)
NIDDM024	60	Male	Normal	256	217	101	78	50	16	Hypertension Stage 2 (Systolic >140 Diastolic >90)
NIDDM025	70	Female	Normal	158	214	105	310	69	70	Hypertension Stage 2 (Systolic >140 Diastolic >90)
NIDDM026	71	Female	Normal	152	125	100	90	102	11	Hypertension Stage 2 (Systolic >140 Diastolic >90)
NIDDM027	35	Female	Obese	169	150	181	56	80	12	Normal (Systolic < 120 Diastolic < 80)
NIDDM028	68	Female	Normal	241	174	100	67	45	33	Normal (Systolic < 120 Diastolic < 80)
NIDDM029	41	Male	Obese	211	129	180	321	117	13	Hypertension Stage 2 (Systolic >140 Diastolic >90)
NIDDM030	35	Female	Normal	201	281	182	90	88	12	Hypertension Stage 2 (Systolic >140 Diastolic >90)
NIDDM031	28	Female	Over Weight	200	214	182	56	105	15	Hypertension Stage 2 (Systolic >140 Diastolic >90)
NIDDM032	32	Female	Over Weight	205	218	184	80	120	19	Hypertension Stage 2 (Systolic >140 Diastolic >90)
NIDDM033	70	Female	Normal	210	360	100	66	121	110	Normal (Systolic < 120 Diastolic < 80)
NIDDM034	65	Male	Normal	185	128	112	47	105	15	Normal (Systolic < 120 Diastolic < 80)
NIDDM035	43	Male	Over Weight	123	216	185	45	116	55	Elevated (Systolic 120-129 Diastolic <80)
NIDDM036	56	Female	Obese	126	213	160	321	70	134	Hypertension Stage 2 (Systolic >140 Diastolic >90)
NIDDM037	59	Female	Obese	200	224	187	68	117	90	Hypertension Stage 2 (Systolic >140 Diastolic >90)
NIDDM038	46	Female	Over Weight	200	214	187	80	94	25	Hypertension Stage 2 (Systolic >140 Diastolic >90)
NIDDM039	59	Female	Normal	135	263	120	76	140	23	Normal (Systolic < 120 Diastolic < 80)
NIDDM040	58	Female	Obese	256	224	190	104	130	13	Normal (Systolic < 120 Diastolic < 80)
NIDDM041	58	Female	Over Weight	269	221	190	90	90	19	Normal (Systolic < 120 Diastolic < 80)
NIDDM042	53	Female	Over Weight	360	214	181	68	85	11	Hypertension Stage 2 (Systolic >140 Diastolic >90)
NIDDM043	50	Male	Over Weight	305	216	182	78	83	19	Normal (Systolic < 120 Diastolic < 80)
NIDDM044	40	Female	Normal	156	214	182	54	108	17	Normal (Systolic < 120 Diastolic < 80)
NIDDM045	52	Female	Obese	236	224	190	310	136	10	Hypertension Stage 2 (Systolic >140 Diastolic >90)
NIDDM046	55	Male	Over Weight	268	225	140	48	130	65	Normal (Systolic < 120 Diastolic < 80)
NIDDM047	45	Female	Over Weight	245	218	150	73	99	55	Normal (Systolic < 120 Diastolic < 80)
NIDDM048	62	Male	Over Weight	206	217	167	410	91	54	Hypertension Stage 2 (Systolic >140 Diastolic >90)
NIDDM049	46	Male	Over Weight	175	213	182	310	107	25	Hypertension Stage 2 (Systolic >140 Diastolic >90)
NIDDM050	47	Female	Over Weight	162	216	145	40	43	14	Hypertension Stage 2 (Systolic >140 Diastolic >90)
NIDDM051	46	Female	Over Weight	169	223	190	71	40	38	Normal (Systolic < 120 Diastolic < 80)
NIDDM052	49	Female	Obese	123	127	170	89	70	17	Hypertension Stage 2 (Systolic >140 Diastolic >90)
NIDDM053	41	Female	Over Weight	123	224	150	79	41	74	Hypertension Stage 2 (Systolic >140 Diastolic >90)
NIDDM054	45	Female	Normal	287	214	110	56	129	31	Hypertension Stage 2 (Systolic >140 Diastolic >90)
NIDDM055	51	Male	Obese	215	216	200	300	210	21	Normal (Systolic < 120 Diastolic < 80)
NIDDM056	47	Female	Normal	280	213	182	80	125	35	Normal (Systolic < 120 Diastolic < 80)
NIDDM057	49	Female	Over Weight	169	120	205	43	83	46	Elevated (Systolic 120-129 Diastolic <80)
NIDDM058	59	Female	Over Weight	260	215	204	310	96	26	Hypertension Stage 2 (Systolic >140 Diastolic >90)
NIDDM059	49	Male	Normal	236	459	110	54	58	46	Normal (Systolic < 120 Diastolic < 80)
NIDDM060	54	Female	Over Weight	189	214	202	360	90	122	Hypertension Stage 2 (Systolic >140 Diastolic >90)
NIDDM061	42	Male	Obese	190	215	200	378	95	45	Hypertension Stage 2 (Systolic >140 Diastolic >90)
NIDDM062	52	Female	Normal	191	221	145	90	85	29	Normal (Systolic < 120 Diastolic < 80)
NIDDM063	41	Male	Normal	165	469	134	80	123	29	Hypertension Stage 2 (Systolic >140 Diastolic >90)
NIDDM064	56	Female	Normal	124	123	127	340	89	50	Hypertension Stage 2 (Systolic >140 Diastolic >90)
NIDDM065	46	Female	Over Weight	193	123	182	90	90	47	Hypertension Stage 2 (Systolic >140 Diastolic >90)
NIDDM066	61	Male	Obese	185	218	180	390	123	18	Hypertension Stage 2 (Systolic >140 Diastolic >90)
NIDDM067	41	Male	Over Weight	340	213	184	291	78	54	Normal (Systolic < 120 Diastolic < 80)
NIDDM068	42	Female	Over Weight	190	224	167	356	90	34	Hypertension Stage 2 (Systolic >140 Diastolic >90)
NIDDM069	54	Female	Normal	300	221	167	90	127	45	Normal (Systolic < 120 Diastolic < 80)
NIDDM070	55	Female	Normal	203	360	200	160	90	40	Normal (Systolic < 120 Diastolic < 80)
NIDDM071	49	Male	Obese	127	115	190	152	73	43	Normal (Systolic < 120 Diastolic < 80)
NIDDM072	60	Male	Over Weight	282	280	201	92	125	42	Normal (Systolic < 120 Diastolic < 80)
NIDDM073	40	Female	Normal	109	279	105	61	98	40	Normal (Systolic < 120 Diastolic < 80)
NIDDM074	61	Female	Normal	221	345	123	186	101	39	Elevated (Systolic 120-129 Diastolic <80)
NIDDM075	61	Male	Over Weight	326	267	177	354	98	45	Normal (Systolic < 120 Diastolic < 80)
NIDDM076	60	Female	Over Weight	100	276	140	94	133	39	Normal (Systolic < 120 Diastolic < 80)
NIDDM077	67	Female	Over Weight	166	216	112	196	122	36	Normal (Systolic < 120 Diastolic < 80)
NIDDM078	29	Male	Normal	231	251	112	120	108	49	Elevated (Systolic 120-129 Diastolic <80)
NIDDM079	64	Male	Normal	150	215	121	340	123	38	Normal (Systolic < 120 Diastolic < 80)
NIDDM080	72	Female	Over Weight	141	218	190	90	150	50	Normal (Systolic < 120 Diastolic < 80)
NIDDM081	71	Female	Normal	125	308	110	312	145	20	Normal (Systolic < 120 Diastolic < 80)

NIDDM082	61	Female	Normal	204	216	100	390	78	54	Elevated (Systolic 120-129 Diastolic <80)
NIDDM083	46	Female	Over Weight	125	223	182	0.9	179	52	Normal (Systolic < 120 Diastolic < 80)
NIDDM084	47	Male	Obese	169	261	186	144	88	36	Normal (Systolic < 120 Diastolic < 80)
NIDDM085	51	Male	Normal	129	495	107	413	160	40	Normal (Systolic < 120 Diastolic < 80)
NIDDM086	60	Female	Over Weight	201	469	180	404	60	55	Normal (Systolic < 120 Diastolic < 80)
NIDDM087	47	Male	Over Weight	288	236	145	80	140	17	Normal (Systolic < 120 Diastolic < 80)
NIDDM088	61	Female	Over Weight	118	336	167	188	175	41	Normal (Systolic < 120 Diastolic < 80)
NIDDM089	59	Female	Obese	180	375	198	45	123	59	Normal (Systolic < 120 Diastolic < 80)
NIDDM090	45	Female	Normal	90	256	107	310	102	24	Normal (Systolic < 120 Diastolic < 80)
NIDDM091	50	Female	Over Weight	159	277	203	47	50	13	Normal (Systolic < 120 Diastolic < 80)
NIDDM092	58	Female	Normal	170	142	182	367	101	41	Normal (Systolic < 120 Diastolic < 80)
NIDDM093	30	Female	Normal	220	360	180	148	157	42	Elevated (Systolic 120-129 Diastolic <80)
NIDDM094	58	Female	Obese	161	380	223	140	146	45	Normal (Systolic < 120 Diastolic < 80)
NIDDM095	67	Female	Over Weight	103	250	140	70	75	52	Normal (Systolic < 120 Diastolic < 80)
NIDDM096	46	Female	Over Weight	125	299	133	80	137	55	Normal (Systolic < 120 Diastolic < 80)
NIDDM097	58	Female	Obese	111	272	182	127	47	88	Normal (Systolic < 120 Diastolic < 80)
NIDDM098	40	Female	Over Weight	101	225	223	360	145	70	Normal (Systolic < 120 Diastolic < 80)
NIDDM099	58	Female	Normal	162	319	106	88	44	67	Normal (Systolic < 120 Diastolic < 80)
NIDDM100	39	Female	Obese	218	216	187	404	210	60	Normal (Systolic < 120 Diastolic < 80)
NIDDM101	62	Male	Over Weight	129	113	179	83	78	32	Elevated (Systolic 120-129 Diastolic <80)
NIDDM102	29	Female	Over Weight	237	131	112	330	80	45	Normal (Systolic < 120 Diastolic < 80)
NIDDM103	47	Male	Normal	222	139	181	400	107	41	Normal (Systolic < 120 Diastolic < 80)
NIDDM104	62	Male	Over Weight	93	221	199	310	48	51	Normal (Systolic < 120 Diastolic < 80)
NIDDM105	58	Male	Normal	218	334	111	300	109	42	Normal (Systolic < 120 Diastolic < 80)
NIDDM106	50	Male	Over Weight	124	475	109	120	80	54	Normal (Systolic < 120 Diastolic < 80)
NIDDM107	46	Female	Obese	263	300	200	378	121	36	Normal (Systolic < 120 Diastolic < 80)
NIDDM108	59	Female	Normal	102	170	123	80	78	30	Hypertension Stage 2 (Systolic >140 Diastolic >90)
NIDDM109	49	Male	Obese	96	97	100	395	140	52	Normal (Systolic < 120 Diastolic < 80)
NIDDM110	61	Female	Over Weight	125	174	212	99	96	65	Hypertension Stage 2 (Systolic >140 Diastolic >90)
NIDDM111	45	Male	Normal	132	328	214	110	94	35	Hypertension Stage 2 (Systolic >140 Diastolic >90)
NIDDM112	45	Female	Normal	140	403	109	340	140	37	Normal (Systolic < 120 Diastolic < 80)
NIDDM113	46	Female	Obese	150	254	126	367	184	38	Normal (Systolic < 120 Diastolic < 80)
NIDDM114	39	Female	Normal	124	523	132	400	123	41	Normal (Systolic < 120 Diastolic < 80)
NIDDM115	60	Male	Normal	297	256	186	467	120	42	Normal (Systolic < 120 Diastolic < 80)
NIDDM116	57	Male	Over Weight	110	247	223	34	145	38	Elevated (Systolic 120-129 Diastolic <80)
NIDDM117	44	Female	Over Weight	90	396	189	198	100	39	Normal (Systolic < 120 Diastolic < 80)
NIDDM118	46	Female	Normal	100	532	182	433	78	42	Normal (Systolic < 120 Diastolic < 80)
NIDDM119	56	Female	Normal	238	217	212	386	89	41	Normal (Systolic < 120 Diastolic < 80)
NIDDM120	46	Male	Normal	102	289	110	70	135	56	Normal (Systolic < 120 Diastolic < 80)
NIDDM121	47	Female	Over Weight	156	580	209	390	89	45	Normal (Systolic < 120 Diastolic < 80)
NIDDM122	39	Female	Over Weight	284	300	190	343	138	39	Normal (Systolic < 120 Diastolic < 80)
NIDDM123	39	Female	Over Weight	236	116	136	54	73	39	Elevated (Systolic 120-129 Diastolic <80)
NIDDM124	53	Female	Over Weight	125	521	130	50	68	30	Normal (Systolic < 120 Diastolic < 80)
NIDDM125	49	Female	Over Weight	188	236	180	60	90	42	Normal (Systolic < 120 Diastolic < 80)
NIDDM126	51	Female	Normal	349	365	213	378	135	39	Hypertension Stage 2 (Systolic >140 Diastolic >90)
NIDDM127	39	Female	Over Weight	143	218	156	102	38	40	Normal (Systolic < 120 Diastolic < 80)
NIDDM128	60	Male	Over Weight	200	310	200	215	96	40	Normal (Systolic < 120 Diastolic < 80)
NIDDM129	50	Female	Normal	199	244	175	24	145	39	Normal (Systolic < 120 Diastolic < 80)
NIDDM130	61	Female	Normal	145	214	184	300	135	45	Normal (Systolic < 120 Diastolic < 80)
NIDDM131	62	Female	Over Weight	100	172	178	56	98	33	Normal (Systolic < 120 Diastolic < 80)
NIDDM132	44	Male	Obese	307	172	180	45	70	56	Normal (Systolic < 120 Diastolic < 80)
NIDDM133	61	Female	Over Weight	125	225	207	80	79	48	Normal (Systolic < 120 Diastolic < 80)
NIDDM134	62	Female	Obese	100	124	200	118	54	38	Elevated (Systolic 120-129 Diastolic <80)
NIDDM135	57	Female	Over Weight	118	430	156	135	50	46	Elevated (Systolic 120-129 Diastolic <80)
NIDDM136	50	Female	Over Weight	116	58	107	378	120	45	Normal (Systolic < 120 Diastolic < 80)
NIDDM137	56	Male	Over Weight	98	124	101	56	135	90	Normal (Systolic < 120 Diastolic < 80)
NIDDM138	51	Female	Over Weight	93	180	182	204	52	39	Hypertension Stage 2 (Systolic >140 Diastolic >90)
NIDDM139	55	Male	Over Weight	125	218	100	200	75	63	Normal (Systolic < 120 Diastolic < 80)
NIDDM140	53	Female	Obese	236	217	199	300	87	52	Normal (Systolic < 120 Diastolic < 80)
NIDDM141	60	Female	Over Weight	135	225	201	90	98	48	Hypertension Stage 2 (Systolic >140 Diastolic >90)
NIDDM142	43	Female	Normal	190	215	210	190	150	36	Normal (Systolic < 120 Diastolic < 80)
NIDDM143	58	Female	Normal	343	118	187	167	95	34	Normal (Systolic < 120 Diastolic < 80)
NIDDM144	51	Female	Over Weight	120	116	185	180	192	34	Normal (Systolic < 120 Diastolic < 80)
NIDDM145	47	Male	Normal	81	223	182	105	123	36	Normal (Systolic < 120 Diastolic < 80)
NIDDM146	40	Female	Over Weight	186	224	112	360	190	74	Normal (Systolic < 120 Diastolic < 80)
NIDDM147	39	Female	Obese	146	169	213	400	210	66	Normal (Systolic < 120 Diastolic < 80)
NIDDM148	39	Male	Obese	300	280	215	390	67	22	Normal (Systolic < 120 Diastolic < 80)
NIDDM149	60	Male	Over Weight	205	186	210	136	58	24	Elevated (Systolic 120-129 Diastolic <80)
NIDDM150	58	Female	Over Weight	200	218	212	120	76	19	Hypertension Stage 2 (Systolic >140 Diastolic >90)
NIDDM151	44	Female	Over Weight	322	216	204	150	48	16	Normal (Systolic < 120 Diastolic < 80)
NIDDM152	61	Female	Normal	146	124	120	170	67	36	Normal (Systolic < 120 Diastolic < 80)
NIDDM153	52	Male	Over Weight	86	125	215	300	80	75	Normal (Systolic < 120 Diastolic < 80)
NIDDM154	54	Female	Obese	87	213	212	360	89	37	Normal (Systolic < 120 Diastolic < 80)
NIDDM155	56	Male	Obese	199	253	210	410	65	56	Normal (Systolic < 120 Diastolic < 80)
NIDDM156	40	Female	Obese	168	216	114	130	39	33	Normal (Systolic < 120 Diastolic < 80)
NIDDM157	53	Male	Over Weight	146	234	108	237	96	37	Elevated (Systolic 120-129 Diastolic <80)
NIDDM158	54	Female	Normal	159	224	215	210	58	60	Normal (Systolic < 120 Diastolic < 80)
NIDDM159	62	Female	Obese	120	250	208	204	98	73	Hypertension Stage 2 (Systolic >140 Diastolic >90)
NIDDM160	40	Female	Obese	277	312	146	333	91	45	Normal (Systolic < 120 Diastolic < 80)
NIDDM161	50	Female	Obese	199	216	151	182	132	39	Normal (Systolic < 120 Diastolic < 80)
NIDDM162	43	Female	Over Weight	21	210	170	356	123	56	Normal (Systolic < 120 Diastolic < 80)
NIDDM163	46	Female	Over Weight	356	210	189	97	131	39	Normal (Systolic < 120 Diastolic < 80)
NIDDM164	61	Female	Obese	144	224	167	120	156	122	Elevated (Systolic 120-129 Diastolic <80)
NIDDM165	59	Female	Obese	213	139	170	135	39	67	Hypertension Stage 2 (Systolic >140 Diastolic >90)

NIDDM166	62	Female	Over Weight	158	225	190	90	67	63	Normal (Systolic < 120 Diastolic < 80)
NIDDM167	53	Female	Over Weight	159	216	145	120	80	48	Normal (Systolic < 120 Diastolic < 80)
NIDDM168	54	Female	Over Weight	258	147	208	174	135	38	Normal (Systolic < 120 Diastolic < 80)
NIDDM169	57	Female	Over Weight	180	210	203	160	87	43	Normal (Systolic < 120 Diastolic < 80)
NIDDM170	49	Female	Over Weight	407	375	178	120	70	54	Normal (Systolic < 120 Diastolic < 80)
NIDDM171	58	Male	Over Weight	108	160	190	115	50	43	Elevated (Systolic 120-129 Diastolic <80)
NIDDM172	56	Female	Over Weight	80	395	182	89	38	60	Normal (Systolic < 120 Diastolic < 80)
NIDDM173	54	Female	Over Weight	170	167	156	210	120	61	Normal (Systolic < 120 Diastolic < 80)
NIDDM174	39	Female	Obese	251	580	174	165	84	42	Normal (Systolic < 120 Diastolic < 80)
NIDDM175	61	Female	Over Weight	180	217	222	204	178	12	Hypertension Stage 2 (Systolic >140 Diastolic >90)
NIDDM176	46	Female	Obese	213	218	176	200	50	32	Normal (Systolic < 120 Diastolic < 80)
NIDDM177	39	Male	Over Weight	145	573	209	324	78	45	Normal (Systolic < 120 Diastolic < 80)
NIDDM178	58	Male	Over Weight	65	213	112	126	70	47	Normal (Systolic < 120 Diastolic < 80)
NIDDM179	42	Female	Over Weight	90	225	143	321	108	37	Normal (Systolic < 120 Diastolic < 80)
NIDDM180	42	Male	Over Weight	196	526	205	81	157	72	Elevated (Systolic 120-129 Diastolic <80)
NIDDM181	39	Female	Obese	189	221	156	190	150	45	Normal (Systolic < 120 Diastolic < 80)
NIDDM182	47	Female	Obese	127	216	200	79	44	37	Normal (Systolic < 120 Diastolic < 80)
NIDDM183	45	Female	Over Weight	253	376	190	114	73	35	Hypertension Stage 2 (Systolic >140 Diastolic >90)
NIDDM184	50	Female	Over Weight	218	395	178	145	67	43	Normal (Systolic < 120 Diastolic < 80)
NIDDM185	62	Female	Over Weight	121	218	180	312	73	40	Elevated (Systolic 120-129 Diastolic <80)
NIDDM186	55	Female	Over Weight	261	147	173	267	89	37	Hypertension Stage 2 (Systolic >140 Diastolic >90)
NIDDM187	60	Female	Over Weight	123	221	135	220	40	72	Normal (Systolic < 120 Diastolic < 80)
NIDDM188	43	Male	Over Weight	334	225	145	235	65	38	Normal (Systolic < 120 Diastolic < 80)
NIDDM189	52	Female	Over Weight	265	215	190	216	63	29	Hypertension Stage 2 (Systolic >140 Diastolic >90)
NIDDM190	59	Female	Over Weight	199	217	182	278	65	30	Hypertension Stage 2 (Systolic >140 Diastolic >90)
NIDDM191	43	Female	Over Weight	70	521	190	310	90	35	Normal (Systolic < 120 Diastolic < 80)
NIDDM192	49	Female	Over Weight	69	142	233	306	65	41	Normal (Systolic < 120 Diastolic < 80)
NIDDM193	48	Male	Over Weight	294	218	225	290	54	60	Normal (Systolic < 120 Diastolic < 80)
NIDDM194	39	Male	Over Weight	149	497	182	370	156	50	Normal (Systolic < 120 Diastolic < 80)
NIDDM195	37	Female	Obese	165	207	179	204	143	65	Normal (Systolic < 120 Diastolic < 80)
NIDDM196	36	Male	Over Weight	153	379	221	206	77	42	Elevated (Systolic 120-129 Diastolic <80)
NIDDM197	32	Female	Obese	98	273	182	190	65	102	Hypertension Stage 2 (Systolic >140 Diastolic >90)
NIDDM198	24	Female	Obese	331	214	222	185	90	69	Normal (Systolic < 120 Diastolic < 80)
NIDDM199	44	Female	Obese	213	248	219	110	95	48	Normal (Systolic < 120 Diastolic < 80)
NIDDM200	30	Male	Obese	125	112	200	58	107	58	Elevated (Systolic 120-129 Diastolic <80)
NIDDM201	29	Female	Obese	250	123	210	120	130	48	Elevated (Systolic 120-129 Diastolic <80)
NIDDM202	27	Male	Obese	189	274	180	80	170	49	Normal (Systolic < 120 Diastolic < 80)
NIDDM203	25	Male	Obese	250	110	182	190	125	31	Normal (Systolic < 120 Diastolic < 80)
NIDDM204	24	Female	Obese	188	481	180	49	130	43	Normal (Systolic < 120 Diastolic < 80)
NIDDM205	32	Female	Obese	188	217	182	398	154	50	Normal (Systolic < 120 Diastolic < 80)
NIDDM206	71	Female	Obese	198	532	200	350	135	65	Hypertension Stage 2 (Systolic >140 Diastolic >90)
NIDDM207	34	Male	Obese	165	202	210	170	150	42	Hypertension Stage 2 (Systolic >140 Diastolic >90)
NIDDM208	67	Female	Obese	156	512	221	185	145	45	Normal (Systolic < 120 Diastolic < 80)
NIDDM209	65	Female	Obese	398	227	330	120	80	42	Normal (Systolic < 120 Diastolic < 80)
NIDDM210	74	Female	Obese	69	116	153	506	90	11	Normal (Systolic < 120 Diastolic < 80)
NIDDM211	24	Male	Over Weight	153	460	320	379	95	62	Normal (Systolic < 120 Diastolic < 80)
NIDDM212	22	Female	Obese	156	225	181	90	100	54	Elevated (Systolic 120-129 Diastolic <80)
NIDDM213	43	Female	Obese	188	216	110	87	107	34	Hypertension Stage 2 (Systolic >140 Diastolic >90)
NIDDM214	61	Male	Obese	253	215	92	56	123	46	Elevated (Systolic 120-129 Diastolic <80)
NIDDM215	60	Male	Obese	246	217	220	180	156	30	Hypertension Stage 2 (Systolic >140 Diastolic >90)
NIDDM216	67	Female	Obese	336	136	360	149	150	66	Normal (Systolic < 120 Diastolic < 80)
NIDDM217	39	Male	Obese	186	219	160	141	123	36	Normal (Systolic < 120 Diastolic < 80)
NIDDM218	40	Female	Obese	265	521	112	390	134	51	Normal (Systolic < 120 Diastolic < 80)
NIDDM219	78	Female	Obese	253	133	129	380	156	43	Hypertension Stage 2 (Systolic >140 Diastolic >90)
NIDDM220	61	Female	Obese	169	268	185	387	95	48	Hypertension Stage 2 (Systolic >140 Diastolic >90)
NIDDM221	47	Female	Obese	187	216	86	310	90	29	Elevated (Systolic 120-129 Diastolic <80)
NIDDM222	69	Female	Obese	259	151	130	300	94	38	Normal (Systolic < 120 Diastolic < 80)
NIDDM223	45	Female	Over Weight	228	238	110	350	67	36	Hypertension Stage 2 (Systolic >140 Diastolic >90)
NIDDM224	42	Female	Over Weight	208	152	320	379	80	32	Hypertension Stage 2 (Systolic >140 Diastolic >90)
NIDDM225	61	Female	Obese	109	390	100	54	122	41	Hypertension Stage 2 (Systolic >140 Diastolic >90)
NIDDM226	41	Female	Obese	190	137	133	398	67	34	Normal (Systolic < 120 Diastolic < 80)
NIDDM227	69	Female	Obese	200	112	98	109	150	42	Hypertension Stage 2 (Systolic >140 Diastolic >90)
NIDDM228	58	Female	Over Weight	480	421	231	200	84	52	Hypertension Stage 2 (Systolic >140 Diastolic >90)
NIDDM229	41	Male	Over Weight	205	317	181	180	95	56	Hypertension Stage 2 (Systolic >140 Diastolic >90)
NIDDM230	43	Female	Obese	186	531	105	389	69	49	Normal (Systolic < 120 Diastolic < 80)
NIDDM231	24	Female	Over Weight	128	232	200	145	59	51	Normal (Systolic < 120 Diastolic < 80)
NIDDM232	23	Male	Over Weight	102	241	210	380	78	42	Normal (Systolic < 120 Diastolic < 80)
NIDDM233	40	Female	Obese	420	216	178	75	71	39	Normal (Systolic < 120 Diastolic < 80)
NIDDM234	39	Female	Obese	196	365	182	190	76	36	Elevated (Systolic 120-129 Diastolic <80)
NIDDM235	24	Female	Obese	270	218	195	180	98	29	Hypertension Stage 2 (Systolic >140 Diastolic >90)
NIDDM236	23	Female	Over Weight	201	182	186	145	95	42	Hypertension Stage 2 (Systolic >140 Diastolic >90)
NIDDM237	26	Male	Obese	450	185	185	95	93	55	Elevated (Systolic 120-129 Diastolic <80)
NIDDM238	59	Male	Over Weight	347	217	182	56	79	49	Hypertension Stage 2 (Systolic >140 Diastolic >90)
NIDDM239	72	Male	Over Weight	156	91	179	50	67	42	Hypertension Stage 2 (Systolic >140 Diastolic >90)
NIDDM240	74	Female	Over Weight	119	221	180	300	56	37	Normal (Systolic < 120 Diastolic < 80)
NIDDM241	61	Female	Obese	490	524	178	45	50	11	Elevated (Systolic 120-129 Diastolic <80)
NIDDM242	45	Male	Obese	108	489	190	78	87	60	Hypertension Stage 2 (Systolic >140 Diastolic >90)
NIDDM243	53	Female	Obese	158	469	229	430	83	36	Normal (Systolic < 120 Diastolic < 80)
NIDDM244	58	Male	Obese	188	215	156	123	98	47	Normal (Systolic < 120 Diastolic < 80)
NIDDM245	60	Male	Obese	299	211	350	169	123	43	Hypertension Stage 2 (Systolic >140 Diastolic >90)
NIDDM246	61	Female	Obese	154	216	140	190	45	27	Normal (Systolic < 120 Diastolic < 80)
NIDDM247	56	Female	Obese	369	329	270	69	87	45	Hypertension Stage 2 (Systolic >140 Diastolic >90)
NIDDM248	66	Female	Obese	146	584	180	338	80	39	Normal (Systolic < 120 Diastolic < 80)
NIDDM249	64	Female	Obese	186	165	182	105	78	63	Hypertension Stage 2 (Systolic >140 Diastolic >90)

NIDDM250	70	Male	Over Weight	156	434	189	340	85	11	Normal (Systolic < 120 Diastolic < 80)
NIDDM251	43	Female	Obese	295	223	178	280	83	61	Hypertension Stage 2 (Systolic >140 Diastolic >90)
NIDDM252	70	Female	Normal	159	234	188	60	67	73	Normal (Systolic < 120 Diastolic < 80)
NIDDM253	73	Female	Obese	224	110	184	56	94	48	Normal (Systolic < 120 Diastolic < 80)
NIDDM254	76	Female	Obese	164	333	182	302	70	51	Normal (Systolic < 120 Diastolic < 80)
NIDDM255	66	Female	Obese	163	118	231	34	75	56	Normal (Systolic < 120 Diastolic < 80)
NIDDM256	62	Male	Obese	259	150	131	137	65	39	Hypertension Stage 2 (Systolic >140 Diastolic >90)
NIDDM257	44	Female	Obese	135	96	236	30	78	70	Hypertension Stage 2 (Systolic >140 Diastolic >90)
NIDDM258	60	Male	Obese	72	215	185	177	110	40	Hypertension Stage 2 (Systolic >140 Diastolic >90)
NIDDM259	66	Female	Obese	176	90	156	323	89	56	Normal (Systolic < 120 Diastolic < 80)
NIDDM260	40	Male	Obese	314	423	228	123	84	40	Normal (Systolic < 120 Diastolic < 80)
NIDDM261	62	Male	Obese	276	348	120	90	82	35	Normal (Systolic < 120 Diastolic < 80)
NIDDM262	39	Male	Over Weight	156	124	181	56	84	56	Normal (Systolic < 120 Diastolic < 80)
NIDDM263	68	Female	Obese	365	119	183	398	78	43	Hypertension Stage 2 (Systolic >140 Diastolic >90)
NIDDM264	46	Female	Obese	134	214	179	59	90	37	Hypertension Stage 2 (Systolic >140 Diastolic >90)
NIDDM265	41	Female	Over Weight	184	241	224	61	56	16	Normal (Systolic < 120 Diastolic < 80)
NIDDM266	61	Female	Obese	206	116	229	80	74	47	Hypertension Stage 2 (Systolic >140 Diastolic >90)
NIDDM267	60	Female	Over Weight	156	113	222	70	56	20	Normal (Systolic < 120 Diastolic < 80)
NIDDM268	65	Male	Obese	358	225	223	90	78	48	Hypertension Stage 2 (Systolic >140 Diastolic >90)
NIDDM269	69	Female	Obese	246	221	233	100	68	62	Hypertension Stage 2 (Systolic >140 Diastolic >90)
NIDDM270	74	Female	Obese	158	209	178	135	107	44	Hypertension Stage 2 (Systolic >140 Diastolic >90)
NIDDM271	68	Male	Obese	246	222	201	165	97	42	Hypertension Stage 2 (Systolic >140 Diastolic >90)
NIDDM272	44	Female	Obese	256	242	225	204	105	56	Hypertension Stage 2 (Systolic >140 Diastolic >90)
NIDDM273	69	Female	Obese	213	428	146	207	123	78	Normal (Systolic < 120 Diastolic < 80)
NIDDM274	68	Male	Obese	425	415	163	398	134	90	Elevated (Systolic 120-129 Diastolic <80)
NIDDM275	70	Female	Obese	145	242	164	116	106	35	Hypertension Stage 2 (Systolic >140 Diastolic >90)
NIDDM276	59	Female	Obese	144	396	231	90	23	192	Hypertension Stage 2 (Systolic >140 Diastolic >90)
NIDDM277	42	Female	Over Weight	132	120	230	198	145	55	Normal (Systolic < 120 Diastolic < 80)
NIDDM278	67	Male	Obese	117	150	123	128	57	31	Normal (Systolic < 120 Diastolic < 80)
NIDDM279	26	Female	Over Weight	125	112	221	201	67	45	Normal (Systolic < 120 Diastolic < 80)
NIDDM280	68	Female	Obese	100	180	225	300	78	67	Hypertension Stage 2 (Systolic >140 Diastolic >90)
NIDDM281	65	Female	Obese	460	250	80	310	51	87	Normal (Systolic < 120 Diastolic < 80)
NIDDM282	39	Female	Obese	126	146	231	340	60	57	Normal (Systolic < 120 Diastolic < 80)
NIDDM283	57	Female	Over Weight	120	218	235	327	67	88	Elevated (Systolic 120-129 Diastolic <80)
NIDDM284	58	Female	Over Weight	300	221	204	250	65	43	Hypertension Stage 2 (Systolic >140 Diastolic >90)
NIDDM285	61	Male	Obese	98	214	182	119	51	59	Normal (Systolic < 120 Diastolic < 80)
NIDDM286	28	Female	Obese	322	154	155	200	57	76	Hypertension Stage 2 (Systolic >140 Diastolic >90)
NIDDM287	26	Female	Normal	89	243	166	250	76	40	Normal (Systolic < 120 Diastolic < 80)
NIDDM288	46	Female	Obese	269	415	135	259	123	11	Normal (Systolic < 120 Diastolic < 80)
NIDDM289	60	Female	Obese	199	118	185	321	176	13	Hypertension Stage 2 (Systolic >140 Diastolic >90)
NIDDM290	61	Male	Over Weight	85	188	182	200	195	53	Hypertension Stage 2 (Systolic >140 Diastolic >90)
NIDDM291	30	Female	Over Weight	235	113	185	237	78	37	Hypertension Stage 2 (Systolic >140 Diastolic >90)
NIDDM292	33	Female	Over Weight	326	133	180	190	180	50	Hypertension Stage 2 (Systolic >140 Diastolic >90)
NIDDM293	35	Male	Obese	201	118	179	187	89	64	Normal (Systolic < 120 Diastolic < 80)
NIDDM294	29	Female	Over Weight	406	268	90	178	201	54	Elevated (Systolic 120-129 Diastolic <80)
NIDDM295	44	Female	Obese	228	345	149	168	86	53	Normal (Systolic < 120 Diastolic < 80)
NIDDM296	58	Male	Obese	308	457	143	180	130	40	Normal (Systolic < 120 Diastolic < 80)
NIDDM297	62	Male	Obese	192	92	256	178	76	14	Normal (Systolic < 120 Diastolic < 80)
NIDDM298	32	Female	Obese	186	74	190	195	71	75	Elevated (Systolic 120-129 Diastolic <80)
NIDDM299	72	Female	Obese	360	117	180	210	129	39	Normal (Systolic < 120 Diastolic < 80)
NIDDM300	57	Female	Normal	268	215	106	216	89	41	Normal (Systolic < 120 Diastolic < 80)
NIDDM301	34	Male	Over Weight	269	470	130	190	127	57	Normal (Systolic < 120 Diastolic < 80)
NIDDM302	30	Female	Over Weight	345	138	181	40	119	34	Hypertension Stage 2 (Systolic >140 Diastolic >90)
NIDDM303	72	Female	Obese	63	279	140	35	121	27	Normal (Systolic < 120 Diastolic < 80)
NIDDM304	40	Male	Over Weight	98	213	186	56	121	54	Normal (Systolic < 120 Diastolic < 80)
NIDDM305	43	Male	Obese	100	300	182	67	90	28	Hypertension Stage 2 (Systolic >140 Diastolic >90)
NIDDM306	77	Female	Normal	146	90	178	237	73	50	Normal (Systolic < 120 Diastolic < 80)
NIDDM307	68	Male	Normal	200	253	175	37	67	37	Normal (Systolic < 120 Diastolic < 80)
NIDDM308	40	Female	Obese	294	216	133	79	69	29	Normal (Systolic < 120 Diastolic < 80)
NIDDM309	71	Female	Over Weight	215	195	181	278	76	38	Normal (Systolic < 120 Diastolic < 80)
NIDDM310	60	Male	Obese	122	180	180	284	123	42	Normal (Systolic < 120 Diastolic < 80)
NIDDM311	42	Female	Obese	112	116	201	94	126	37	Normal (Systolic < 120 Diastolic < 80)
NIDDM312	60	Female	Obese	201	224	144	79	130	32	Normal (Systolic < 120 Diastolic < 80)
NIDDM313	72	Female	Obese	346	418	110	21	67	32	Hypertension Stage 2 (Systolic >140 Diastolic >90)
NIDDM314	67	Female	Over Weight	57	129	190	276	91	424	Normal (Systolic < 120 Diastolic < 80)
NIDDM315	28	Male	Obese	285	189	140	35	89	45	Hypertension Stage 2 (Systolic >140 Diastolic >90)
NIDDM316	43	Male	Obese	110	117	125	67	90	67	Normal (Systolic < 120 Diastolic < 80)
NIDDM317	26	Female	Obese	125	169	159	336	95	54	Normal (Systolic < 120 Diastolic < 80)
NIDDM318	60	Female	Over Weight	180	217	123	320	154	56	Normal (Systolic < 120 Diastolic < 80)
NIDDM319	39	Female	Normal	107	221	140	370	134	78	Normal (Systolic < 120 Diastolic < 80)
NIDDM320	46	Female	Obese	125	210	119	200	123	47	Elevated (Systolic 120-129 Diastolic <80)
NIDDM321	56	Female	Over Weight	301	253	140	56	112	42	Normal (Systolic < 120 Diastolic < 80)
NIDDM322	40	Female	Over Weight	253	412	145	45	106	40	Normal (Systolic < 120 Diastolic < 80)
NIDDM323	47	Male	Over Weight	96	216	350	51	107	74	Normal (Systolic < 120 Diastolic < 80)
NIDDM324	23	Female	Obese	69	274	160	87	74	37	Normal (Systolic < 120 Diastolic < 80)
NIDDM325	33	Male	Obese	213	395	180	310	56	40	Elevated (Systolic 120-129 Diastolic <80)
NIDDM326	43	Male	Obese	121	215	183	347	78	56	Normal (Systolic < 120 Diastolic < 80)
NIDDM327	59	Female	Obese	212	175	230	401	98	35	Normal (Systolic < 120 Diastolic < 80)
NIDDM328	41	Female	Over Weight	169	219	270	290	95	56	Normal (Systolic < 120 Diastolic < 80)
NIDDM329	40	Female	Obese	227	282	168	67	90	35	Normal (Systolic < 120 Diastolic < 80)
NIDDM330	53	Female	Over Weight	129	104	230	478	87	32	Hypertension Stage 2 (Systolic >140 Diastolic >90)
NIDDM331	57	Female	Obese	118	167	223	102	68	31	Normal (Systolic < 120 Diastolic < 80)
NIDDM332	53	Female	Obese	102	217	110	300	85	54	Normal (Systolic < 120 Diastolic < 80)
NIDDM333	53	Female	Obese	210	222	190	41	81	33	Normal (Systolic < 120 Diastolic < 80)

NIDDM334	69	Female	Obese	146	210	278	51	123	32	Elevated (Systolic 120-129 Diastolic <80)
NIDDM335	61	Female	Over Weight	270	113	227	267	110	50	Normal (Systolic < 120 Diastolic < 80)
NIDDM336	57	Female	Obese	110	315	289	390	86	43	Normal (Systolic < 120 Diastolic < 80)
NIDDM337	44	Female	Obese	345	215	223	189	87	37	Normal (Systolic < 120 Diastolic < 80)
NIDDM338	54	Female	Normal	490	199	178	190	90	35	Normal (Systolic < 120 Diastolic < 80)
NIDDM339	46	Male	Over Weight	110	148	122	168	135	62	Normal (Systolic < 120 Diastolic < 80)
NIDDM340	58	Female	Obese	220	430	181	410	125	45	Normal (Systolic < 120 Diastolic < 80)
NIDDM341	60	Female	Obese	249	121	182	81	108	69	Normal (Systolic < 120 Diastolic < 80)
NIDDM342	35	Female	Obese	109	278	180	198	122	23	Normal (Systolic < 120 Diastolic < 80)
NIDDM343	49	Female	Obese	456	195	234	213	93	62	Normal (Systolic < 120 Diastolic < 80)
NIDDM344	64	Male	Over Weight	76	221	227	456	139	65	Normal (Systolic < 120 Diastolic < 80)
NIDDM345	58	Male	Normal	415	337	171	159	123	20	Normal (Systolic < 120 Diastolic < 80)
NIDDM346	46	Female	Obese	103	293	227	155	109	50	Normal (Systolic < 120 Diastolic < 80)
NIDDM347	38	Female	Obese	169	218	229	180	96	15	Normal (Systolic < 120 Diastolic < 80)
NIDDM348	57	Female	Obese	246	114	180	294	140	49	Normal (Systolic < 120 Diastolic < 80)
NIDDM349	70	Female	Obese	99	124	300	150	112	31	Normal (Systolic < 120 Diastolic < 80)
NIDDM350	71	Female	Obese	365	222	182	390	132	58	Normal (Systolic < 120 Diastolic < 80)
NIDDM351	68	Female	Over Weight	143	226	181	310	135	77	Normal (Systolic < 120 Diastolic < 80)
NIDDM352	34	Female	Obese	156	448	180	178	96	25	Normal (Systolic < 120 Diastolic < 80)
NIDDM353	39	Male	Obese	158	290	189	87	90	17	Elevated (Systolic 120-129 Diastolic <80)
NIDDM354	56	Female	Over Weight	215	537	236	78	94	67	Normal (Systolic < 120 Diastolic < 80)
NIDDM355	67	Female	Obese	112	82	204	31	89	18	Normal (Systolic < 120 Diastolic < 80)
NIDDM356	59	Female	Obese	106	222	223	345	76	76	Hypertension Stage 2 (Systolic >140 Diastolic >90)
NIDDM357	37	Female	Normal	285	165	228	422	74	45	Normal (Systolic < 120 Diastolic < 80)
NIDDM358	46	Female	Over Weight	128	225	112	76	65	87	Hypertension Stage 2 (Systolic >140 Diastolic >90)
NIDDM359	65	Female	Over Weight	88	213	109	101	69	57	Elevated (Systolic 120-129 Diastolic <80)
NIDDM360	40	Male	Obese	124	305	230	182	117	37	Hypertension Stage 2 (Systolic >140 Diastolic >90)
NIDDM361	48	Male	Over Weight	186	155	145	156	68	26	Hypertension Stage 2 (Systolic >140 Diastolic >90)
NIDDM362	62	Female	Obese	94	490	223	170	56	29	Hypertension Stage 2 (Systolic >140 Diastolic >90)
NIDDM363	44	Male	Obese	215	205	226	189	78	26	Hypertension Stage 2 (Systolic >140 Diastolic >90)
NIDDM364	40	Male	Normal	86	404	241	67	98	64	Elevated (Systolic 120-129 Diastolic <80)
NIDDM365	62	Female	Normal	76	117	162	326	95	60	Elevated (Systolic 120-129 Diastolic <80)
NIDDM366	56	Female	Obese	135	207	165	409	54	72	Hypertension Stage 2 (Systolic >140 Diastolic >90)
NIDDM367	39	Female	Over Weight	115	89	226	46	46	13	Elevated (Systolic 120-129 Diastolic <80)
NIDDM368	45	Female	Obese	235	184	178	290	95	49	Hypertension Stage 2 (Systolic >140 Diastolic >90)
NIDDM369	43	Female	Obese	114	129	263	692	85	40	Elevated (Systolic 120-129 Diastolic <80)
NIDDM370	48	Male	Obese	263	385	182	200	84	69	Hypertension Stage 2 (Systolic >140 Diastolic >90)
NIDDM371	47	Male	Over Weight	140	213	181	210	75	26	Hypertension Stage 2 (Systolic >140 Diastolic >90)
NIDDM372	67	Female	Over Weight	130	218	184	267	70	76	Hypertension Stage 2 (Systolic >140 Diastolic >90)
NIDDM373	46	Male	Obese	155	216	179	249	90	41	Hypertension Stage 2 (Systolic >140 Diastolic >90)
NIDDM374	57	Female	Over Weight	260	321	181	228	70	72	Hypertension Stage 2 (Systolic >140 Diastolic >90)
NIDDM375	60	Male	Obese	70	301	187	310	80	26	Elevated (Systolic 120-129 Diastolic <80)
NIDDM376	47	Male	Obese	194	500	180	422	96	8	Hypertension Stage 2 (Systolic >140 Diastolic >90)
NIDDM377	70	Male	Obese	176	475	300	95	75	66	Hypertension Stage 2 (Systolic >140 Diastolic >90)
NIDDM378	42	Male	Obese	88	217	270	110	84	20	Hypertension Stage 2 (Systolic >140 Diastolic >90)
NIDDM379	68	Male	Obese	249	216	275	94	61	67	Elevated (Systolic 120-129 Diastolic <80)
NIDDM380	65	Male	Obese	375	222	280	61	35	77	Hypertension Stage 2 (Systolic >140 Diastolic >90)
NIDDM381	70	Male	Obese	175	110	280	298	78	54	Hypertension Stage 2 (Systolic >140 Diastolic >90)
NIDDM382	68	Male	Over Weight	185	158	340	368	70	23	Hypertension Stage 2 (Systolic >140 Diastolic >90)
NIDDM383	42	Female	Obese	188	116	171	380	131	73	Hypertension Stage 2 (Systolic >140 Diastolic >90)
NIDDM384	46	Male	Obese	200	434	241	80	91	42	Hypertension Stage 2 (Systolic >140 Diastolic >90)
NIDDM385	61	Female	Obese	85	129	230	95	98	59	Hypertension Stage 2 (Systolic >140 Diastolic >90)
NIDDM386	44	Female	Obese	70	466	156	120	95	64	Hypertension Stage 2 (Systolic >140 Diastolic >90)
NIDDM387	68	Female	Obese	225	283	160	78	123	45	Hypertension Stage 2 (Systolic >140 Diastolic >90)
NIDDM388	65	Male	Obese	274	334	200	200	111	42	Hypertension Stage 2 (Systolic >140 Diastolic >90)
NIDDM389	45	Female	Over Weight	95	164	223	490	65	56	Hypertension Stage 2 (Systolic >140 Diastolic >90)
NIDDM390	43	Male	Obese	87	150	174	132	118	47	Elevated (Systolic 120-129 Diastolic <80)
NIDDM391	61	Female	Obese	216	213	179	156	76	78	Hypertension Stage 2 (Systolic >140 Diastolic >90)
NIDDM392	60	Male	Over Weight	185	105	222	313	119	40	Hypertension Stage 2 (Systolic >140 Diastolic >90)
NIDDM393	54	Male	Over Weight	246	349	182	35	50	65	Hypertension Stage 2 (Systolic >140 Diastolic >90)
NIDDM394	44	Female	Obese	175	212	223	29	89	50	Hypertension Stage 2 (Systolic >140 Diastolic >90)
NIDDM395	47	Female	Obese	216	166	226	310	100	45	Hypertension Stage 2 (Systolic >140 Diastolic >90)
NIDDM396	35	Female	Over Weight	77	136	227	103	82	32	Hypertension Stage 2 (Systolic >140 Diastolic >90)
NIDDM397	69	Female	Over Weight	99	342	165	90	81	33	Hypertension Stage 2 (Systolic >140 Diastolic >90)
NIDDM398	74	Female	Obese	153	211	167	81	76	7	Hypertension Stage 2 (Systolic >140 Diastolic >90)
NIDDM399	70	Male	Over Weight	414	157	177	85	70	49	Hypertension Stage 2 (Systolic >140 Diastolic >90)
NIDDM400	38	Female	Obese	143	221	228	402	132	8	Hypertension Stage 2 (Systolic >140 Diastolic >90)
NIDDM401	32	Female	Obese	89	162	200	23	97	13	Elevated (Systolic 120-129 Diastolic <80)
NIDDM402	47	Male	Obese	123	216	190	54	91	18	Hypertension Stage 2 (Systolic >140 Diastolic >90)
NIDDM403	60	Female	Over Weight	58	345	265	62	49	31	Hypertension Stage 2 (Systolic >140 Diastolic >90)
NIDDM404	57	Female	Over Weight	156	40	234	12	120	78	Hypertension Stage 2 (Systolic >140 Diastolic >90)
NIDDM405	30	Female	Over Weight	253	230	231	1.6	3.2	0.9	Elevated (Systolic 120-129 Diastolic <80)
NIDDM406	57	Male	Obese	134	417	201	92	80	64	Hypertension Stage 2 (Systolic >140 Diastolic >90)
NIDDM407	55	Male	Obese	146	222	222	96	34	34	Hypertension Stage 2 (Systolic >140 Diastolic >90)
NIDDM408	35	Female	Obese	175	215	108	378	88	67	Hypertension Stage 2 (Systolic >140 Diastolic >90)
NIDDM409	34	Female	Over Weight	98	125	212	335	116	79	Hypertension Stage 2 (Systolic >140 Diastolic >90)
NIDDM410	41	Male	Over Weight	186	222	171	64	104	55	Elevated (Systolic 120-129 Diastolic <80)
NIDDM411	56	Female	Obese	146	213	177	318	109	59	Hypertension Stage 2 (Systolic >140 Diastolic >90)
NIDDM412	32	Female	Over Weight	215	216	204	94	108	10	Hypertension Stage 2 (Systolic >140 Diastolic >90)
NIDDM413	28	Female	Obese	231	214	209	341	27	23	Hypertension Stage 2 (Systolic >140 Diastolic >90)
NIDDM414	53	Male	Over Weight	134	127	151	50	91	78	Hypertension Stage 2 (Systolic >140 Diastolic >90)
NIDDM415	48	Female	Normal	157	210	196	140	108	76	Hypertension Stage 2 (Systolic >140 Diastolic >90)
NIDDM416	42	Female	Over Weight	97	216	208	112	73	65	Hypertension Stage 2 (Systolic >140 Diastolic >90)
NIDDM417	32	Male	Over Weight	241	118	207	70	100	66	Hypertension Stage 2 (Systolic >140 Diastolic >90)

NIDDM418	33	Female	Over Weight	150	458	175	65	125	65	Hypertension Stage 2 (Systolic >140 Diastolic >90)
NIDDM419	43	Female	Over Weight	186	216	240	367	109	76	Hypertension Stage 2 (Systolic >140 Diastolic >90)
NIDDM420	40	Female	Over Weight	194	210	197	149	117	64	Hypertension Stage 2 (Systolic >140 Diastolic >90)
NIDDM421	62	Male	Over Weight	176	220	205	94	88	72	Elevated (Systolic 120-129 Diastolic <80)
NIDDM422	40	Male	Over Weight	120	218	196	123	96	56	Hypertension Stage 2 (Systolic >140 Diastolic >90)
NIDDM423	60	Female	Over Weight	280	215	201	53	105	21	Hypertension Stage 2 (Systolic >140 Diastolic >90)
NIDDM424	28	Female	Over Weight	65	225	183	415	96	11	Hypertension Stage 2 (Systolic >140 Diastolic >90)
NIDDM425	29	Male	Over Weight	130	219	200	397	74	64	Elevated (Systolic 120-129 Diastolic <80)
NIDDM426	43	Female	Over Weight	125	125	191	82	121	17	Hypertension Stage 2 (Systolic >140 Diastolic >90)
NIDDM427	45	Male	Over Weight	90	118	230	52	67	72	Hypertension Stage 2 (Systolic >140 Diastolic >90)
NIDDM428	62	Female	Over Weight	270	217	203	67	121	29	Hypertension Stage 2 (Systolic >140 Diastolic >90)
NIDDM429	35	Female	Normal	245	214	176	380	122	15	Elevated (Systolic 120-129 Diastolic <80)
NIDDM430	72	Male	Over Weight	400	210	168	65	45	73	Hypertension Stage 2 (Systolic >140 Diastolic >90)
NIDDM431	60	Female	Over Weight	410	220	192	145	41	35	Hypertension Stage 2 (Systolic >140 Diastolic >90)
NIDDM432	43	Female	Obese	300	134	202	72	96	11	Hypertension Stage 2 (Systolic >140 Diastolic >90)
NIDDM433	35	Female	Over Weight	60	218	175	325	33	51	Elevated (Systolic 120-129 Diastolic <80)
NIDDM434	75	Female	Normal	175	213	179	350	70	29	Hypertension Stage 2 (Systolic >140 Diastolic >90)
NIDDM435	65	Male	Over Weight	260	210	170	345	67	73	Hypertension Stage 2 (Systolic >140 Diastolic >90)
NIDDM436	58	Female	Over Weight	400	218	167	304	78	32	Elevated (Systolic 120-129 Diastolic <80)
NIDDM437	35	Female	Obese	215	221	179	426	80	57	Hypertension Stage 2 (Systolic >140 Diastolic >90)
NIDDM438	41	Female	Obese	146	220	203	356	54	15	Hypertension Stage 2 (Systolic >140 Diastolic >90)
NIDDM439	62	Male	Obese	158	216	191	284	49	31	Hypertension Stage 2 (Systolic >140 Diastolic >90)
NIDDM440	49	Male	Over Weight	146	215	172	300	160	68	Hypertension Stage 2 (Systolic >140 Diastolic >90)
NIDDM441	78	Male	Normal	125	217	188	380	137	16	Hypertension Stage 2 (Systolic >140 Diastolic >90)
NIDDM442	72	Female	Over Weight	88	213	172	307	84	73	Elevated (Systolic 120-129 Diastolic <80)
NIDDM443	46	Female	Obese	270	210	185	75	78	51	Hypertension Stage 2 (Systolic >140 Diastolic >90)
NIDDM444	68	Female	Normal	100	210	195	51	170	36	Hypertension Stage 2 (Systolic >140 Diastolic >90)
NIDDM445	49	Female	Obese	90	111	203	35	167	79	Hypertension Stage 2 (Systolic >140 Diastolic >90)
NIDDM446	72	Male	Normal	60	218	173	29	138	41	Hypertension Stage 2 (Systolic >140 Diastolic >90)
NIDDM447	40	Male	Normal	100	216	188	312	92	51	Hypertension Stage 2 (Systolic >140 Diastolic >90)
NIDDM448	43	Female	Normal	70	210	189	460	136	65	Hypertension Stage 2 (Systolic >140 Diastolic >90)
NIDDM449	58	Female	Normal	150	213	183	88	80	58	Hypertension Stage 2 (Systolic >140 Diastolic >90)
NIDDM450	47	Male	Obese	140	225	205	360	190	60	Hypertension Stage 2 (Systolic >140 Diastolic >90)
NIDDM451	57	Female	Obese	80	220	187	88	178	50	Elevated (Systolic 120-129 Diastolic <80)
NIDDM452	58	Male	Normal	120	224	192	481	135	35	Elevated (Systolic 120-129 Diastolic <80)
NIDDM453	45	Male	Obese	88	214	190	43	198	63	Hypertension Stage 2 (Systolic >140 Diastolic >90)
NIDDM454	57	Female	Normal	215	217	172	81	99	39	Hypertension Stage 2 (Systolic >140 Diastolic >90)
NIDDM455	67	Male	Normal	76	210	182	388	67	51	Hypertension Stage 2 (Systolic >140 Diastolic >90)
NIDDM456	68	Female	Normal	215	218	197	401	94	55	Hypertension Stage 2 (Systolic >140 Diastolic >90)
NIDDM457	46	Female	Obese	214	216	200	103	135	15	Hypertension Stage 2 (Systolic >140 Diastolic >90)
NIDDM458	70	Male	Normal	146	219	187	392	192	47	Hypertension Stage 2 (Systolic >140 Diastolic >90)
NIDDM459	42	Female	Normal	153	210	189	290	220	38	Hypertension Stage 2 (Systolic >140 Diastolic >90)
NIDDM460	40	Female	Obese	300	157	184	380	72	44	Elevated (Systolic 120-129 Diastolic <80)
NIDDM461	66	Female	Normal	310	215	194	310	180	42	Hypertension Stage 2 (Systolic >140 Diastolic >90)
NIDDM462	61	Female	Normal	80	216	175	280	190	60	Hypertension Stage 2 (Systolic >140 Diastolic >90)
NIDDM463	42	Male	Normal	110	118	183	289	70	46	Hypertension Stage 2 (Systolic >140 Diastolic >90)
NIDDM464	39	Male	Normal	410	216	179	378	78	27	Hypertension Stage 2 (Systolic >140 Diastolic >90)
NIDDM465	62	Female	Normal	420	210	194	387	73	36	Hypertension Stage 2 (Systolic >140 Diastolic >90)
NIDDM466	45	Male	Normal	110	216	198	298	185	66	Hypertension Stage 2 (Systolic >140 Diastolic >90)
NIDDM467	41	Male	Normal	231	213	179	345	79	52	Hypertension Stage 2 (Systolic >140 Diastolic >90)
NIDDM468	46	Female	Obese	164	220	189	302	89	34	Hypertension Stage 2 (Systolic >140 Diastolic >90)
NIDDM469	41	Female	Obese	150	219	196	90	77	42	Elevated (Systolic 120-129 Diastolic <80)
NIDDM470	22	Female	Obese	200	218	204	78	99	9	Hypertension Stage 2 (Systolic >140 Diastolic >90)
NIDDM471	72	Female	Normal	215	222	104	21	96	64	Hypertension Stage 2 (Systolic >140 Diastolic >90)
NIDDM472	56	Female	Normal	146	220	140	298	196	59	Hypertension Stage 2 (Systolic >140 Diastolic >90)
NIDDM473	44	Female	Normal	76	219	207	398	123	60	Hypertension Stage 2 (Systolic >140 Diastolic >90)
NIDDM474	41	Female	Normal	89	215	117	376	76	43	Hypertension Stage 2 (Systolic >140 Diastolic >90)
NIDDM475	46	Female	Normal	213	218	187	387	195	8	Hypertension Stage 2 (Systolic >140 Diastolic >90)
NIDDM476	68	Female	Normal	143	215	143	401	76	55	Hypertension Stage 2 (Systolic >140 Diastolic >90)
NIDDM477	42	Female	Normal	98	213	139	392	124	65	Elevated (Systolic 120-129 Diastolic <80)
NIDDM478	40	Male	Normal	164	225	160	393	93	60	Hypertension Stage 2 (Systolic >140 Diastolic >90)
NIDDM479	46	Male	Normal	211	110	138	401	71	62	Hypertension Stage 2 (Systolic >140 Diastolic >90)
NIDDM480	62	Male	Normal	322	216	115	290	133	29	Hypertension Stage 2 (Systolic >140 Diastolic >90)
NIDDM481	50	Female	Normal	94	213	130	278	119	67	Hypertension Stage 2 (Systolic >140 Diastolic >90)
NIDDM482	47	Male	Normal	49	210	123	310	135	56	Hypertension Stage 2 (Systolic >140 Diastolic >90)
NIDDM483	41	Female	Normal	86	218	155	377	130	16	Hypertension Stage 2 (Systolic >140 Diastolic >90)
NIDDM484	49	Female	Normal	65	224	202	83	90	61	Hypertension Stage 2 (Systolic >140 Diastolic >90)
NIDDM485	52	Female	Normal	156	220	120	382	91	26	Hypertension Stage 2 (Systolic >140 Diastolic >90)
NIDDM486	52	Female	Normal	146	215	185	289	88	42	Elevated (Systolic 120-129 Diastolic <80)
NIDDM487	27	Female	Normal	348	210	178	290	113	44	Hypertension Stage 2 (Systolic >140 Diastolic >90)
NIDDM488	45	Female	Normal	176	216	201	70	133	9	Hypertension Stage 2 (Systolic >140 Diastolic >90)
NIDDM489	47	Male	Normal	86	210	176	350	77	41	Hypertension Stage 2 (Systolic >140 Diastolic >90)
NIDDM490	41	Female	Normal	99	217	176	250	129	31	Hypertension Stage 2 (Systolic >140 Diastolic >90)
NIDDM491	56	Female	Normal	86	216	240	290	55	24	Hypertension Stage 2 (Systolic >140 Diastolic >90)
NIDDM492	61	Female	Normal	141	210	235	310	86	71	Hypertension Stage 2 (Systolic >140 Diastolic >90)
NIDDM493	43	Male	Normal	141	216	200	292	99	51	Hypertension Stage 2 (Systolic >140 Diastolic >90)
NIDDM494	55	Female	Normal	300	213	199	367	118	50	Hypertension Stage 2 (Systolic >140 Diastolic >90)
NIDDM495	26	Female	Normal	335	218	206	318	107	43	Hypertension Stage 2 (Systolic >140 Diastolic >90)
NIDDM496	40	Male	Normal	146	210	110	364	65	64	Hypertension Stage 2 (Systolic >140 Diastolic >90)
NIDDM497	25	Male	Normal	125	256	280	370	60	46	Hypertension Stage 2 (Systolic >140 Diastolic >90)
NIDDM498	53	Female	Normal	86	218	234	80	78	36	Hypertension Stage 2 (Systolic >140 Diastolic >90)
NIDDM499	41	Female	Normal	94	254	120	64	70	44	Hypertension Stage 2 (Systolic >140 Diastolic >90)
NIDDM500	46	Male	Normal	75	265	230	114	75	36	Hypertension Stage 2 (Systolic >140 Diastolic >90)
NIDDM501	64	Male	Normal	246	398	280	96	190	52	Hypertension Stage 2 (Systolic >140 Diastolic >90)

NIDDM502	29	Female	Normal	146	218	230	120	150	56	Hypertension Stage 2 (Systolic >140 Diastolic >90)
NIDDM503	44	Male	Normal	91	235	223	78	82	73	Hypertension Stage 2 (Systolic >140 Diastolic >90)
NIDDM504	55	Male	Normal	175	237	120	322	82	36	Hypertension Stage 2 (Systolic >140 Diastolic >90)
NIDDM505	41	Female	Normal	165	256	230	315	178	51	Hypertension Stage 2 (Systolic >140 Diastolic >90)
NIDDM506	54	Female	Normal	153	260	240	190	121	43	Hypertension Stage 2 (Systolic >140 Diastolic >90)
NIDDM507	47	Male	Normal	213	496	102	56	201	11	Hypertension Stage 2 (Systolic >140 Diastolic >90)
NIDDM508	61	Female	Normal	258	252	202	67	92	13	Hypertension Stage 2 (Systolic >140 Diastolic >90)
NIDDM509	61	Female	Normal	246	255	195	93	87	8	Hypertension Stage 2 (Systolic >140 Diastolic >90)
NIDDM510	43	Male	Normal	88	395	179	81	68	27	Hypertension Stage 2 (Systolic >140 Diastolic >90)
NIDDM511	60	Female	Normal	89	235	310	120	170	33	Hypertension Stage 2 (Systolic >140 Diastolic >90)
NIDDM512	42	Female	Normal	153	218	196	108	86	47	Hypertension Stage 2 (Systolic >140 Diastolic >90)
NIDDM513	62	Female	Normal	64	210	201	74	127	19	Hypertension Stage 2 (Systolic >140 Diastolic >90)
NIDDM514	53	Female	Normal	358	216	202	93	156	31	Hypertension Stage 2 (Systolic >140 Diastolic >90)
NIDDM515	58	Female	Normal	86	256	160	120	136	44	Hypertension Stage 2 (Systolic >140 Diastolic >90)
NIDDM516	48	Male	Normal	215	287	162	134	193	19	Hypertension Stage 2 (Systolic >140 Diastolic >90)
NIDDM517	59	Female	Normal	145	256	140	387	132	53	Hypertension Stage 2 (Systolic >140 Diastolic >90)
NIDDM518	53	Female	Normal	89	257	204	524	230	32	Hypertension Stage 2 (Systolic >140 Diastolic >90)
NIDDM519	49	Female	Normal	253	374	132	319	122	49	Hypertension Stage 2 (Systolic >140 Diastolic >90)
NIDDM520	46	Female	Normal	164	246	150	401	213	55	Hypertension Stage 2 (Systolic >140 Diastolic >90)
NIDDM521	60	Female	Normal	145	268	115	77	128	56	Hypertension Stage 2 (Systolic >140 Diastolic >90)
NIDDM522	40	Male	Normal	76	258	123	381	92	40	Hypertension Stage 2 (Systolic >140 Diastolic >90)
NIDDM523	32	Male	Normal	214	136	278	72	74	35	Hypertension Stage 2 (Systolic >140 Diastolic >90)
NIDDM524	29	Male	Normal	128	410	112	401	96	56	Hypertension Stage 2 (Systolic >140 Diastolic >90)
NIDDM525	28	Female	Normal	145	121	230	310	68	43	Hypertension Stage 2 (Systolic >140 Diastolic >90)
NIDDM526	67	Female	Normal	160	130	240	390	79	37	Hypertension Stage 2 (Systolic >140 Diastolic >90)
NIDDM527	60	Female	Normal	406	123	278	74	68	16	Hypertension Stage 2 (Systolic >140 Diastolic >90)
NIDDM528	56	Female	Normal	249	140	118	423	81	47	Hypertension Stage 2 (Systolic >140 Diastolic >90)
NIDDM529	42	Female	Normal	100	224	192	336	94	20	Hypertension Stage 2 (Systolic >140 Diastolic >90)
NIDDM530	39	Female	Normal	176	257	130	340	90	48	Hypertension Stage 2 (Systolic >140 Diastolic >90)
NIDDM531	33	Female	Normal	164	253	145	100	238	62	Hypertension Stage 2 (Systolic >140 Diastolic >90)
NIDDM532	46	Female	Normal	91	265	150	56	92	48	Hypertension Stage 2 (Systolic >140 Diastolic >90)
NIDDM533	60	Female	Normal	87	298	203	447	95	19	Hypertension Stage 2 (Systolic >140 Diastolic >90)
NIDDM534	57	Female	Normal	115	230	108	99	77	64	Hypertension Stage 2 (Systolic >140 Diastolic >90)
NIDDM535	56	Female	Normal	276	279	202	69	76	39	Hypertension Stage 2 (Systolic >140 Diastolic >90)
NIDDM536	56	Female	Normal	173	310	265	95	70	42	Hypertension Stage 2 (Systolic >140 Diastolic >90)
NIDDM537	42	Female	Normal	94	299	250	384	75	17	Hypertension Stage 2 (Systolic >140 Diastolic >90)
NIDDM538	39	Female	Normal	146	265	245	374	80	53	Hypertension Stage 2 (Systolic >140 Diastolic >90)
NIDDM539	42	Female	Normal	99	248	123	390	189	36	Hypertension Stage 2 (Systolic >140 Diastolic >90)
NIDDM540	32	Female	Normal	217	621	112	356	99	49	Hypertension Stage 2 (Systolic >140 Diastolic >90)
NIDDM541	61	Male	Normal	86	277	103	371	68	41	Hypertension Stage 2 (Systolic >140 Diastolic >90)
NIDDM542	26	Male	Normal	143	265	132	90	189	48	Hypertension Stage 2 (Systolic >140 Diastolic >90)
NIDDM543	41	Female	Normal	218	269	235	396	150	56	Hypertension Stage 2 (Systolic >140 Diastolic >90)
NIDDM544	54	Male	Normal	156	235	230	440	170	50	Hypertension Stage 2 (Systolic >140 Diastolic >90)
NIDDM545	45	Female	Normal	86	284	119	67	85	49	Hypertension Stage 2 (Systolic >140 Diastolic >90)
NIDDM546	58	Female	Normal	91	248	256	377	178	24	Hypertension Stage 2 (Systolic >140 Diastolic >90)
NIDDM547	46	Female	Normal	216	146	134	371	178	42	Hypertension Stage 2 (Systolic >140 Diastolic >90)
NIDDM548	54	Female	Normal	91	185	123	56	80	32	Hypertension Stage 2 (Systolic >140 Diastolic >90)
NIDDM549	43	Female	Normal	156	254	140	59	91	30	Hypertension Stage 2 (Systolic >140 Diastolic >90)
NIDDM550	51	Male	Normal	76	215	280	399	92	25	Hypertension Stage 2 (Systolic >140 Diastolic >90)
NIDDM551	61	Male	Normal	59	260	204	321	156	9	Hypertension Stage 2 (Systolic >140 Diastolic >90)
NIDDM552	55	Male	Normal	86	277	210	67	167	50	Hypertension Stage 2 (Systolic >140 Diastolic >90)
NIDDM553	47	Female	Normal	341	235	123	81	88	36	Hypertension Stage 2 (Systolic >140 Diastolic >90)
NIDDM554	39	Female	Normal	156	224	240	397	93	29	Hypertension Stage 2 (Systolic >140 Diastolic >90)
NIDDM555	43	Male	Normal	221	157	278	390	99	42	Hypertension Stage 2 (Systolic >140 Diastolic >90)
NIDDM556	40	Female	Normal	186	256	142	92	170	55	Hypertension Stage 2 (Systolic >140 Diastolic >90)
NIDDM557	43	Male	Normal	386	287	123	381	180	49	Hypertension Stage 2 (Systolic >140 Diastolic >90)
NIDDM558	40	Female	Normal	297	247	204	358	77	72	Hypertension Stage 2 (Systolic >140 Diastolic >90)
NIDDM559	58	Female	Normal	343	215	108	378	67	67	Hypertension Stage 2 (Systolic >140 Diastolic >90)
NIDDM560	60	Male	Normal	256	265	113	90	93	11	Hypertension Stage 2 (Systolic >140 Diastolic >90)
NIDDM561	43	Male	Normal	82	234	140	90	99	60	Hypertension Stage 2 (Systolic >140 Diastolic >90)
NIDDM562	49	Female	Normal	98	278	122	89	72	36	Hypertension Stage 2 (Systolic >140 Diastolic >90)
NIDDM563	47	Male	Normal	391	288	119	450	178	37	Hypertension Stage 2 (Systolic >140 Diastolic >90)
NIDDM564	60	Female	Normal	343	265	234	336	160	45	Hypertension Stage 2 (Systolic >140 Diastolic >90)
NIDDM565	47	Female	Normal	283	287	111	80	150	27	Hypertension Stage 2 (Systolic >140 Diastolic >90)
NIDDM566	59	Male	Normal	243	236	134	378	132	22	Hypertension Stage 2 (Systolic >140 Diastolic >90)
NIDDM567	53	Female	Normal	183	254	204	370	92	11	Hypertension Stage 2 (Systolic >140 Diastolic >90)
NIDDM568	62	Male	Normal	146	162	155	91	78	61	Hypertension Stage 2 (Systolic >140 Diastolic >90)
NIDDM569	62	Female	Normal	301	285	140	370	96	32	Hypertension Stage 2 (Systolic >140 Diastolic >90)
NIDDM570	41	Female	Normal	73	268	132	99	130	48	Hypertension Stage 2 (Systolic >140 Diastolic >90)
NIDDM571	39	Female	Normal	99	249	153	32	186	29	Hypertension Stage 2 (Systolic >140 Diastolic >90)
NIDDM572	60	Female	Normal	108	247	207	67	138	38	Hypertension Stage 2 (Systolic >140 Diastolic >90)
NIDDM573	53	Male	Normal	134	246	205	67	88	76	Hypertension Stage 2 (Systolic >140 Diastolic >90)
NIDDM574	46	Female	Normal	146	248	201	56	73	43	Hypertension Stage 2 (Systolic >140 Diastolic >90)
NIDDM575	56	Male	Normal	286	255	253	70	87	10	Hypertension Stage 2 (Systolic >140 Diastolic >90)
NIDDM576	40	Female	Normal	145	222	129	407	85	37	Hypertension Stage 2 (Systolic >140 Diastolic >90)
NIDDM577	50	Female	Normal	308	244	164	330	173	72	Hypertension Stage 2 (Systolic >140 Diastolic >90)
NIDDM578	51	Female	Normal	175	248	121	412	165	38	Hypertension Stage 2 (Systolic >140 Diastolic >90)
NIDDM579	55	Female	Normal	274	265	122	334	89	29	Hypertension Stage 2 (Systolic >140 Diastolic >90)
NIDDM580	46	Male	Normal	247	247	123	75	187	30	Hypertension Stage 2 (Systolic >140 Diastolic >90)
NIDDM581	48	Female	Normal	123	247	212	395	99	35	Hypertension Stage 2 (Systolic >140 Diastolic >90)
NIDDM582	48	Female	Normal	77	248	208	356	126	18	Hypertension Stage 2 (Systolic >140 Diastolic >90)
NIDDM583	44	Female	Normal	198	265	130	350	122	45	Hypertension Stage 2 (Systolic >140 Diastolic >90)
NIDDM584	58	Female	Normal	146	231	135	67	82	60	Hypertension Stage 2 (Systolic >140 Diastolic >90)
NIDDM585	44	Female	Normal	88	243	140	344	135	46	Hypertension Stage 2 (Systolic >140 Diastolic >90)

NIDDM586	44	Female	Normal	119	268	135	388	76	36	Hypertension Stage 2 (Systolic >140 Diastolic >90)
NIDDM587	51	Male	Normal	400	299	200	86	190	39	Hypertension Stage 2 (Systolic >140 Diastolic >90)
NIDDM588	42	Female	Normal	280	274	245	345	68	33	Hypertension Stage 2 (Systolic >140 Diastolic >90)
NIDDM589	42	Female	Normal	130	163	202	387	180	50	Hypertension Stage 2 (Systolic >140 Diastolic >90)
NIDDM590	49	Female	Normal	230	275	125	98	82	7	Hypertension Stage 2 (Systolic >140 Diastolic >90)
NIDDM591	51	Male	Normal	300	251	132	390	81	58	Hypertension Stage 2 (Systolic >140 Diastolic >90)
NIDDM592	54	Female	Normal	100	249	128	70	83	35	Hypertension Stage 2 (Systolic >140 Diastolic >90)
NIDDM593	62	Female	Normal	260	265	210	96	70	27	Hypertension Stage 2 (Systolic >140 Diastolic >90)
NIDDM594	51	Female	Normal	420	254	200	341	132	55	Hypertension Stage 2 (Systolic >140 Diastolic >90)
NIDDM595	60	Male	Normal	88	361	167	345	97	51	Hypertension Stage 2 (Systolic >140 Diastolic >90)
NIDDM596	52	Female	Normal	76	222	185	325	102	24	Hypertension Stage 2 (Systolic >140 Diastolic >90)
NIDDM597	44	Male	Normal	84	288	203	390	127	34	Hypertension Stage 2 (Systolic >140 Diastolic >90)
NIDDM598	49	Female	Normal	94	235	199	81	112	32	Hypertension Stage 2 (Systolic >140 Diastolic >90)
NIDDM599	57	Female	Normal	80	231	234	354	126	52	Hypertension Stage 2 (Systolic >140 Diastolic >90)
NIDDM600	49	Female	Normal	75	297	135	78	95	38	Hypertension Stage 2 (Systolic >140 Diastolic >90)
NIDDM601	58	Female	Normal	115	300	196	366	94	58	Hypertension Stage 2 (Systolic >140 Diastolic >90)
NIDDM602	41	Female	Normal	249	265	190	319	118	21	Hypertension Stage 1 (Systolic 130-139 Diastolic 80-89)
NIDDM603	61	Female	Normal	75	213	170	375	137	8	Hypertension Stage 1 (Systolic 130-139 Diastolic 80-89)
NIDDM604	53	Female	Normal	70	298	202	85	116	40	Hypertension Stage 1 (Systolic 130-139 Diastolic 80-89)
NIDDM605	56	Female	Normal	450	245	174	336	77	25	Hypertension Stage 1 (Systolic 130-139 Diastolic 80-89)
NIDDM606	57	Male	Normal	100	264	165	90	99	43	Hypertension Stage 1 (Systolic 130-139 Diastolic 80-89)
NIDDM607	59	Female	Normal	76	239	198	313	130	33	Hypertension Stage 1 (Systolic 130-139 Diastolic 80-89)
NIDDM608	59	Female	Normal	218	274	197	90	109	14	Hypertension Stage 2 (Systolic >140 Diastolic >90)
NIDDM609	53	Male	Normal	94	173	178	331	86	15	Hypertension Stage 1 (Systolic 130-139 Diastolic 80-89)
NIDDM610	46	Male	Normal	76	520	200	321	112	35	Hypertension Stage 1 (Systolic 130-139 Diastolic 80-89)
NIDDM611	41	Female	Normal	90	265	177	59	79	54	Hypertension Stage 1 (Systolic 130-139 Diastolic 80-89)
NIDDM612	62	Male	Normal	99	235	160	90	70	60	Hypertension Stage 2 (Systolic >140 Diastolic >90)
NIDDM613	52	Female	Normal	418	117	192	82	107	15	Hypertension Stage 1 (Systolic 130-139 Diastolic 80-89)
NIDDM614	57	Male	Normal	261	264	201	300	139	17	Hypertension Stage 1 (Systolic 130-139 Diastolic 80-89)
NIDDM615	62	Male	Normal	275	298	202	74	73	18	Hypertension Stage 1 (Systolic 130-139 Diastolic 80-89)
NIDDM616	61	Female	Normal	100	237	189	67	77	22	Hypertension Stage 1 (Systolic 130-139 Diastolic 80-89)
NIDDM617	57	Female	Normal	76	216	187	312	132	57	Hypertension Stage 1 (Systolic 130-139 Diastolic 80-89)
NIDDM618	55	Male	Normal	258	245	186	97	121	57	Hypertension Stage 1 (Systolic 130-139 Diastolic 80-89)
NIDDM619	40	Female	Normal	112	357	190	81	104	42	Hypertension Stage 1 (Systolic 130-139 Diastolic 80-89)
NIDDM620	55	Female	Normal	253	278	178	362	106	32	Hypertension Stage 1 (Systolic 130-139 Diastolic 80-89)
NIDDM621	57	Male	Normal	86	265	200	421	118	58	Hypertension Stage 1 (Systolic 130-139 Diastolic 80-89)
NIDDM622	54	Male	Normal	349	243	207	378	131	24	Hypertension Stage 1 (Systolic 130-139 Diastolic 80-89)
NIDDM623	48	Male	Normal	144	244	174	59	139	26	Hypertension Stage 1 (Systolic 130-139 Diastolic 80-89)
NIDDM624	62	Female	Normal	79	241	177	371	101	47	Hypertension Stage 1 (Systolic 130-139 Diastolic 80-89)
NIDDM625	55	Male	Normal	314	235	109	46	109	58	Hypertension Stage 1 (Systolic 130-139 Diastolic 80-89)
NIDDM626	55	Male	Normal	112	566	182	88	79	13	Hypertension Stage 1 (Systolic 130-139 Diastolic 80-89)
NIDDM627	51	Female	Normal	100	248	201	362	86	48	Hypertension Stage 1 (Systolic 130-139 Diastolic 80-89)
NIDDM628	45	Female	Normal	285	391	173	81	104	49	Hypertension Stage 2 (Systolic >140 Diastolic >90)
NIDDM629	50	Male	Normal	246	294	113	390	112	31	Hypertension Stage 1 (Systolic 130-139 Diastolic 80-89)
NIDDM630	57	Male	Normal	115	251	207	365	108	74	Hypertension Stage 1 (Systolic 130-139 Diastolic 80-89)
NIDDM631	51	Male	Normal	115	132	198	350	112	50	Hypertension Stage 2 (Systolic >140 Diastolic >90)
NIDDM632	41	Female	Normal	105	247	181	390	78	65	Hypertension Stage 1 (Systolic 130-139 Diastolic 80-89)
NIDDM633	45	Male	Normal	120	264	195	308	85	42	Hypertension Stage 1 (Systolic 130-139 Diastolic 80-89)
NIDDM634	50	Female	Normal	333	248	130	89	127	42	Hypertension Stage 1 (Systolic 130-139 Diastolic 80-89)
NIDDM635	42	Male	Normal	145	249	142	378	86	51	Hypertension Stage 2 (Systolic >140 Diastolic >90)
NIDDM636	54	Male	Normal	153	98	256	301	101	11	Hypertension Stage 1 (Systolic 130-139 Diastolic 80-89)
NIDDM637	59	Female	Normal	115	267	137	67	129	62	Hypertension Stage 1 (Systolic 130-139 Diastolic 80-89)
NIDDM638	45	Female	Normal	77	271	188	63	118	43	Hypertension Stage 1 (Systolic 130-139 Diastolic 80-89)
NIDDM639	49	Female	Normal	258	249	193	397	72	34	Hypertension Stage 2 (Systolic >140 Diastolic >90)
NIDDM640	43	Male	Normal	301	562	206	45	116	38	Hypertension Stage 1 (Systolic 130-139 Diastolic 80-89)
NIDDM641	62	Male	Normal	231	98	131	389	135	46	Hypertension Stage 1 (Systolic 130-139 Diastolic 80-89)
NIDDM642	59	Female	Normal	96	237	190	67	98	34	Hypertension Stage 1 (Systolic 130-139 Diastolic 80-89)
NIDDM643	58	Female	Normal	113	248	132	375	128	41	Hypertension Stage 1 (Systolic 130-139 Diastolic 80-89)
NIDDM644	58	Female	Normal	110	246	206	370	86	55	Hypertension Stage 1 (Systolic 130-139 Diastolic 80-89)
NIDDM645	23	Female	Normal	258	138	128	325	82	47	Hypertension Stage 1 (Systolic 130-139 Diastolic 80-89)
NIDDM646	40	Male	Normal	446	89	206	56	82	16	Hypertension Stage 2 (Systolic >140 Diastolic >90)
NIDDM647	62	Male	Normal	81	151	195	358	84	36	Hypertension Stage 1 (Systolic 130-139 Diastolic 80-89)
NIDDM648	40	Female	Normal	243	356	177	337	88	45	Hypertension Stage 1 (Systolic 130-139 Diastolic 80-89)
NIDDM649	41	Female	Normal	156	278	191	89	129	37	Hypertension Stage 1 (Systolic 130-139 Diastolic 80-89)
NIDDM650	48	Female	Normal	84	268	234	360	79	56	Hypertension Stage 1 (Systolic 130-139 Diastolic 80-89)
NIDDM651	40	Female	Normal	231	152	222	390	69	33	Hypertension Stage 1 (Systolic 130-139 Diastolic 80-89)
NIDDM652	45	Female	Normal	216	246	249	38	82	43	Hypertension Stage 1 (Systolic 130-139 Diastolic 80-89)
NIDDM653	70	Female	Normal	73	149	177	94	80	12	Hypertension Stage 1 (Systolic 130-139 Diastolic 80-89)
NIDDM654	45	Female	Normal	163	231	202	392	107	32	Hypertension Stage 1 (Systolic 130-139 Diastolic 80-89)
NIDDM655	58	Female	Normal	91	265	202	404	130	45	Hypertension Stage 1 (Systolic 130-139 Diastolic 80-89)
NIDDM656	67	Female	Normal	102	237	120	94	102	37	Hypertension Stage 1 (Systolic 130-139 Diastolic 80-89)
NIDDM657	48	Male	Normal	79	360	123	341	94	46	Hypertension Stage 1 (Systolic 130-139 Diastolic 80-89)
NIDDM658	40	Female	Normal	96	297	134	67	110	35	Hypertension Stage 2 (Systolic >140 Diastolic >90)
NIDDM659	49	Female	Normal	100	246	235	376	74	37	Hypertension Stage 1 (Systolic 130-139 Diastolic 80-89)
NIDDM660	25	Female	Normal	219	248	260	32	113	31	Hypertension Stage 1 (Systolic 130-139 Diastolic 80-89)
NIDDM661	57	Female	Normal	97	243	267	82	109	65	Hypertension Stage 1 (Systolic 130-139 Diastolic 80-89)
NIDDM662	23	Male	Normal	213	267	280	59	101	57	Hypertension Stage 1 (Systolic 130-139 Diastolic 80-89)
NIDDM663	40	Female	Normal	253	156	235	348	116	37	Hypertension Stage 1 (Systolic 130-139 Diastolic 80-89)
NIDDM664	59	Male	Normal	246	237	280	380	104	68	Hypertension Stage 1 (Systolic 130-139 Diastolic 80-89)
NIDDM665	66	Male	Normal	102	287	267	86	111	7	Hypertension Stage 1 (Systolic 130-139 Diastolic 80-89)
NIDDM666	24	Female	Normal	192	300	310	67	128	20	Hypertension Stage 1 (Systolic 130-139 Diastolic 80-89)
NIDDM667	65	Female	Normal	246	284	123	90	110	53	Hypertension Stage 1 (Systolic 130-139 Diastolic 80-89)
NIDDM668	68	Male	Normal	98	379	196	317	109	12	Hypertension Stage 2 (Systolic >140 Diastolic >90)
NIDDM669	66	Female	Normal	186	248	237	311	123	56	Hypertension Stage 1 (Systolic 130-139 Diastolic 80-89)

NIDDM670	43	Female	Normal	79	264	249	400	96	17	Hypertension Stage 1 (Systolic 130-139 Diastolic 80-89)
NIDDM671	36	Male	Normal	252	156	195	90	118	48	Hypertension Stage 1 (Systolic 130-139 Diastolic 80-89)
NIDDM672	24	Female	Normal	77	569	214	390	73	50	Hypertension Stage 1 (Systolic 130-139 Diastolic 80-89)
NIDDM673	26	Female	Normal	350	118	116	340	97	46	Hypertension Stage 1 (Systolic 130-139 Diastolic 80-89)
NIDDM674	62	Male	Normal	218	246	223	91	126	8	Hypertension Stage 1 (Systolic 130-139 Diastolic 80-89)
NIDDM675	70	Female	Obese	219	235	193	45	104	38	Hypertension Stage 1 (Systolic 130-139 Diastolic 80-89)
NIDDM676	61	Female	Obese	198	245	234	335	116	9	Hypertension Stage 2 (Systolic >140 Diastolic >90)
NIDDM677	39	Male	Obese	186	485	194	311	93	42	Hypertension Stage 1 (Systolic 130-139 Diastolic 80-89)
NIDDM678	59	Female	Obese	198	156	162	434	136	23	Hypertension Stage 1 (Systolic 130-139 Diastolic 80-89)
NIDDM679	33	Male	Obese	249	298	123	320	120	22	Hypertension Stage 1 (Systolic 130-139 Diastolic 80-89)
NIDDM680	66	Female	Obese	245	297	196	96	101	34	Hypertension Stage 1 (Systolic 130-139 Diastolic 80-89)
NIDDM681	24	Male	Normal	87	264	276	67	134	35	Hypertension Stage 1 (Systolic 130-139 Diastolic 80-89)
NIDDM682	67	Female	Obese	89	232	140	326	135	11	Hypertension Stage 1 (Systolic 130-139 Diastolic 80-89)
NIDDM683	43	Female	Obese	87	246	240	332	104	50	Hypertension Stage 1 (Systolic 130-139 Diastolic 80-89)
NIDDM684	25	Female	Obese	352	163	247	329	100	44	Hypertension Stage 2 (Systolic >140 Diastolic >90)
NIDDM685	60	Female	Obese	269	256	120	44	91	60	Hypertension Stage 1 (Systolic 130-139 Diastolic 80-89)
NIDDM686	39	Female	Obese	299	142	234	81	107	49	Hypertension Stage 1 (Systolic 130-139 Diastolic 80-89)
NIDDM687	25	Female	Over Weight	281	267	150	67	115	14	Hypertension Stage 1 (Systolic 130-139 Diastolic 80-89)
NIDDM688	28	Male	Normal	88	168	234	322	71	36	Hypertension Stage 1 (Systolic 130-139 Diastolic 80-89)
NIDDM689	27	Female	Over Weight	145	297	267	89	88	14	Hypertension Stage 1 (Systolic 130-139 Diastolic 80-89)
NIDDM690	22	Female	Over Weight	90	248	123	325	135	54	Hypertension Stage 1 (Systolic 130-139 Diastolic 80-89)
NIDDM691	71	Female	Over Weight	246	266	240	324	113	7	Hypertension Stage 1 (Systolic 130-139 Diastolic 80-89)
NIDDM692	26	Female	Over Weight	256	102	102	67	117	37	Hypertension Stage 1 (Systolic 130-139 Diastolic 80-89)
NIDDM693	42	Female	Normal	246	258	123	34	109	32	Hypertension Stage 1 (Systolic 130-139 Diastolic 80-89)
NIDDM694	59	Male	Over Weight	194	374	267	58	107	43	Hypertension Stage 1 (Systolic 130-139 Diastolic 80-89)
NIDDM695	39	Male	Over Weight	110	249	120	366	77	51	Hypertension Stage 1 (Systolic 130-139 Diastolic 80-89)
NIDDM696	59	Female	Over Weight	102	96	128	321	74	21	Hypertension Stage 1 (Systolic 130-139 Diastolic 80-89)
NIDDM697	54	Female	Normal	98	264	130	381	70	25	Hypertension Stage 1 (Systolic 130-139 Diastolic 80-89)
NIDDM698	59	Female	Over Weight	246	297	141	99	97	20	Hypertension Stage 1 (Systolic 130-139 Diastolic 80-89)
NIDDM699	54	Female	Over Weight	268	264	280	334	132	61	Hypertension Stage 2 (Systolic >140 Diastolic >90)
NIDDM700	58	Female	Over Weight	186	112	112	61	84	73	Hypertension Stage 1 (Systolic 130-139 Diastolic 80-89)
NIDDM701	54	Female	Over Weight	112	279	265	333	115	40	Hypertension Stage 1 (Systolic 130-139 Diastolic 80-89)
NIDDM702	40	Male	Over Weight	267	248	126	317	88	59	Hypertension Stage 1 (Systolic 130-139 Diastolic 80-89)
NIDDM703	41	Female	Over Weight	176	115	134	390	134	42	Hypertension Stage 1 (Systolic 130-139 Diastolic 80-89)
NIDDM704	50	Female	Normal	100	298	237	321	126	36	Hypertension Stage 1 (Systolic 130-139 Diastolic 80-89)
NIDDM705	45	Male	Normal	99	243	129	89	124	58	Hypertension Stage 1 (Systolic 130-139 Diastolic 80-89)
NIDDM706	55	Female	Normal	153	249	284	82	123	35	Hypertension Stage 1 (Systolic 130-139 Diastolic 80-89)
NIDDM707	68	Male	Normal	216	218	123	54	109	40	Hypertension Stage 1 (Systolic 130-139 Diastolic 80-89)
NIDDM708	61	Female	Normal	102	456	230	334	82	63	Hypertension Stage 1 (Systolic 130-139 Diastolic 80-89)
NIDDM709	42	Female	Normal	152	267	112	75	138	29	Hypertension Stage 1 (Systolic 130-139 Diastolic 80-89)
NIDDM710	57	Male	Normal	194	234	125	79	75	50	Hypertension Stage 1 (Systolic 130-139 Diastolic 80-89)
NIDDM711	48	Female	Normal	77	229	295	393	109	64	Hypertension Stage 1 (Systolic 130-139 Diastolic 80-89)
NIDDM712	46	Female	Normal	218	230	120	345	114	9	Hypertension Stage 1 (Systolic 130-139 Diastolic 80-89)
NIDDM713	50	Male	Normal	81	260	132	311	113	42	Hypertension Stage 1 (Systolic 130-139 Diastolic 80-89)
NIDDM714	46	Female	Over Weight	96	248	202	373	114	44	Hypertension Stage 2 (Systolic >140 Diastolic >90)
NIDDM715	53	Male	Over Weight	94	480	111	74	114	65	Hypertension Stage 1 (Systolic 130-139 Diastolic 80-89)
NIDDM716	44	Male	Normal	191	388	276	85	99	55	Hypertension Stage 1 (Systolic 130-139 Diastolic 80-89)
NIDDM717	45	Male	Over Weight	245	247	260	308	105	51	Hypertension Stage 1 (Systolic 130-139 Diastolic 80-89)
NIDDM718	50	Female	Over Weight	100	241	123	361	111	13	Hypertension Stage 1 (Systolic 130-139 Diastolic 80-89)
NIDDM719	53	Male	Obese	261	110	257	86	125	39	Hypertension Stage 1 (Systolic 130-139 Diastolic 80-89)
NIDDM720	53	Female	Obese	90	248	107	324	76	49	Hypertension Stage 1 (Systolic 130-139 Diastolic 80-89)
NIDDM721	58	Female	Obese	213	246	113	333	82	42	Hypertension Stage 1 (Systolic 130-139 Diastolic 80-89)
NIDDM722	58	Female	Obese	345	275	202	81	69	28	Hypertension Stage 1 (Systolic 130-139 Diastolic 80-89)
NIDDM723	72	Male	Obese	219	271	138	328	67	33	Hypertension Stage 1 (Systolic 130-139 Diastolic 80-89)
NIDDM724	56	Female	Obese	292	489	117	87	68	33	Hypertension Stage 1 (Systolic 130-139 Diastolic 80-89)
NIDDM725	56	Female	Obese	246	257	245	312	71	42	Hypertension Stage 1 (Systolic 130-139 Diastolic 80-89)
NIDDM726	59	Female	Obese	70	147	267	70	122	38	Hypertension Stage 1 (Systolic 130-139 Diastolic 80-89)
NIDDM727	60	Female	Obese	273	267	110	77	130	19	Hypertension Stage 1 (Systolic 130-139 Diastolic 80-89)
NIDDM728	56	Female	Obese	99	249	112	303	86	14	Hypertension Stage 1 (Systolic 130-139 Diastolic 80-89)
NIDDM729	55	Male	Obese	254	235	202	307	87	49	Hypertension Stage 1 (Systolic 130-139 Diastolic 80-89)
NIDDM730	70	Female	Obese	97	369	144	90	90	55	Hypertension Stage 1 (Systolic 130-139 Diastolic 80-89)
NIDDM731	62	Female	Obese	265	228	102	314	135	25	Hypertension Stage 1 (Systolic 130-139 Diastolic 80-89)
NIDDM732	59	Female	Obese	156	218	107	80	125	32	Hypertension Stage 1 (Systolic 130-139 Diastolic 80-89)
NIDDM733	57	Female	Normal	473	246	159	367	108	58	Hypertension Stage 1 (Systolic 130-139 Diastolic 80-89)
NIDDM734	39	Male	Obese	82	237	114	43	122	14	Hypertension Stage 1 (Systolic 130-139 Diastolic 80-89)
NIDDM735	40	Male	Obese	221	478	137	337	93	31	Hypertension Stage 2 (Systolic >140 Diastolic >90)
NIDDM736	49	Male	Obese	312	296	211	71	139	32	Hypertension Stage 1 (Systolic 130-139 Diastolic 80-89)
NIDDM737	59	Female	Obese	215	267	117	321	78	39	Hypertension Stage 1 (Systolic 130-139 Diastolic 80-89)
NIDDM738	57	Female	Obese	70	257	198	346	84	15	Hypertension Stage 1 (Systolic 130-139 Diastolic 80-89)
NIDDM739	48	Male	Obese	369	137	174	379	75	60	Hypertension Stage 2 (Systolic >140 Diastolic >90)
NIDDM740	50	Male	Obese	193	257	292	308	70	11	Hypertension Stage 2 (Systolic >140 Diastolic >90)
NIDDM741	53	Male	Obese	88	276	118	50	90	78	Hypertension Stage 1 (Systolic 130-139 Diastolic 80-89)
NIDDM742	47	Male	Obese	134	248	204	364	100	54	Hypertension Stage 1 (Systolic 130-139 Diastolic 80-89)
NIDDM743	52	Female	Obese	243	249	207	60	127	8	Hypertension Stage 1 (Systolic 130-139 Diastolic 80-89)
NIDDM744	44	Female	Obese	64	247	123	97	96	9	Hypertension Stage 2 (Systolic >140 Diastolic >90)
NIDDM745	60	Male	Normal	70	246	207	47	112	23	Hypertension Stage 2 (Systolic >140 Diastolic >90)
NIDDM746	62	Female	Obese	300	156	167	91	84	66	Hypertension Stage 1 (Systolic 130-139 Diastolic 80-89)
NIDDM747	46	Female	Obese	400	132	212	337	70	50	Hypertension Stage 1 (Systolic 130-139 Diastolic 80-89)
NIDDM748	46	Male	Obese	420	249	119	370	72	68	Hypertension Stage 1 (Systolic 130-139 Diastolic 80-89)
NIDDM749	46	Male	Over Weight	410	243	173	28	67	25	Hypertension Stage 1 (Systolic 130-139 Diastolic 80-89)
NIDDM750	54	Male	Over Weight	76	248	121	354	66	14	Hypertension Stage 1 (Systolic 130-139 Diastolic 80-89)
NIDDM751	46	Female	Obese	243	287	165	85	61	37	Hypertension Stage 2 (Systolic >140 Diastolic >90)
NIDDM752	61	Male	Over Weight	325	264	109	79	91	40	Hypertension Stage 1 (Systolic 130-139 Diastolic 80-89)
NIDDM753	52	Female	Obese	215	287	193	290	150	57	Hypertension Stage 1 (Systolic 130-139 Diastolic 80-89)

NIDDM754	48	Male	Over Weight	253	267	178	31	210	33	Hypertension Stage 1 (Systolic 130-139 Diastolic 80-89)
NIDDM755	53	Female	Obese	323	237	201	30	190	51	Hypertension Stage 1 (Systolic 130-139 Diastolic 80-89)
NIDDM756	60	Female	Obese	266	297	193	54	195	11	Hypertension Stage 1 (Systolic 130-139 Diastolic 80-89)
NIDDM757	39	Female	Obese	110	248	107	449	67	42	Hypertension Stage 2 (Systolic >140 Diastolic >90)
NIDDM758	56	Male	Normal	164	446	175	390	72	41	Hypertension Stage 2 (Systolic >140 Diastolic >90)
NIDDM759	53	Male	Over Weight	112	237	207	42	51	40	Hypertension Stage 1 (Systolic 130-139 Diastolic 80-89)
NIDDM760	41	Male	Over Weight	108	397	204	404	56	50	Hypertension Stage 1 (Systolic 130-139 Diastolic 80-89)
NIDDM761	60	Male	Over Weight	81	237	132	315	72	63	Hypertension Stage 1 (Systolic 130-139 Diastolic 80-89)
NIDDM762	42	Female	Obese	63	247	256	90	80	13	Hypertension Stage 1 (Systolic 130-139 Diastolic 80-89)
NIDDM763	43	Female	Over Weight	390	249	189	456	44	26	Hypertension Stage 2 (Systolic >140 Diastolic >90)
NIDDM764	44	Female	Over Weight	109	258	234	82	91	12	Hypertension Stage 2 (Systolic >140 Diastolic >90)
NIDDM765	42	Female	Obese	125	246	112	345	98	8	Hypertension Stage 2 (Systolic >140 Diastolic >90)
NIDDM766	47	Female	Over Weight	137	237	199	322	78	41	Hypertension Stage 1 (Systolic 130-139 Diastolic 80-89)
NIDDM767	55	Male	Over Weight	94	458	181	56	77	40	Hypertension Stage 1 (Systolic 130-139 Diastolic 80-89)
NIDDM768	56	Male	Normal	120	345	178	380	85	54	Hypertension Stage 1 (Systolic 130-139 Diastolic 80-89)
NIDDM769	58	Female	Obese	91	251	195	82	94	64	Hypertension Stage 1 (Systolic 130-139 Diastolic 80-89)
NIDDM770	43	Male	Obese	67	267	223	319	82	16	Hypertension Stage 1 (Systolic 130-139 Diastolic 80-89)
NIDDM771	54	Female	Obese	340	291	190	398	80	35	Hypertension Stage 1 (Systolic 130-139 Diastolic 80-89)
NIDDM772	43	Male	Obese	330	367	234	290	85	36	Hypertension Stage 1 (Systolic 130-139 Diastolic 80-89)
NIDDM773	60	Male	Over Weight	120	367	110	40	65	19	Hypertension Stage 1 (Systolic 130-139 Diastolic 80-89)
NIDDM774	61	Female	Normal	153	249	202	81	72	23	Hypertension Stage 1 (Systolic 130-139 Diastolic 80-89)
NIDDM775	51	Female	Normal	163	234	197	51	120	43	Hypertension Stage 1 (Systolic 130-139 Diastolic 80-89)
NIDDM776	39	Female	Obese	92	287	205	75	51	34	Hypertension Stage 1 (Systolic 130-139 Diastolic 80-89)
NIDDM777	59	Female	Normal	316	246	194	82	49	58	Hypertension Stage 1 (Systolic 130-139 Diastolic 80-89)
NIDDM778	61	Female	Over Weight	254	115	230	95	75	17	Hypertension Stage 1 (Systolic 130-139 Diastolic 80-89)
NIDDM779	58	Female	Normal	83	458	124	91	121	76	Hypertension Stage 1 (Systolic 130-139 Diastolic 80-89)
NIDDM780	54	Male	Normal	84	387	120	33	85	33	Hypertension Stage 1 (Systolic 130-139 Diastolic 80-89)
NIDDM781	47	Female	Over Weight	136	269	207	48	74	67	Hypertension Stage 1 (Systolic 130-139 Diastolic 80-89)
NIDDM782	47	Male	Over Weight	120	237	176	65	80	27	Hypertension Stage 1 (Systolic 130-139 Diastolic 80-89)
NIDDM783	56	Female	Over Weight	330	297	199	304	211	44	Hypertension Stage 1 (Systolic 130-139 Diastolic 80-89)
NIDDM784	60	Male	Normal	320	216	204	62	201	56	Hypertension Stage 1 (Systolic 130-139 Diastolic 80-89)
NIDDM785	43	Female	Normal	146	249	179	310	80	74	Hypertension Stage 1 (Systolic 130-139 Diastolic 80-89)
NIDDM786	57	Male	Over Weight	94	137	191	69	73	59	Hypertension Stage 1 (Systolic 130-139 Diastolic 80-89)
NIDDM787	57	Female	Normal	97	257	205	408	94	45	Hypertension Stage 1 (Systolic 130-139 Diastolic 80-89)
NIDDM788	46	Male	Normal	246	249	196	89	180	76	Hypertension Stage 1 (Systolic 130-139 Diastolic 80-89)
NIDDM789	60	Female	Over Weight	96	267	181	325	145	37	Hypertension Stage 1 (Systolic 130-139 Diastolic 80-89)
NIDDM790	52	Male	Normal	110	246	174	340	81	66	Hypertension Stage 2 (Systolic >140 Diastolic >90)
NIDDM791	61	Female	Over Weight	310	267	179	31	87	68	Hypertension Stage 1 (Systolic 130-139 Diastolic 80-89)
NIDDM792	53	Female	Over Weight	91	268	186	412	82	21	Hypertension Stage 1 (Systolic 130-139 Diastolic 80-89)
NIDDM793	54	Female	Normal	110	283	180	371	79	41	Hypertension Stage 1 (Systolic 130-139 Diastolic 80-89)
NIDDM794	48	Male	Normal	91	267	189	89	129	67	Hypertension Stage 1 (Systolic 130-139 Diastolic 80-89)
NIDDM795	42	Female	Normal	285	246	193	90	74	26	Hypertension Stage 1 (Systolic 130-139 Diastolic 80-89)
NIDDM796	49	Female	Normal	312	297	189	313	67	68	Hypertension Stage 1 (Systolic 130-139 Diastolic 80-89)
NIDDM797	49	Female	Normal	315	283	175	100	69	41	Hypertension Stage 1 (Systolic 130-139 Diastolic 80-89)
NIDDM798	54	Male	Normal	217	460	197	89	83	49	Hypertension Stage 1 (Systolic 130-139 Diastolic 80-89)
NIDDM799	57	Female	Normal	255	234	186	60	210	77	Hypertension Stage 1 (Systolic 130-139 Diastolic 80-89)
NIDDM800	50	Male	Over Weight	394	267	179	80	72	50	Hypertension Stage 1 (Systolic 130-139 Diastolic 80-89)
NIDDM801	42	Female	Over Weight	300	237	186	512	198	45	Hypertension Stage 1 (Systolic 130-139 Diastolic 80-89)
NIDDM802	44	Female	Over Weight	377	267	189	50	78	50	Hypertension Stage 1 (Systolic 130-139 Diastolic 80-89)
NIDDM803	59	Male	Normal	98	287	181	72	160	55	Hypertension Stage 1 (Systolic 130-139 Diastolic 80-89)
NIDDM804	58	Male	Over Weight	290	369	193	102	89	32	Hypertension Stage 1 (Systolic 130-139 Diastolic 80-89)
NIDDM805	41	Female	Over Weight	300	390	176	82	170	51	Hypertension Stage 1 (Systolic 130-139 Diastolic 80-89)
NIDDM806	52	Female	Over Weight	410	243	177	102	86	72	Hypertension Stage 2 (Systolic >140 Diastolic >90)
NIDDM807	55	Female	Over Weight	140	267	184	380	130	40	Hypertension Stage 1 (Systolic 130-139 Diastolic 80-89)
NIDDM808	41	Male	Normal	285	258	195	370	76	38	Hypertension Stage 1 (Systolic 130-139 Diastolic 80-89)
NIDDM809	57	Female	Over Weight	260	115	188	93	71	23	Hypertension Stage 1 (Systolic 130-139 Diastolic 80-89)
NIDDM810	52	Female	Normal	360	164	179	307	129	36	Hypertension Stage 1 (Systolic 130-139 Diastolic 80-89)
NIDDM811	46	Female	Over Weight	184	237	191	115	89	76	Hypertension Stage 1 (Systolic 130-139 Diastolic 80-89)
NIDDM812	53	Male	Over Weight	394	278	187	93	127	49	Hypertension Stage 1 (Systolic 130-139 Diastolic 80-89)
NIDDM813	46	Male	Normal	90	98	194	93	119	8	Hypertension Stage 1 (Systolic 130-139 Diastolic 80-89)
NIDDM814	60	Male	Over Weight	310	137	207	328	121	25	Hypertension Stage 1 (Systolic 130-139 Diastolic 80-89)
NIDDM815	41	Female	Over Weight	229	119	201	45	121	19	Hypertension Stage 1 (Systolic 130-139 Diastolic 80-89)
NIDDM816	43	Female	Over Weight	241	275	179	380	90	43	Hypertension Stage 1 (Systolic 130-139 Diastolic 80-89)
NIDDM817	59	Female	Over Weight	125	274	204	77	73	59	Hypertension Stage 2 (Systolic >140 Diastolic >90)
NIDDM818	41	Male	Over Weight	249	256	250	71	178	15	Hypertension Stage 1 (Systolic 130-139 Diastolic 80-89)
NIDDM819	51	Female	Over Weight	184	237	278	119	130	57	Hypertension Stage 1 (Systolic 130-139 Diastolic 80-89)
NIDDM820	39	Female	Normal	211	243	134	74	90	36	Hypertension Stage 1 (Systolic 130-139 Diastolic 80-89)
NIDDM821	39	Male	Over Weight	259	235	249	311	150	57	Hypertension Stage 1 (Systolic 130-139 Diastolic 80-89)
NIDDM822	42	Female	Over Weight	133	267	112	66	167	68	Hypertension Stage 1 (Systolic 130-139 Diastolic 80-89)
NIDDM823	60	Female	Over Weight	399	245	206	100	190	65	Hypertension Stage 1 (Systolic 130-139 Diastolic 80-89)
NIDDM824	43	Male	Normal	301	243	245	108	73	54	Hypertension Stage 1 (Systolic 130-139 Diastolic 80-89)
NIDDM825	40	Male	Normal	276	245	235	414	132	66	Hypertension Stage 2 (Systolic >140 Diastolic >90)
NIDDM826	41	Female	Normal	246	267	139	330	86	78	Hypertension Stage 2 (Systolic >140 Diastolic >90)
NIDDM827	60	Female	Normal	95	275	243	345	88	46	Hypertension Stage 2 (Systolic >140 Diastolic >90)
NIDDM828	47	Male	Normal	176	278	145	85	175	70	Hypertension Stage 2 (Systolic >140 Diastolic >90)
NIDDM829	43	Male	Normal	182	257	180	17	185	77	Hypertension Stage 1 (Systolic 130-139 Diastolic 80-89)
NIDDM830	39	Male	Over Weight	173	276	121	336	79	35	Hypertension Stage 1 (Systolic 130-139 Diastolic 80-89)
NIDDM831	41	Female	Over Weight	194	485	110	370	94	15	Hypertension Stage 1 (Systolic 130-139 Diastolic 80-89)
NIDDM832	49	Female	Normal	196	293	267	360	93	18	Hypertension Stage 1 (Systolic 130-139 Diastolic 80-89)
NIDDM833	56	Male	Over Weight	317	394	176	370	73	75	Hypertension Stage 1 (Systolic 130-139 Diastolic 80-89)
NIDDM834	58	Female	Over Weight	213	246	230	90	83	36	Hypertension Stage 1 (Systolic 130-139 Diastolic 80-89)
NIDDM835	46	Female	Normal	86	376	225	30	131	22	Hypertension Stage 1 (Systolic 130-139 Diastolic 80-89)
NIDDM836	44	Female	Over Weight	193	137	234	300	82	22	Hypertension Stage 1 (Systolic 130-139 Diastolic 80-89)
NIDDM837	46	Male	Over Weight	276	234	270	330	96	38	Hypertension Stage 1 (Systolic 130-139 Diastolic 80-89)

NIDDM838	41	Female	Normal	259	294	230	88	156	39	Hypertension Stage 2 (Systolic >140 Diastolic >90)
NIDDM839	23	Female	Over Weight	245	267	120	99	167	58	Hypertension Stage 1 (Systolic 130-139 Diastolic 80-89)
NIDDM840	29	Male	Over Weight	294	148	127	335	180	37	Hypertension Stage 1 (Systolic 130-139 Diastolic 80-89)
NIDDM841	26	Female	Normal	176	246	122	82	130	17	Hypertension Stage 1 (Systolic 130-139 Diastolic 80-89)
NIDDM842	45	Female	Over Weight	83	249	117	67	168	46	Hypertension Stage 1 (Systolic 130-139 Diastolic 80-89)
NIDDM843	67	Female	Over Weight	246	134	288	329	172	37	Hypertension Stage 1 (Systolic 130-139 Diastolic 80-89)
NIDDM844	65	Female	Over Weight	174	257	108	96	90	53	Hypertension Stage 2 (Systolic >140 Diastolic >90)
NIDDM845	45	Female	Over Weight	64	146	201	89	81	67	Hypertension Stage 2 (Systolic >140 Diastolic >90)
NIDDM846	42	Female	Over Weight	86	369	177	77	137	37	Hypertension Stage 2 (Systolic >140 Diastolic >90)
NIDDM847	58	Female	Over Weight	194	267	203	89	135	41	Hypertension Stage 2 (Systolic >140 Diastolic >90)
NIDDM848	60	Female	Over Weight	276	390	250	66	137	48	Hypertension Stage 2 (Systolic >140 Diastolic >90)
NIDDM849	41	Female	Over Weight	317	334	230	341	145	19	Hypertension Stage 2 (Systolic >140 Diastolic >90)
NIDDM850	48	Female	Over Weight	186	248	235	38	72	51	Hypertension Stage 2 (Systolic >140 Diastolic >90)
NIDDM851	61	Male	Normal	246	276	120	47	96	24	Hypertension Stage 2 (Systolic >140 Diastolic >90)
NIDDM852	49	Female	Over Weight	152	248	102	44	97	59	Hypertension Stage 2 (Systolic >140 Diastolic >90)
NIDDM853	52	Female	Over Weight	279	279	229	71	68	55	Hypertension Stage 2 (Systolic >140 Diastolic >90)
NIDDM854	51	Male	Over Weight	135	399	120	59	136	24	Hypertension Stage 2 (Systolic >140 Diastolic >90)
NIDDM855	52	Male	Over Weight	284	300	280	104	190	14	Hypertension Stage 2 (Systolic >140 Diastolic >90)
NIDDM856	48	Female	Over Weight	176	260	202	371	195	57	Hypertension Stage 2 (Systolic >140 Diastolic >90)
NIDDM857	48	Female	Over Weight	194	500	109	41	76	55	Hypertension Stage 2 (Systolic >140 Diastolic >90)
NIDDM858	52	Male	Normal	156	254	119	89	135	23	Hypertension Stage 2 (Systolic >140 Diastolic >90)
NIDDM859	43	Female	Over Weight	174	264	145	91	175	42	Hypertension Stage 2 (Systolic >140 Diastolic >90)
NIDDM860	56	Female	Over Weight	256	379	267	54	190	19	Hypertension Stage 2 (Systolic >140 Diastolic >90)
NIDDM861	42	Female	Over Weight	384	237	256	96	178	13	Hypertension Stage 2 (Systolic >140 Diastolic >90)
NIDDM862	50	Male	Over Weight	246	267	202	87	165	55	Hypertension Stage 2 (Systolic >140 Diastolic >90)
NIDDM863	55	Female	Over Weight	168	237	145	114	87	28	Hypertension Stage 2 (Systolic >140 Diastolic >90)
NIDDM864	42	Female	Normal	182	246	120	75	137	20	Hypertension Stage 2 (Systolic >140 Diastolic >90)
NIDDM865	42	Female	Over Weight	198	150	125	96	150	49	Hypertension Stage 2 (Systolic >140 Diastolic >90)
NIDDM866	62	Male	Over Weight	213	265	119	67	70	11	Hypertension Stage 2 (Systolic >140 Diastolic >90)
NIDDM867	41	Female	Over Weight	189	243	132	49	197	41	Hypertension Stage 2 (Systolic >140 Diastolic >90)
NIDDM868	50	Male	Over Weight	169	420	201	66	74	38	Hypertension Stage 2 (Systolic >140 Diastolic >90)
NIDDM869	44	Male	Normal	94	350	204	360	70	36	Hypertension Stage 2 (Systolic >140 Diastolic >90)
NIDDM870	48	Female	Over Weight	248	364	204	89	79	37	Hypertension Stage 2 (Systolic >140 Diastolic >90)
NIDDM871	43	Male	Over Weight	187	347	134	390	134	16	Hypertension Stage 2 (Systolic >140 Diastolic >90)
NIDDM872	61	Female	Over Weight	146	300	142	56	123	49	Hypertension Stage 2 (Systolic >140 Diastolic >90)
NIDDM873	39	Male	Over Weight	250	416	191	34	134	45	Hypertension Stage 2 (Systolic >140 Diastolic >90)
NIDDM874	47	Male	Over Weight	194	480	230	367	138	27	Hypertension Stage 2 (Systolic >140 Diastolic >90)
NIDDM875	48	Female	Over Weight	149	348	130	330	96	27	Hypertension Stage 2 (Systolic >140 Diastolic >90)
NIDDM876	61	Male	Over Weight	164	347	267	78	188	63	Hypertension Stage 2 (Systolic >140 Diastolic >90)
NIDDM877	39	Male	Normal	159	348	116	89	93	36	Hypertension Stage 2 (Systolic >140 Diastolic >90)
NIDDM878	58	Female	Over Weight	123	294	203	54	178	43	Hypertension Stage 2 (Systolic >140 Diastolic >90)
NIDDM879	49	Female	Over Weight	221	300	240	102	176	23	Hypertension Stage 2 (Systolic >140 Diastolic >90)
NIDDM880	60	Female	Over Weight	76	300	203	328	190	20	Hypertension Stage 2 (Systolic >140 Diastolic >90)
NIDDM881	61	Male	Over Weight	246	350	230	65	165	62	Hypertension Stage 2 (Systolic >140 Diastolic >90)
NIDDM882	60	Female	Over Weight	164	324	190	62	133	54	Hypertension Stage 2 (Systolic >140 Diastolic >90)
NIDDM883	57	Female	Normal	182	357	193	47	195	11	Hypertension Stage 2 (Systolic >140 Diastolic >90)
NIDDM884	44	Male	Normal	283	324	202	72	74	61	Hypertension Stage 2 (Systolic >140 Diastolic >90)
NIDDM885	65	Male	Normal	246	410	130	90	83	44	Hypertension Stage 2 (Systolic >140 Diastolic >90)
NIDDM886	34	Female	Normal	176	314	125	380	87	32	Hypertension Stage 2 (Systolic >140 Diastolic >90)
NIDDM887	35	Female	Normal	159	374	230	345	70	58	Hypertension Stage 2 (Systolic >140 Diastolic >90)
NIDDM888	65	Female	Normal	276	420	232	89	87	64	Hypertension Stage 2 (Systolic >140 Diastolic >90)
NIDDM889	68	Male	Normal	245	248	150	350	80	61	Hypertension Stage 2 (Systolic >140 Diastolic >90)
NIDDM890	72	Female	Normal	296	321	190	80	185	44	Hypertension Stage 2 (Systolic >140 Diastolic >90)
NIDDM891	32	Female	Over Weight	192	350	204	348	90	54	Hypertension Stage 2 (Systolic >140 Diastolic >90)
NIDDM892	28	Female	Normal	175	348	173	300	67	50	Hypertension Stage 2 (Systolic >140 Diastolic >90)
NIDDM893	34	Male	Over Weight	176	424	178	126	74	11	Hypertension Stage 2 (Systolic >140 Diastolic >90)
NIDDM894	39	Male	Over Weight	198	158	163	70	130	46	Hypertension Stage 2 (Systolic >140 Diastolic >90)
NIDDM895	73	Female	Normal	168	341	207	310	178	53	Hypertension Stage 2 (Systolic >140 Diastolic >90)
NIDDM896	28	Female	Over Weight	298	334	191	71	65	59	Hypertension Stage 2 (Systolic >140 Diastolic >90)
NIDDM897	69	Male	Normal	182	324	112	105	87	32	Hypertension Stage 2 (Systolic >140 Diastolic >90)
NIDDM898	24	Female	Over Weight	369	410	117	122	96	62	Hypertension Stage 2 (Systolic >140 Diastolic >90)
NIDDM899	67	Female	Normal	69	311	198	400	87	41	Hypertension Stage 2 (Systolic >140 Diastolic >90)
NIDDM900	70	Male	Normal	241	324	250	393	93	66	Hypertension Stage 2 (Systolic >140 Diastolic >90)
NIDDM901	24	Female	Normal	195	398	169	403	126	14	Hypertension Stage 2 (Systolic >140 Diastolic >90)
NIDDM902	64	Female	Over Weight	188	346	207	89	82	59	Hypertension Stage 2 (Systolic >140 Diastolic >90)
NIDDM903	60	Male	Normal	228	350	180	126	78	24	Hypertension Stage 2 (Systolic >140 Diastolic >90)
NIDDM904	61	Female	Over Weight	183	267	177	80	71	13	Hypertension Stage 2 (Systolic >140 Diastolic >90)
NIDDM905	45	Male	Normal	85	340	192	394	45	68	Hypertension Stage 2 (Systolic >140 Diastolic >90)
NIDDM906	41	Female	Normal	195	333	211	66	138	46	Hypertension Stage 2 (Systolic >140 Diastolic >90)
NIDDM907	57	Female	Over Weight	164	341	179	310	86	8	Hypertension Stage 2 (Systolic >140 Diastolic >90)
NIDDM908	49	Female	Normal	183	427	167	357	129	62	Hypertension Stage 2 (Systolic >140 Diastolic >90)
NIDDM909	40	Male	Normal	194	320	201	390	51	17	Hypertension Stage 2 (Systolic >140 Diastolic >90)
NIDDM910	43	Female	Over Weight	163	245	185	390	139	23	Hypertension Stage 2 (Systolic >140 Diastolic >90)
NIDDM911	40	Male	Normal	213	100	173	103	87	31	Hypertension Stage 2 (Systolic >140 Diastolic >90)
NIDDM912	46	Female	Over Weight	194	410	134	87	94	11	Hypertension Stage 2 (Systolic >140 Diastolic >90)
NIDDM913	58	Female	Normal	153	257	196	315	82	51	Hypertension Stage 2 (Systolic >140 Diastolic >90)
NIDDM914	49	Male	Normal	134	357	122	60	50	35	Hypertension Stage 2 (Systolic >140 Diastolic >90)
NIDDM915	42	Female	Normal	266	400	170	125	99	40	Hypertension Stage 2 (Systolic >140 Diastolic >90)
NIDDM916	42	Male	Normal	179	248	203	345	130	55	Hypertension Stage 2 (Systolic >140 Diastolic >90)
NIDDM917	46	Female	Over Weight	108	368	191	389	87	17	Hypertension Stage 2 (Systolic >140 Diastolic >90)
NIDDM918	58	Male	Normal	209	323	205	376	125	78	Hypertension Stage 2 (Systolic >140 Diastolic >90)
NIDDM919	43	Female	Over Weight	224	498	175	90	94	74	Hypertension Stage 2 (Systolic >140 Diastolic >90)
NIDDM920	51	Female	Over Weight	192	300	179	105	72	58	Hypertension Stage 2 (Systolic >140 Diastolic >90)
NIDDM921	59	Female	Normal	295	397	178	105	136	43	Hypertension Stage 2 (Systolic >140 Diastolic >90)

NIDDM922	54	Female	Over Weight	176	448	196	108	99	21	Hypertension Stage 2 (Systolic >140 Diastolic >90)
NIDDM923	54	Male	Over Weight	269	367	187	114	82	59	Hypertension Stage 2 (Systolic >140 Diastolic >90)
NIDDM924	51	Female	Over Weight	184	247	199	77	70	17	Hypertension Stage 2 (Systolic >140 Diastolic >90)
NIDDM925	51	Male	Over Weight	189	267	208	88	131	78	Hypertension Stage 2 (Systolic >140 Diastolic >90)
NIDDM926	59	Female	Normal	241	126	205	321	101	68	Hypertension Stage 2 (Systolic >140 Diastolic >90)
NIDDM927	55	Female	Over Weight	251	320	180	80	110	50	Hypertension Stage 2 (Systolic >140 Diastolic >90)
NIDDM928	40	Female	Normal	158	487	200	360	120	20	Hypertension Stage 2 (Systolic >140 Diastolic >90)
NIDDM929	51	Female	Normal	94	267	198	321	113	63	Hypertension Stage 2 (Systolic >140 Diastolic >90)
NIDDM930	55	Female	Over Weight	223	237	187	47	91	18	Hypertension Stage 2 (Systolic >140 Diastolic >90)
NIDDM931	57	Female	Normal	165	248	186	89	80	45	Hypertension Stage 2 (Systolic >140 Diastolic >90)
NIDDM932	50	Female	Over Weight	283	457	171	88	52	29	Hypertension Stage 2 (Systolic >140 Diastolic >90)
NIDDM933	49	Male	Over Weight	286	497	201	54	76	29	Hypertension Stage 2 (Systolic >140 Diastolic >90)
NIDDM934	39	Female	Normal	214	467	199	137	81	50	Hypertension Stage 2 (Systolic >140 Diastolic >90)
NIDDM935	40	Female	Normal	155	487	181	34	72	47	Hypertension Stage 2 (Systolic >140 Diastolic >90)
NIDDM936	46	Female	Normal	198	364	190	44	90	18	Hypertension Stage 2 (Systolic >140 Diastolic >90)
NIDDM937	45	Female	Normal	176	378	196	69	72	54	Hypertension Stage 2 (Systolic >140 Diastolic >90)
NIDDM938	60	Female	Normal	156	369	130	57	100	64	Hypertension Stage 2 (Systolic >140 Diastolic >90)
NIDDM939	42	Female	Normal	246	397	105	89	69	51	Hypertension Stage 2 (Systolic >140 Diastolic >90)
NIDDM940	61	Female	Over Weight	259	348	128	302	75	66	Hypertension Stage 2 (Systolic >140 Diastolic >90)
NIDDM941	56	Female	Normal	294	245	207	401	134	45	Hypertension Stage 2 (Systolic >140 Diastolic >90)
NIDDM942	44	Male	Over Weight	158	387	183	312	83	43	Hypertension Stage 2 (Systolic >140 Diastolic >90)
NIDDM943	56	Female	Normal	176	264	175	345	112	11	Hypertension Stage 2 (Systolic >140 Diastolic >90)
NIDDM944	42	Male	Normal	198	287	178	355	93	38	Hypertension Stage 2 (Systolic >140 Diastolic >90)
NIDDM945	39	Female	Normal	148	249	196	371	79	23	Hypertension Stage 2 (Systolic >140 Diastolic >90)
NIDDM946	55	Female	Normal	146	347	240	59	73	53	Hypertension Stage 2 (Systolic >140 Diastolic >90)
NIDDM947	30	Female	Over Weight	152	267	135	70	119	41	Hypertension Stage 2 (Systolic >140 Diastolic >90)
NIDDM948	29	Female	Normal	113	252	191	390	81	11	Hypertension Stage 2 (Systolic >140 Diastolic >90)
NIDDM949	33	Female	Normal	126	267	204	94	78	14	Hypertension Stage 2 (Systolic >140 Diastolic >90)
NIDDM950	25	Female	Over Weight	88	249	110	308	80	24	Hypertension Stage 2 (Systolic >140 Diastolic >90)
NIDDM951	28	Male	Normal	184	237	198	345	74	45	Hypertension Stage 2 (Systolic >140 Diastolic >90)
NIDDM952	25	Female	Normal	111	123	190	319	95	49	Hypertension Stage 2 (Systolic >140 Diastolic >90)
NIDDM953	31	Male	Normal	78	300	140	360	78	60	Hypertension Stage 2 (Systolic >140 Diastolic >90)
NIDDM954	37	Female	Over Weight	102	245	192	100	70	20	Hypertension Stage 2 (Systolic >140 Diastolic >90)
NIDDM955	68	Male	Over Weight	133	367	186	67	99	20	Hypertension Stage 2 (Systolic >140 Diastolic >90)
NIDDM956	30	Female	Over Weight	245	351	265	400	90	51	Hypertension Stage 2 (Systolic >140 Diastolic >90)
NIDDM957	36	Female	Over Weight	255	324	184	322	97	50	Hypertension Stage 2 (Systolic >140 Diastolic >90)
NIDDM958	63	Female	Normal	156	367	199	367	100	35	Hypertension Stage 2 (Systolic >140 Diastolic >90)
NIDDM959	64	Male	Normal	158	397	187	63	130	25	Hypertension Stage 2 (Systolic >140 Diastolic >90)
NIDDM960	39	Female	Normal	199	265	175	390	119	40	Hypertension Stage 2 (Systolic >140 Diastolic >90)
NIDDM961	71	Male	Over Weight	129	397	203	310	67	47	Hypertension Stage 2 (Systolic >140 Diastolic >90)
NIDDM962	70	Male	Normal	362	330	132	345	90	27	Hypertension Stage 2 (Systolic >140 Diastolic >90)
NIDDM963	31	Female	Over Weight	258	124	192	72	91	29	Hypertension Stage 2 (Systolic >140 Diastolic >90)
NIDDM964	62	Female	Normal	236	369	235	348	111	76	Hypertension Stage 2 (Systolic >140 Diastolic >90)
NIDDM965	32	Male	Normal	169	241	201	329	90	22	Hypertension Stage 2 (Systolic >140 Diastolic >90)
NIDDM966	64	Female	Normal	150	367	123	75	101	51	Hypertension Stage 2 (Systolic >140 Diastolic >90)
NIDDM967	31	Female	Normal	231	327	265	377	125	34	Hypertension Stage 2 (Systolic >140 Diastolic >90)
NIDDM968	29	Female	Normal	152	304	112	72	67	42	Hypertension Stage 2 (Systolic >140 Diastolic >90)
NIDDM969	63	Male	Normal	370	317	280	410	119	53	Hypertension Stage 2 (Systolic >140 Diastolic >90)
NIDDM970	75	Female	Normal	205	324	234	86	136	11	Hypertension Stage 2 (Systolic >140 Diastolic >90)
NIDDM971	29	Female	Over Weight	145	124	194	58	118	65	Hypertension Stage 2 (Systolic >140 Diastolic >90)
NIDDM972	27	Male	Normal	165	297	123	49	131	33	Hypertension Stage 2 (Systolic >140 Diastolic >90)
NIDDM973	43	Female	Normal	169	267	176	390	101	64	Hypertension Stage 2 (Systolic >140 Diastolic >90)
NIDDM974	78	Female	Normal	142	118	113	59	69	45	Hypertension Stage 2 (Systolic >140 Diastolic >90)
NIDDM975	42	Female	Over Weight	102	147	180	61	103	39	Hypertension Stage 2 (Systolic >140 Diastolic >90)
NIDDM976	61	Female	Over Weight	80	374	176	339	86	50	Hypertension Stage 2 (Systolic >140 Diastolic >90)
NIDDM977	59	Female	Over Weight	110	254	123	63	126	45	Hypertension Stage 2 (Systolic >140 Diastolic >90)
NIDDM978	59	Male	Over Weight	125	264	177	90	107	46	Hypertension Stage 2 (Systolic >140 Diastolic >90)
NIDDM979	44	Female	Normal	80	147	191	390	96	43	Hypertension Stage 2 (Systolic >140 Diastolic >90)
NIDDM980	43	Male	Over Weight	150	249	142	67	129	42	Hypertension Stage 2 (Systolic >140 Diastolic >90)
NIDDM981	58	Male	Normal	100	247	243	345	83	36	Hypertension Stage 2 (Systolic >140 Diastolic >90)
NIDDM982	45	Female	Over Weight	100	300	195	385	77	48	Hypertension Stage 2 (Systolic >140 Diastolic >90)
NIDDM983	41	Female	Over Weight	90	150	180	81	85	46	Hypertension Stage 2 (Systolic >140 Diastolic >90)
NIDDM984	54	Male	Over Weight	100	118	121	68	105	46	Hypertension Stage 2 (Systolic >140 Diastolic >90)
NIDDM985	62	Male	Over Weight	80	98	145	304	117	57	Hypertension Stage 2 (Systolic >140 Diastolic >90)
NIDDM986	53	Female	Normal	70	367	203	341	114	11	Hypertension Stage 2 (Systolic >140 Diastolic >90)
NIDDM987	59	Male	Over Weight	232	100	112	54	121	19	Hypertension Stage 2 (Systolic >140 Diastolic >90)
NIDDM988	61	Female	Over Weight	180	300	195	313	110	58	Hypertension Stage 2 (Systolic >140 Diastolic >90)
NIDDM989	43	Male	Over Weight	69	367	237	400	128	10	Hypertension Stage 2 (Systolic >140 Diastolic >90)
NIDDM990	45	Female	Over Weight	125	395	132	333	116	56	Hypertension Stage 2 (Systolic >140 Diastolic >90)
NIDDM991	51	Male	Normal	156	367	187	67	137	51	Hypertension Stage 2 (Systolic >140 Diastolic >90)
NIDDM992	58	Male	Over Weight	236	316	203	93	136	54	Hypertension Stage 2 (Systolic >140 Diastolic >90)
NIDDM993	41	Female	Over Weight	158	349	178	80	132	25	Hypertension Stage 2 (Systolic >140 Diastolic >90)
NIDDM994	48	Male	Normal	136	327	197	84	68	14	Hypertension Stage 2 (Systolic >140 Diastolic >90)
NIDDM995	42	Female	Normal	125	376	123	90	130	38	Hypertension Stage 2 (Systolic >140 Diastolic >90)
NIDDM996	40	Female	Normal	96	248	120	45	98	17	Hypertension Stage 2 (Systolic >140 Diastolic >90)
NIDDM997	49	Male	Normal	125	367	177	34	115	42	Hypertension Stage 2 (Systolic >140 Diastolic >90)
NIDDM998	60	Male	Over Weight	405	397	141	435	95	31	Hypertension Stage 2 (Systolic >140 Diastolic >90)
NIDDM999	54	Male	Over Weight	360	348	194	142	68	21	Hypertension Stage 2 (Systolic >140 Diastolic >90)
NIDDM1000	39	Female	Over Weight	159	400	123	402	133	35	Hypertension Stage 2 (Systolic >140 Diastolic >90)
NIDDM1001	51	Female	Normal	132	500	190	380	114	46	Hypertension Stage 2 (Systolic >140 Diastolic >90)
NIDDM1002	55	Female	Over Weight	101	397	265	49	120	26	Hypertension Stage 2 (Systolic >140 Diastolic >90)
NIDDM1003	57	Male	Over Weight	400	148	185	82	94	20	Hypertension Stage 2 (Systolic >140 Diastolic >90)
NIDDM1004	62	Male	Over Weight	185	365	244	41	87	29	Hypertension Stage 2 (Systolic >140 Diastolic >90)
NIDDM1005	51	Female	Normal	125	124	190	40	122	25	Hypertension Stage 2 (Systolic >140 Diastolic >90)
