# Supplementary material for: Linkage disequilibrium block single-nucleotide polymorphisms in FTO alpha ketoglutarate dependent dioxygenase gene inference with breast cancer and Type II diabetes in Pakistani female population
Source: PLoS One. 2023 Jul 20;18(7):e0288934. doi: 10.1371/journal.pone.0288934 (PMC10358933; doi:10.1371/journal.pone.0288934)
Supplement: S4 File — (PDF) [file pone.0288934.s004.pdf]

S4: Minimal Data Set for Type II diabetes Controls

Sample ID	Age in Years	Gender	Hypertension
CNIDDM001	42	Male	Elevated (Systolic 120-129 Diastolic <80)
CNIDDM002	64	Female	Elevated (Systolic 120-129 Diastolic <80)
CNIDDM003	56	Female	Elevated (Systolic 120-129 Diastolic <80)
CNIDDM004	43	Female	Elevated (Systolic 120-129 Diastolic <80)
CNIDDM005	44	Female	Elevated (Systolic 120-129 Diastolic <80)
CNIDDM006	42	Male	Elevated (Systolic 120-129 Diastolic <80)
CNIDDM007	45	Male	Elevated (Systolic 120-129 Diastolic <80)
CNIDDM008	41	Female	Elevated (Systolic 120-129 Diastolic <80)
CNIDDM009	52	Female	Elevated (Systolic 120-129 Diastolic <80)
CNIDDM010	48	Female	Elevated (Systolic 120-129 Diastolic <80)
CNIDDM011	65	Female	Elevated (Systolic 120-129 Diastolic <80)
CNIDDM012	71	Male	Elevated (Systolic 120-129 Diastolic <80)
CNIDDM013	40	Male	Elevated (Systolic 120-129 Diastolic <80)
CNIDDM014	41	Female	Elevated (Systolic 120-129 Diastolic <80)
CNIDDM015	50	Female	Elevated (Systolic 120-129 Diastolic <80)
CNIDDM016	43	Female	Elevated (Systolic 120-129 Diastolic <80)
CNIDDM017	70	Male	Elevated (Systolic 120-129 Diastolic <80)
CNIDDM018	43	Female	Elevated (Systolic 120-129 Diastolic <80)
CNIDDM019	42	Male	Elevated (Systolic 120-129 Diastolic <80)
CNIDDM020	36	Female	Elevated (Systolic 120-129 Diastolic <80)
CNIDDM021	52	Female	Elevated (Systolic 120-129 Diastolic <80)
CNIDDM022	63	Female	Elevated (Systolic 120-129 Diastolic <80)
CNIDDM023	56	Male	Elevated (Systolic 120-129 Diastolic <80)
CNIDDM024	30	Female	Elevated (Systolic 120-129 Diastolic <80)
CNIDDM025	61	Female	Elevated (Systolic 120-129 Diastolic <80)
CNIDDM026	43	Female	Elevated (Systolic 120-129 Diastolic <80)
CNIDDM027	49	Male	Elevated (Systolic 120-129 Diastolic <80)
CNIDDM028	57	Female	Elevated (Systolic 120-129 Diastolic <80)
CNIDDM029	48	Female	Elevated (Systolic 120-129 Diastolic <80)
CNIDDM030	41	Female	Elevated (Systolic 120-129 Diastolic <80)
CNIDDM031	50	Female	Elevated (Systolic 120-129 Diastolic <80)
CNIDDM032	61	Male	Elevated (Systolic 120-129 Diastolic <80)
CNIDDM033	50	Male	Elevated (Systolic 120-129 Diastolic <80)
CNIDDM034	57	Female	Elevated (Systolic 120-129 Diastolic <80)
CNIDDM035	60	Female	Elevated (Systolic 120-129 Diastolic <80)
CNIDDM036	42	Female	Elevated (Systolic 120-129 Diastolic <80)
CNIDDM037	50	Male	Elevated (Systolic 120-129 Diastolic <80)
CNIDDM038	65	Female	Elevated (Systolic 120-129 Diastolic <80)
CNIDDM039	54	Female	Elevated (Systolic 120-129 Diastolic <80)
CNIDDM040	54	Female	Elevated (Systolic 120-129 Diastolic <80)
CNIDDM041	71	Female	Elevated (Systolic 120-129 Diastolic <80)
CNIDDM042	50	Female	Elevated (Systolic 120-129 Diastolic <80)
CNIDDM043	57	Female	Elevated (Systolic 120-129 Diastolic <80)
CNIDDM044	67	Female	Elevated (Systolic 120-129 Diastolic <80)

CNIDDM045	50	Female	Elevated (Systolic 120-129 Diastolic <80)
CNIDDM046	41	Female	Elevated (Systolic 120-129 Diastolic <80)
CNIDDM047	42	Female	Elevated (Systolic 120-129 Diastolic <80)
CNIDDM048	49	Female	Elevated (Systolic 120-129 Diastolic <80)
CNIDDM049	67	Female	Elevated (Systolic 120-129 Diastolic <80)
CNIDDM050	49	Female	Elevated (Systolic 120-129 Diastolic <80)
CNIDDM051	72	Male	Elevated (Systolic 120-129 Diastolic <80)
CNIDDM052	57	Male	Elevated (Systolic 120-129 Diastolic <80)
CNIDDM053	65	Female	Elevated (Systolic 120-129 Diastolic <80)
CNIDDM054	60	Female	Elevated (Systolic 120-129 Diastolic <80)
CNIDDM055	55	Female	Elevated (Systolic 120-129 Diastolic <80)
CNIDDM056	38	Female	Elevated (Systolic 120-129 Diastolic <80)
CNIDDM057	51	Male	Elevated (Systolic 120-129 Diastolic <80)
CNIDDM058	41	Female	Elevated (Systolic 120-129 Diastolic <80)
CNIDDM059	52	Female	Elevated (Systolic 120-129 Diastolic <80)
CNIDDM060	62	Female	Elevated (Systolic 120-129 Diastolic <80)
CNIDDM061	67	Female	Elevated (Systolic 120-129 Diastolic <80)
CNIDDM062	29	Female	Elevated (Systolic 120-129 Diastolic <80)
CNIDDM063	30	Female	Elevated (Systolic 120-129 Diastolic <80)
CNIDDM064	46	Female	Elevated (Systolic 120-129 Diastolic <80)
CNIDDM065	63	Female	Elevated (Systolic 120-129 Diastolic <80)
CNIDDM066	29	Male	Elevated (Systolic 120-129 Diastolic <80)
CNIDDM067	61	Male	Elevated (Systolic 120-129 Diastolic <80)
CNIDDM068	46	Female	Elevated (Systolic 120-129 Diastolic <80)
CNIDDM069	39	Female	Elevated (Systolic 120-129 Diastolic <80)
CNIDDM070	40	Male	Elevated (Systolic 120-129 Diastolic <80)
CNIDDM071	55	Male	Elevated (Systolic 120-129 Diastolic <80)
CNIDDM072	40	Female	Normal (Systolic < 120 Diastolic < 80)
CNIDDM073	37	Male	Normal (Systolic < 120 Diastolic < 80)
CNIDDM074	67	Female	Normal (Systolic < 120 Diastolic < 80)
CNIDDM075	27	Male	Normal (Systolic < 120 Diastolic < 80)
CNIDDM076	46	Female	Normal (Systolic < 120 Diastolic < 80)
CNIDDM077	51	Female	Normal (Systolic < 120 Diastolic < 80)
CNIDDM078	59	Female	Normal (Systolic < 120 Diastolic < 80)
CNIDDM079	45	Male	Normal (Systolic < 120 Diastolic < 80)
CNIDDM080	25	Female	Normal (Systolic < 120 Diastolic < 80)
CNIDDM081	40	Male	Normal (Systolic < 120 Diastolic < 80)
CNIDDM082	43	Female	Normal (Systolic < 120 Diastolic < 80)
CNIDDM083	54	Female	Normal (Systolic < 120 Diastolic < 80)
CNIDDM084	39	Female	Normal (Systolic < 120 Diastolic < 80)
CNIDDM085	46	Male	Normal (Systolic < 120 Diastolic < 80)
CNIDDM086	41	Female	Normal (Systolic < 120 Diastolic < 80)
CNIDDM087	45	Female	Normal (Systolic < 120 Diastolic < 80)
CNIDDM088	53	Female	Normal (Systolic < 120 Diastolic < 80)
CNIDDM089	43	Female	Normal (Systolic < 120 Diastolic < 80)
CNIDDM090	65	Female	Normal (Systolic < 120 Diastolic < 80)
CNIDDM091	40	Female	Normal (Systolic < 120 Diastolic < 80)

CNIDDM092	62	Female	Normal (Systolic < 120 Diastolic < 80)
CNIDDM093	57	Male	Normal (Systolic < 120 Diastolic < 80)
CNIDDM094	37	Male	Normal (Systolic < 120 Diastolic < 80)
CNIDDM095	44	Female	Normal (Systolic < 120 Diastolic < 80)
CNIDDM096	55	Male	Normal (Systolic < 120 Diastolic < 80)
CNIDDM097	42	Female	Normal (Systolic < 120 Diastolic < 80)
CNIDDM098	44	Female	Normal (Systolic < 120 Diastolic < 80)
CNIDDM099	51	Female	Normal (Systolic < 120 Diastolic < 80)
CNIDDM100	67	Female	Normal (Systolic < 120 Diastolic < 80)
CNIDDM101	46	Female	Normal (Systolic < 120 Diastolic < 80)
CNIDDM102	42	Female	Normal (Systolic < 120 Diastolic < 80)
CNIDDM103	39	Female	Normal (Systolic < 120 Diastolic < 80)
CNIDDM104	42	Female	Normal (Systolic < 120 Diastolic < 80)
CNIDDM105	53	Female	Normal (Systolic < 120 Diastolic < 80)
CNIDDM106	45	Male	Normal (Systolic < 120 Diastolic < 80)
CNIDDM107	43	Male	Normal (Systolic < 120 Diastolic < 80)
CNIDDM108	42	Female	Normal (Systolic < 120 Diastolic < 80)
CNIDDM109	45	Female	Normal (Systolic < 120 Diastolic < 80)
CNIDDM110	40	Female	Normal (Systolic < 120 Diastolic < 80)
CNIDDM111	71	Female	Normal (Systolic < 120 Diastolic < 80)
CNIDDM112	39	Female	Normal (Systolic < 120 Diastolic < 80)
CNIDDM113	41	Male	Normal (Systolic < 120 Diastolic < 80)
CNIDDM114	46	Female	Normal (Systolic < 120 Diastolic < 80)
CNIDDM115	44	Female	Normal (Systolic < 120 Diastolic < 80)
CNIDDM116	55	Male	Normal (Systolic < 120 Diastolic < 80)
CNIDDM117	43	Female	Normal (Systolic < 120 Diastolic < 80)
CNIDDM118	37	Female	Normal (Systolic < 120 Diastolic < 80)
CNIDDM119	63	Female	Normal (Systolic < 120 Diastolic < 80)
CNIDDM120	54	Female	Normal (Systolic < 120 Diastolic < 80)
CNIDDM121	64	Female	Normal (Systolic < 120 Diastolic < 80)
CNIDDM122	43	Female	Normal (Systolic < 120 Diastolic < 80)
CNIDDM123	46	Male	Normal (Systolic < 120 Diastolic < 80)
CNIDDM124	37	Female	Normal (Systolic < 120 Diastolic < 80)
CNIDDM125	44	Male	Normal (Systolic < 120 Diastolic < 80)
CNIDDM126	43	Female	Normal (Systolic < 120 Diastolic < 80)
CNIDDM127	45	Female	Normal (Systolic < 120 Diastolic < 80)
CNIDDM128	50	Male	Normal (Systolic < 120 Diastolic < 80)
CNIDDM129	53	Female	Normal (Systolic < 120 Diastolic < 80)
CNIDDM130	54	Male	Normal (Systolic < 120 Diastolic < 80)
CNIDDM131	43	Male	Normal (Systolic < 120 Diastolic < 80)
CNIDDM132	55	Female	Normal (Systolic < 120 Diastolic < 80)
CNIDDM133	46	Male	Normal (Systolic < 120 Diastolic < 80)
CNIDDM134	45	Female	Normal (Systolic < 120 Diastolic < 80)
CNIDDM135	37	Female	Normal (Systolic < 120 Diastolic < 80)
CNIDDM136	63	Female	Normal (Systolic < 120 Diastolic < 80)
CNIDDM137	56	Male	Normal (Systolic < 120 Diastolic < 80)
CNIDDM138	45	Female	Normal (Systolic < 120 Diastolic < 80)

CNIDDM139	50	Female	Normal (Systolic < 120 Diastolic < 80)
CNIDDM140	65	Male	Normal (Systolic < 120 Diastolic < 80)
CNIDDM141	61	Female	Normal (Systolic < 120 Diastolic < 80)
CNIDDM142	37	Female	Normal (Systolic < 120 Diastolic < 80)
CNIDDM143	45	Female	Normal (Systolic < 120 Diastolic < 80)
CNIDDM144	36	Male	Normal (Systolic < 120 Diastolic < 80)
CNIDDM145	61	Female	Normal (Systolic < 120 Diastolic < 80)
CNIDDM146	54	Female	Normal (Systolic < 120 Diastolic < 80)
CNIDDM147	55	Female	Normal (Systolic < 120 Diastolic < 80)
CNIDDM148	54	Male	Normal (Systolic < 120 Diastolic < 80)
CNIDDM149	60	Female	Normal (Systolic < 120 Diastolic < 80)
CNIDDM150	55	Female	Normal (Systolic < 120 Diastolic < 80)
CNIDDM151	60	Male	Normal (Systolic < 120 Diastolic < 80)
CNIDDM152	37	Female	Normal (Systolic < 120 Diastolic < 80)
CNIDDM153	42	Male	Normal (Systolic < 120 Diastolic < 80)
CNIDDM154	51	Female	Normal (Systolic < 120 Diastolic < 80)
CNIDDM155	42	Female	Normal (Systolic < 120 Diastolic < 80)
CNIDDM156	56	Female	Normal (Systolic < 120 Diastolic < 80)
CNIDDM157	44	Female	Normal (Systolic < 120 Diastolic < 80)
CNIDDM158	55	Female	Normal (Systolic < 120 Diastolic < 80)
CNIDDM159	58	Female	Normal (Systolic < 120 Diastolic < 80)
CNIDDM160	42	Male	Normal (Systolic < 120 Diastolic < 80)
CNIDDM161	55	Female	Normal (Systolic < 120 Diastolic < 80)
CNIDDM162	40	Male	Normal (Systolic < 120 Diastolic < 80)
CNIDDM163	46	Female	Normal (Systolic < 120 Diastolic < 80)
CNIDDM164	46	Female	Normal (Systolic < 120 Diastolic < 80)
CNIDDM165	56	Male	Normal (Systolic < 120 Diastolic < 80)
CNIDDM166	42	Female	Normal (Systolic < 120 Diastolic < 80)
CNIDDM167	36	Female	Normal (Systolic < 120 Diastolic < 80)
CNIDDM168	37	Female	Normal (Systolic < 120 Diastolic < 80)
CNIDDM169	52	Female	Normal (Systolic < 120 Diastolic < 80)
CNIDDM170	60	Female	Normal (Systolic < 120 Diastolic < 80)
CNIDDM171	41	Male	Normal (Systolic < 120 Diastolic < 80)
CNIDDM172	60	Female	Normal (Systolic < 120 Diastolic < 80)
CNIDDM173	54	Female	Normal (Systolic < 120 Diastolic < 80)
CNIDDM174	43	Female	Normal (Systolic < 120 Diastolic < 80)
CNIDDM175	41	Female	Normal (Systolic < 120 Diastolic < 80)
CNIDDM176	46	Female	Normal (Systolic < 120 Diastolic < 80)
CNIDDM177	38	Male	Normal (Systolic < 120 Diastolic < 80)
CNIDDM178	50	Female	Normal (Systolic < 120 Diastolic < 80)
CNIDDM179	38	Male	Normal (Systolic < 120 Diastolic < 80)
CNIDDM180	60	Female	Normal (Systolic < 120 Diastolic < 80)
CNIDDM181	39	Female	Normal (Systolic < 120 Diastolic < 80)
CNIDDM182	60	Female	Normal (Systolic < 120 Diastolic < 80)
CNIDDM183	40	Female	Normal (Systolic < 120 Diastolic < 80)
CNIDDM184	37	Female	Normal (Systolic < 120 Diastolic < 80)
CNIDDM185	61	Female	Normal (Systolic < 120 Diastolic < 80)

CNIDDM186	38	Female	Normal (Systolic < 120 Diastolic < 80)
CNIDDM187	61	Female	Normal (Systolic < 120 Diastolic < 80)
CNIDDM188	46	Female	Normal (Systolic < 120 Diastolic < 80)
CNIDDM189	38	Male	Normal (Systolic < 120 Diastolic < 80)
CNIDDM190	37	Female	Normal (Systolic < 120 Diastolic < 80)
CNIDDM191	57	Female	Normal (Systolic < 120 Diastolic < 80)
CNIDDM192	50	Female	Normal (Systolic < 120 Diastolic < 80)
CNIDDM193	38	Female	Normal (Systolic < 120 Diastolic < 80)
CNIDDM194	43	Female	Normal (Systolic < 120 Diastolic < 80)
CNIDDM195	37	Female	Normal (Systolic < 120 Diastolic < 80)
CNIDDM196	43	Male	Normal (Systolic < 120 Diastolic < 80)
CNIDDM197	53	Female	Normal (Systolic < 120 Diastolic < 80)
CNIDDM198	43	Female	Normal (Systolic < 120 Diastolic < 80)
CNIDDM199	45	Male	Normal (Systolic < 120 Diastolic < 80)
CNIDDM200	52	Female	Normal (Systolic < 120 Diastolic < 80)
CNIDDM201	51	Male	Normal (Systolic < 120 Diastolic < 80)
CNIDDM202	39	Female	Normal (Systolic < 120 Diastolic < 80)
CNIDDM203	62	Female	Normal (Systolic < 120 Diastolic < 80)
CNIDDM204	60	Female	Normal (Systolic < 120 Diastolic < 80)
CNIDDM205	41	Female	Normal (Systolic < 120 Diastolic < 80)
CNIDDM206	55	Female	Normal (Systolic < 120 Diastolic < 80)
CNIDDM207	39	Male	Normal (Systolic < 120 Diastolic < 80)
CNIDDM208	60	Female	Normal (Systolic < 120 Diastolic < 80)
CNIDDM209	39	Female	Normal (Systolic < 120 Diastolic < 80)
CNIDDM210	42	Female	Normal (Systolic < 120 Diastolic < 80)
CNIDDM211	52	Female	Normal (Systolic < 120 Diastolic < 80)
CNIDDM212	42	Female	Normal (Systolic < 120 Diastolic < 80)
CNIDDM213	41	Male	Normal (Systolic < 120 Diastolic < 80)
CNIDDM214	43	Female	Normal (Systolic < 120 Diastolic < 80)
CNIDDM215	37	Female	Normal (Systolic < 120 Diastolic < 80)
CNIDDM216	43	Female	Normal (Systolic < 120 Diastolic < 80)
CNIDDM217	43	Female	Normal (Systolic < 120 Diastolic < 80)
CNIDDM218	53	Male	Normal (Systolic < 120 Diastolic < 80)
CNIDDM219	42	Male	Normal (Systolic < 120 Diastolic < 80)
CNIDDM220	44	Female	Normal (Systolic < 120 Diastolic < 80)
CNIDDM221	41	Male	Normal (Systolic < 120 Diastolic < 80)
CNIDDM222	63	Female	Normal (Systolic < 120 Diastolic < 80)
CNIDDM223	52	Male	Normal (Systolic < 120 Diastolic < 80)
CNIDDM224	43	Female	Normal (Systolic < 120 Diastolic < 80)
CNIDDM225	44	Female	Normal (Systolic < 120 Diastolic < 80)
CNIDDM226	44	Female	Normal (Systolic < 120 Diastolic < 80)
CNIDDM227	37	Female	Normal (Systolic < 120 Diastolic < 80)
CNIDDM228	64	Male	Normal (Systolic < 120 Diastolic < 80)
CNIDDM229	38	Female	Normal (Systolic < 120 Diastolic < 80)
CNIDDM230	57	Female	Normal (Systolic < 120 Diastolic < 80)
CNIDDM231	50	Female	Normal (Systolic < 120 Diastolic < 80)
CNIDDM232	55	Female	Normal (Systolic < 120 Diastolic < 80)

CNIDDM233	38	Female	Normal (Systolic < 120 Diastolic < 80)
CNIDDM234	41	Male	Normal (Systolic < 120 Diastolic < 80)
CNIDDM235	45	Female	Normal (Systolic < 120 Diastolic < 80)
CNIDDM236	54	Female	Normal (Systolic < 120 Diastolic < 80)
CNIDDM237	45	Female	Normal (Systolic < 120 Diastolic < 80)
CNIDDM238	53	Male	Normal (Systolic < 120 Diastolic < 80)
CNIDDM239	59	Female	Normal (Systolic < 120 Diastolic < 80)
CNIDDM240	63	Female	Normal (Systolic < 120 Diastolic < 80)
CNIDDM241	54	Female	Normal (Systolic < 120 Diastolic < 80)
CNIDDM242	46	Female	Normal (Systolic < 120 Diastolic < 80)
CNIDDM243	39	Female	Normal (Systolic < 120 Diastolic < 80)
CNIDDM244	65	Female	Normal (Systolic < 120 Diastolic < 80)
CNIDDM245	45	Male	Normal (Systolic < 120 Diastolic < 80)
CNIDDM246	43	Female	Normal (Systolic < 120 Diastolic < 80)
CNIDDM247	39	Male	Normal (Systolic < 120 Diastolic < 80)
CNIDDM248	43	Female	Normal (Systolic < 120 Diastolic < 80)
CNIDDM249	71	Male	Normal (Systolic < 120 Diastolic < 80)
CNIDDM250	44	Male	Normal (Systolic < 120 Diastolic < 80)
CNIDDM251	37	Female	Normal (Systolic < 120 Diastolic < 80)
CNIDDM252	46	Female	Normal (Systolic < 120 Diastolic < 80)
CNIDDM253	37	Female	Normal (Systolic < 120 Diastolic < 80)
CNIDDM254	64	Female	Normal (Systolic < 120 Diastolic < 80)
CNIDDM255	45	Female	Normal (Systolic < 120 Diastolic < 80)
CNIDDM256	42	Female	Normal (Systolic < 120 Diastolic < 80)
CNIDDM257	45	Male	Normal (Systolic < 120 Diastolic < 80)
CNIDDM258	38	Female	Normal (Systolic < 120 Diastolic < 80)
CNIDDM259	55	Female	Normal (Systolic < 120 Diastolic < 80)
CNIDDM260	38	Male	Normal (Systolic < 120 Diastolic < 80)
CNIDDM261	45	Male	Normal (Systolic < 120 Diastolic < 80)
CNIDDM262	52	Female	Normal (Systolic < 120 Diastolic < 80)
CNIDDM263	44	Male	Normal (Systolic < 120 Diastolic < 80)
CNIDDM264	53	Female	Normal (Systolic < 120 Diastolic < 80)
CNIDDM265	45	Female	Normal (Systolic < 120 Diastolic < 80)
CNIDDM266	51	Female	Normal (Systolic < 120 Diastolic < 80)
CNIDDM267	44	Male	Normal (Systolic < 120 Diastolic < 80)
CNIDDM268	39	Male	Normal (Systolic < 120 Diastolic < 80)
CNIDDM269	54	Female	Normal (Systolic < 120 Diastolic < 80)
CNIDDM270	44	Female	Normal (Systolic < 120 Diastolic < 80)
CNIDDM271	38	Female	Normal (Systolic < 120 Diastolic < 80)
CNIDDM272	42	Male	Normal (Systolic < 120 Diastolic < 80)
CNIDDM273	64	Female	Normal (Systolic < 120 Diastolic < 80)
CNIDDM274	66	Female	Normal (Systolic < 120 Diastolic < 80)
CNIDDM275	71	Female	Normal (Systolic < 120 Diastolic < 80)
CNIDDM276	65	Female	Normal (Systolic < 120 Diastolic < 80)
CNIDDM277	67	Female	Normal (Systolic < 120 Diastolic < 80)
CNIDDM278	56	Male	Normal (Systolic < 120 Diastolic < 80)
CNIDDM279	45	Female	Normal (Systolic < 120 Diastolic < 80)

CNIDDM280	39	Male	Normal (Systolic < 120 Diastolic < 80)
CNIDDM281	46	Female	Normal (Systolic < 120 Diastolic < 80)
CNIDDM282	36	Female	Normal (Systolic < 120 Diastolic < 80)
CNIDDM283	61	Male	Normal (Systolic < 120 Diastolic < 80)
CNIDDM284	59	Male	Normal (Systolic < 120 Diastolic < 80)
CNIDDM285	40	Female	Normal (Systolic < 120 Diastolic < 80)
CNIDDM286	36	Female	Normal (Systolic < 120 Diastolic < 80)
CNIDDM287	46	Female	Normal (Systolic < 120 Diastolic < 80)
CNIDDM288	37	Male	Normal (Systolic < 120 Diastolic < 80)
CNIDDM289	45	Female	Normal (Systolic < 120 Diastolic < 80)
CNIDDM290	38	Female	Normal (Systolic < 120 Diastolic < 80)
CNIDDM291	54	Female	Normal (Systolic < 120 Diastolic < 80)
CNIDDM292	37	Female	Normal (Systolic < 120 Diastolic < 80)
CNIDDM293	44	Male	Normal (Systolic < 120 Diastolic < 80)
CNIDDM294	42	Female	Normal (Systolic < 120 Diastolic < 80)
CNIDDM295	56	Female	Normal (Systolic < 120 Diastolic < 80)
CNIDDM296	53	Female	Normal (Systolic < 120 Diastolic < 80)
CNIDDM297	43	Female	Normal (Systolic < 120 Diastolic < 80)
CNIDDM298	53	Male	Normal (Systolic < 120 Diastolic < 80)
CNIDDM299	54	Female	Normal (Systolic < 120 Diastolic < 80)
CNIDDM300	55	Male	Normal (Systolic < 120 Diastolic < 80)
CNIDDM301	72	Male	Normal (Systolic < 120 Diastolic < 80)
CNIDDM302	54	Female	Normal (Systolic < 120 Diastolic < 80)
CNIDDM303	57	Female	Normal (Systolic < 120 Diastolic < 80)
CNIDDM304	56	Female	Normal (Systolic < 120 Diastolic < 80)
CNIDDM305	45	Female	Normal (Systolic < 120 Diastolic < 80)
CNIDDM306	39	Female	Normal (Systolic < 120 Diastolic < 80)
CNIDDM307	56	Female	Normal (Systolic < 120 Diastolic < 80)
CNIDDM308	40	Male	Normal (Systolic < 120 Diastolic < 80)
CNIDDM309	46	Female	Normal (Systolic < 120 Diastolic < 80)
CNIDDM310	60	Female	Normal (Systolic < 120 Diastolic < 80)
CNIDDM311	52	Male	Normal (Systolic < 120 Diastolic < 80)
CNIDDM312	40	Female	Normal (Systolic < 120 Diastolic < 80)
CNIDDM313	42	Female	Normal (Systolic < 120 Diastolic < 80)
CNIDDM314	55	Female	Normal (Systolic < 120 Diastolic < 80)
CNIDDM315	42	Male	Normal (Systolic < 120 Diastolic < 80)
CNIDDM316	38	Female	Normal (Systolic < 120 Diastolic < 80)
CNIDDM317	44	Female	Normal (Systolic < 120 Diastolic < 80)
CNIDDM318	60	Male	Normal (Systolic < 120 Diastolic < 80)
CNIDDM319	40	Male	Normal (Systolic < 120 Diastolic < 80)
CNIDDM320	52	Female	Normal (Systolic < 120 Diastolic < 80)
CNIDDM321	41	Male	Normal (Systolic < 120 Diastolic < 80)
CNIDDM322	41	Female	Normal (Systolic < 120 Diastolic < 80)
CNIDDM323	61	Male	Normal (Systolic < 120 Diastolic < 80)
CNIDDM324	59	Male	Normal (Systolic < 120 Diastolic < 80)
CNIDDM325	43	Female	Normal (Systolic < 120 Diastolic < 80)
CNIDDM326	54	Female	Normal (Systolic < 120 Diastolic < 80)

CNIDDM327	44	Female	Normal (Systolic < 120 Diastolic < 80)
CNIDDM328	65	Male	Normal (Systolic < 120 Diastolic < 80)
CNIDDM329	60	Female	Normal (Systolic < 120 Diastolic < 80)
CNIDDM330	51	Female	Normal (Systolic < 120 Diastolic < 80)
CNIDDM331	50	Male	Normal (Systolic < 120 Diastolic < 80)
CNIDDM332	36	Female	Normal (Systolic < 120 Diastolic < 80)
CNIDDM333	71	Female	Normal (Systolic < 120 Diastolic < 80)
CNIDDM334	41	Male	Normal (Systolic < 120 Diastolic < 80)
CNIDDM335	59	Female	Normal (Systolic < 120 Diastolic < 80)
CNIDDM336	63	Female	Normal (Systolic < 120 Diastolic < 80)
CNIDDM337	45	Female	Normal (Systolic < 120 Diastolic < 80)
CNIDDM338	57	Male	Normal (Systolic < 120 Diastolic < 80)
CNIDDM339	54	Female	Normal (Systolic < 120 Diastolic < 80)
CNIDDM340	41	Female	Normal (Systolic < 120 Diastolic < 80)
CNIDDM341	60	Male	Normal (Systolic < 120 Diastolic < 80)
CNIDDM342	51	Female	Normal (Systolic < 120 Diastolic < 80)
CNIDDM343	67	Female	Normal (Systolic < 120 Diastolic < 80)
CNIDDM344	59	Male	Normal (Systolic < 120 Diastolic < 80)
CNIDDM345	46	Male	Normal (Systolic < 120 Diastolic < 80)
CNIDDM346	57	Female	Normal (Systolic < 120 Diastolic < 80)
CNIDDM347	42	Female	Normal (Systolic < 120 Diastolic < 80)
CNIDDM348	54	Male	Normal (Systolic < 120 Diastolic < 80)
CNIDDM349	65	Male	Normal (Systolic < 120 Diastolic < 80)
CNIDDM350	60	Female	Normal (Systolic < 120 Diastolic < 80)
CNIDDM351	57	Female	Normal (Systolic < 120 Diastolic < 80)
CNIDDM352	51	Male	Normal (Systolic < 120 Diastolic < 80)
CNIDDM353	44	Male	Normal (Systolic < 120 Diastolic < 80)
CNIDDM354	50	Female	Normal (Systolic < 120 Diastolic < 80)
CNIDDM355	42	Female	Normal (Systolic < 120 Diastolic < 80)
CNIDDM356	57	Male	Normal (Systolic < 120 Diastolic < 80)
CNIDDM357	44	Male	Normal (Systolic < 120 Diastolic < 80)
CNIDDM358	68	Female	Normal (Systolic < 120 Diastolic < 80)
CNIDDM359	44	Male	Normal (Systolic < 120 Diastolic < 80)
CNIDDM360	37	Female	Normal (Systolic < 120 Diastolic < 80)
CNIDDM361	43	Male	Normal (Systolic < 120 Diastolic < 80)
CNIDDM362	55	Female	Normal (Systolic < 120 Diastolic < 80)
CNIDDM363	54	Female	Normal (Systolic < 120 Diastolic < 80)
CNIDDM364	69	Male	Normal (Systolic < 120 Diastolic < 80)
CNIDDM365	43	Female	Normal (Systolic < 120 Diastolic < 80)
CNIDDM366	52	Female	Normal (Systolic < 120 Diastolic < 80)
CNIDDM367	64	Female	Normal (Systolic < 120 Diastolic < 80)
CNIDDM368	46	Female	Normal (Systolic < 120 Diastolic < 80)
CNIDDM369	38	Female	Normal (Systolic < 120 Diastolic < 80)
CNIDDM370	52	Male	Normal (Systolic < 120 Diastolic < 80)
CNIDDM371	64	Female	Normal (Systolic < 120 Diastolic < 80)
CNIDDM372	52	Female	Normal (Systolic < 120 Diastolic < 80)
CNIDDM373	45	Female	Normal (Systolic < 120 Diastolic < 80)

CNIDDM374	65	Male	Normal (Systolic < 120 Diastolic < 80)
CNIDDM375	71	Female	Normal (Systolic < 120 Diastolic < 80)
CNIDDM376	41	Female	Normal (Systolic < 120 Diastolic < 80)
CNIDDM377	55	Female	Normal (Systolic < 120 Diastolic < 80)
CNIDDM378	50	Female	Normal (Systolic < 120 Diastolic < 80)
CNIDDM379	44	Male	Normal (Systolic < 120 Diastolic < 80)
CNIDDM380	38	Female	Normal (Systolic < 120 Diastolic < 80)
CNIDDM381	55	Female	Normal (Systolic < 120 Diastolic < 80)
CNIDDM382	41	Male	Normal (Systolic < 120 Diastolic < 80)
CNIDDM383	39	Female	Normal (Systolic < 120 Diastolic < 80)
CNIDDM384	72	Male	Normal (Systolic < 120 Diastolic < 80)
CNIDDM385	51	Female	Normal (Systolic < 120 Diastolic < 80)
CNIDDM386	55	Female	Normal (Systolic < 120 Diastolic < 80)
CNIDDM387	43	Male	Normal (Systolic < 120 Diastolic < 80)
CNIDDM388	42	Male	Normal (Systolic < 120 Diastolic < 80)
CNIDDM389	38	Female	Normal (Systolic < 120 Diastolic < 80)
CNIDDM390	57	Female	Normal (Systolic < 120 Diastolic < 80)
CNIDDM391	52	Female	Normal (Systolic < 120 Diastolic < 80)
CNIDDM392	37	Male	Normal (Systolic < 120 Diastolic < 80)
CNIDDM393	44	Female	Normal (Systolic < 120 Diastolic < 80)
CNIDDM394	46	Male	Normal (Systolic < 120 Diastolic < 80)
CNIDDM395	41	Male	Normal (Systolic < 120 Diastolic < 80)
CNIDDM396	65	Female	Normal (Systolic < 120 Diastolic < 80)
CNIDDM397	41	Female	Normal (Systolic < 120 Diastolic < 80)
CNIDDM398	44	Male	Normal (Systolic < 120 Diastolic < 80)
CNIDDM399	41	Female	Normal (Systolic < 120 Diastolic < 80)
CNIDDM400	44	Female	Normal (Systolic < 120 Diastolic < 80)
CNIDDM401	46	Female	Normal (Systolic < 120 Diastolic < 80)
CNIDDM402	44	Female	Normal (Systolic < 120 Diastolic < 80)
CNIDDM403	50	Male	Normal (Systolic < 120 Diastolic < 80)
CNIDDM404	62	Female	Normal (Systolic < 120 Diastolic < 80)
CNIDDM405	39	Female	Normal (Systolic < 120 Diastolic < 80)
CNIDDM406	40	Female	Normal (Systolic < 120 Diastolic < 80)
CNIDDM407	53	Female	Normal (Systolic < 120 Diastolic < 80)
CNIDDM408	67	Female	Normal (Systolic < 120 Diastolic < 80)
CNIDDM409	59	Female	Normal (Systolic < 120 Diastolic < 80)
CNIDDM410	52	Male	Normal (Systolic < 120 Diastolic < 80)
CNIDDM411	42	Female	Normal (Systolic < 120 Diastolic < 80)
CNIDDM412	52	Male	Normal (Systolic < 120 Diastolic < 80)
CNIDDM413	59	Male	Normal (Systolic < 120 Diastolic < 80)
CNIDDM414	36	Female	Normal (Systolic < 120 Diastolic < 80)
CNIDDM415	60	Female	Normal (Systolic < 120 Diastolic < 80)
CNIDDM416	59	Male	Normal (Systolic < 120 Diastolic < 80)
CNIDDM417	53	Female	Normal (Systolic < 120 Diastolic < 80)
CNIDDM418	40	Female	Normal (Systolic < 120 Diastolic < 80)
CNIDDM419	65	Male	Normal (Systolic < 120 Diastolic < 80)
CNIDDM420	72	Female	Normal (Systolic < 120 Diastolic < 80)

CNIDDM421	58	Male	Normal (Systolic < 120 Diastolic < 80)
CNIDDM422	46	Female	Normal (Systolic < 120 Diastolic < 80)
CNIDDM423	37	Female	Normal (Systolic < 120 Diastolic < 80)
CNIDDM424	69	Female	Normal (Systolic < 120 Diastolic < 80)
CNIDDM425	37	Male	Normal (Systolic < 120 Diastolic < 80)
CNIDDM426	44	Female	Normal (Systolic < 120 Diastolic < 80)
CNIDDM427	62	Male	Normal (Systolic < 120 Diastolic < 80)
CNIDDM428	60	Female	Normal (Systolic < 120 Diastolic < 80)
CNIDDM429	60	Female	Normal (Systolic < 120 Diastolic < 80)
CNIDDM430	45	Female	Normal (Systolic < 120 Diastolic < 80)
CNIDDM431	54	Female	Normal (Systolic < 120 Diastolic < 80)
CNIDDM432	50	Male	Normal (Systolic < 120 Diastolic < 80)
CNIDDM433	68	Female	Normal (Systolic < 120 Diastolic < 80)
CNIDDM434	55	Male	Normal (Systolic < 120 Diastolic < 80)
CNIDDM435	53	Female	Normal (Systolic < 120 Diastolic < 80)
CNIDDM436	43	Female	Normal (Systolic < 120 Diastolic < 80)
CNIDDM437	51	Male	Normal (Systolic < 120 Diastolic < 80)
CNIDDM438	41	Male	Normal (Systolic < 120 Diastolic < 80)
CNIDDM439	41	Female	Normal (Systolic < 120 Diastolic < 80)
CNIDDM440	37	Female	Normal (Systolic < 120 Diastolic < 80)
CNIDDM441	40	Male	Normal (Systolic < 120 Diastolic < 80)
CNIDDM442	41	Male	Normal (Systolic < 120 Diastolic < 80)
CNIDDM443	51	Female	Normal (Systolic < 120 Diastolic < 80)
CNIDDM444	63	Female	Normal (Systolic < 120 Diastolic < 80)
CNIDDM445	50	Female	Normal (Systolic < 120 Diastolic < 80)
CNIDDM446	44	Male	Normal (Systolic < 120 Diastolic < 80)
CNIDDM447	53	Female	Normal (Systolic < 120 Diastolic < 80)
CNIDDM448	53	Female	Normal (Systolic < 120 Diastolic < 80)
CNIDDM449	57	Female	Normal (Systolic < 120 Diastolic < 80)
CNIDDM450	67	Female	Normal (Systolic < 120 Diastolic < 80)
CNIDDM451	59	Male	Normal (Systolic < 120 Diastolic < 80)
CNIDDM452	59	Female	Normal (Systolic < 120 Diastolic < 80)
CNIDDM453	40	Female	Normal (Systolic < 120 Diastolic < 80)
CNIDDM454	37	Female	Normal (Systolic < 120 Diastolic < 80)
CNIDDM455	44	Female	Normal (Systolic < 120 Diastolic < 80)
CNIDDM456	50	Male	Normal (Systolic < 120 Diastolic < 80)
CNIDDM457	43	Male	Normal (Systolic < 120 Diastolic < 80)
CNIDDM458	61	Female	Normal (Systolic < 120 Diastolic < 80)
CNIDDM459	53	Male	Normal (Systolic < 120 Diastolic < 80)
CNIDDM460	51	Male	Normal (Systolic < 120 Diastolic < 80)
CNIDDM461	48	Female	Normal (Systolic < 120 Diastolic < 80)
CNIDDM462	48	Female	Normal (Systolic < 120 Diastolic < 80)
CNIDDM463	38	Male	Normal (Systolic < 120 Diastolic < 80)
CNIDDM464	44	Female	Normal (Systolic < 120 Diastolic < 80)
CNIDDM465	55	Female	Normal (Systolic < 120 Diastolic < 80)
CNIDDM466	44	Male	Normal (Systolic < 120 Diastolic < 80)
CNIDDM467	55	Female	Normal (Systolic < 120 Diastolic < 80)

CNIDDM468	36	Female	Normal (Systolic < 120 Diastolic < 80)
CNIDDM469	43	Female	Normal (Systolic < 120 Diastolic < 80)
CNIDDM470	44	Female	Normal (Systolic < 120 Diastolic < 80)
CNIDDM471	54	Female	Normal (Systolic < 120 Diastolic < 80)
CNIDDM472	52	Female	Normal (Systolic < 120 Diastolic < 80)
CNIDDM473	42	Male	Normal (Systolic < 120 Diastolic < 80)
CNIDDM474	40	Female	Normal (Systolic < 120 Diastolic < 80)
CNIDDM475	40	Female	Normal (Systolic < 120 Diastolic < 80)
CNIDDM476	65	Female	Normal (Systolic < 120 Diastolic < 80)
CNIDDM477	68	Female	Normal (Systolic < 120 Diastolic < 80)
CNIDDM478	38	Male	Normal (Systolic < 120 Diastolic < 80)
CNIDDM479	71	Male	Normal (Systolic < 120 Diastolic < 80)
CNIDDM480	46	Female	Normal (Systolic < 120 Diastolic < 80)
CNIDDM481	57	Female	Normal (Systolic < 120 Diastolic < 80)
CNIDDM482	43	Female	Normal (Systolic < 120 Diastolic < 80)
CNIDDM483	55	Female	Normal (Systolic < 120 Diastolic < 80)
CNIDDM484	70	Male	Normal (Systolic < 120 Diastolic < 80)
CNIDDM485	43	Female	Normal (Systolic < 120 Diastolic < 80)
CNIDDM486	41	Male	Normal (Systolic < 120 Diastolic < 80)
CNIDDM487	52	Female	Normal (Systolic < 120 Diastolic < 80)
CNIDDM488	37	Female	Normal (Systolic < 120 Diastolic < 80)
CNIDDM489	62	Male	Normal (Systolic < 120 Diastolic < 80)
CNIDDM490	67	Female	Normal (Systolic < 120 Diastolic < 80)
CNIDDM491	46	Female	Normal (Systolic < 120 Diastolic < 80)
CNIDDM492	67	Female	Normal (Systolic < 120 Diastolic < 80)
CNIDDM493	38	Male	Normal (Systolic < 120 Diastolic < 80)
CNIDDM494	53	Female	Normal (Systolic < 120 Diastolic < 80)
CNIDDM495	44	Female	Normal (Systolic < 120 Diastolic < 80)
CNIDDM496	43	Female	Normal (Systolic < 120 Diastolic < 80)
CNIDDM497	43	Female	Normal (Systolic < 120 Diastolic < 80)
CNIDDM498	39	Male	Normal (Systolic < 120 Diastolic < 80)
CNIDDM499	39	Female	Normal (Systolic < 120 Diastolic < 80)
CNIDDM500	38	Female	Normal (Systolic < 120 Diastolic < 80)
