# Supplementary figures and images for: Linkage disequilibrium block single-nucleotide polymorphisms in FTO alpha ketoglutarate dependent dioxygenase gene inference with breast cancer and Type II diabetes in Pakistani female population
Source: PLoS One. 2023 Jul 20;18(7):e0288934. doi: 10.1371/journal.pone.0288934 (PMC10358933; doi:10.1371/journal.pone.0288934)

## S1\_raw\_images

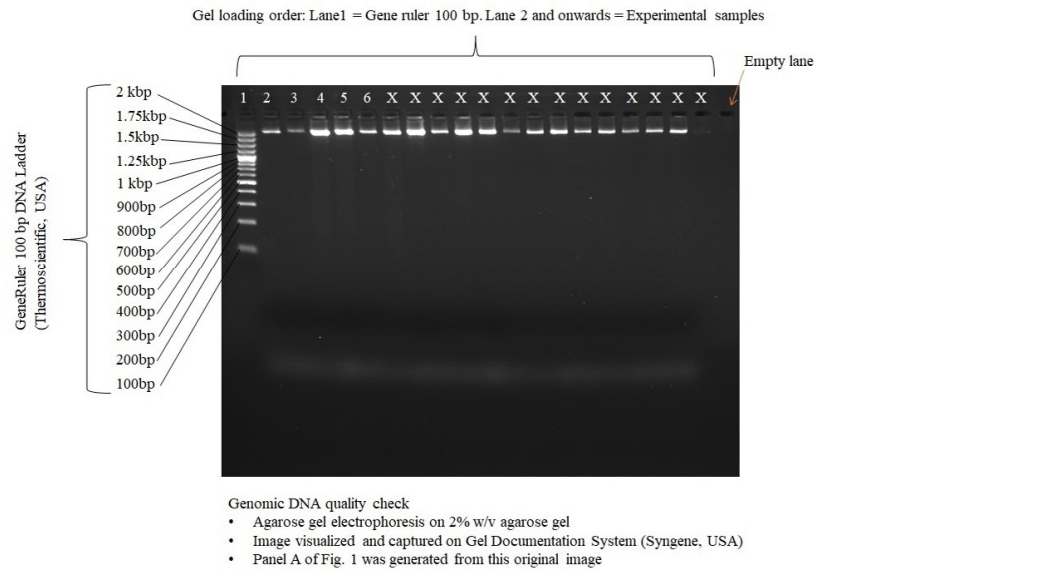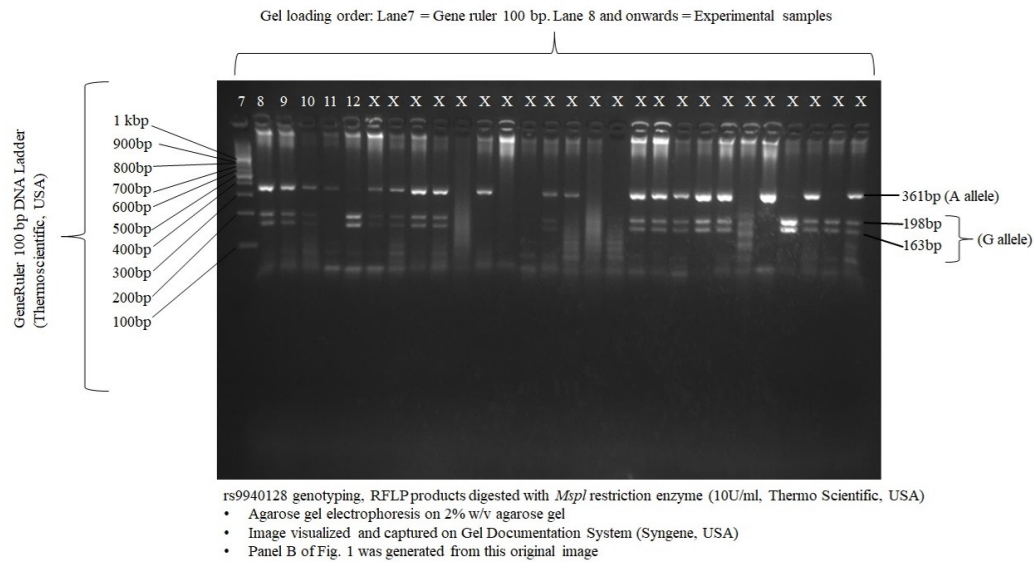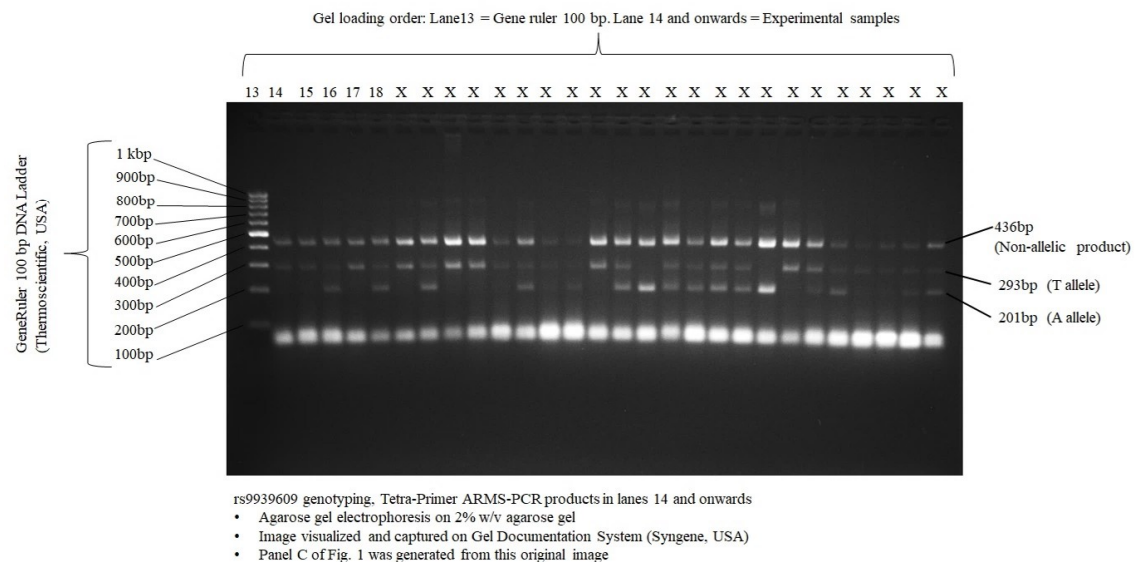

Supplement: S1 Raw images — (PDF) [file pone.0288934.s005.pdf]
